# Non‐genetic biomarkers and colorectal cancer risk: Umbrella review and evidence triangulation

**DOI:** 10.1002/cam4.3051

**Published:** 2020-05-12

**Authors:** Xiaomeng Zhang, Dipender Gill, Yazhou He, Tian Yang, Xue Li, Grace Monori, Harry Campbell, Malcolm Dunlop, Konstantinos K. Tsilidis, Maria Timofeeva, Evropi Theodoratou

**Affiliations:** ^1^ Centre for Global Health Usher Institute The University of Edinburgh Edinburgh UK; ^2^ Department of Epidemiology and Biostatistics School of Public Health Imperial College London London UK; ^3^ Colon Cancer Genetics Group Medical Research Council Human Genetics Unit Institute of Genetics and Molecular Medicine Western General Hospital University of Edinburgh Edinburgh UK; ^4^ Department of Hygiene and Epidemiology University of Ioannina School of Medicine Ioannina Greece; ^5^ Danish Institute for Advanced Study Department of Public Health University of Southern Denmark Odense C Denmark

**Keywords:** biomarkers, cancer risk factors, colorectal cancer, epidemiology and prevention

## Abstract

Several associations between non‐genetic biomarkers and colorectal cancer (CRC) risk have been detected, but the strength of evidence and the direction of associations are not confirmed. We aimed to evaluate the evidence of these associations and integrate results from different approaches to assess causal inference. We searched Medline and Embase for meta‐analyses of observational studies, meta‐analyses of randomized clinical trials (RCTs), and Mendelian randomization (MR) studies measuring the associations between non‐genetic biomarkers and CRC risk and meta‐analyses of RCTs on supplementary micronutrients. We repeated the meta‐analyses using random‐effects models and categorized the evidence based on predefined criteria. We described each MR study and evaluated their credibility. Seventy‐two meta‐analyses of observational studies and 18 MR studies on non‐genetic biomarkers and six meta‐analyses of RCTs on micronutrient intake and CRC risk considering 65, 42, and five unique associations, respectively, were identified. No meta‐analyses of RCTs on blood level biomarkers have been found. None of the associations were classified as convincing or highly suggestive, three were classified as suggestive, and 26 were classified as weak. For three biomarkers explored in MR studies, there was evidence of causality and seven were classified as likely noncausal. For the first time, results from both observational and MR studies were integrated by triangulating the evidence for a wide variety of non‐genetic biomarkers and CRC risk. At blood level, lower vitamin D, higher homeostatic model assessment‐insulin resistance, and human papillomavirus infection were associated with higher CRC risk while increased linoleic acid and oleic acid and decreased arachidonic acid were likely causally associated with lower CRC risk. No association was found convincing in both study types.

## Graphical abstract

The identification of specific biomarkers related to colorectal cancer (CRC) risk is essential to understand cancer aetiology and mechanisms of progression. This is the first time that evidence of associations between a wide range of non‐genetic biomarkers and CRC risk from a huge number of epidemiological workswere evaluated. Three associations detected from meta‐analyses of observational studies were classified as suggestive, three and seven associations detected from Mendelian randomisation studies were classified as evidence of causality and likely non‐causal respectively.

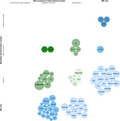

## 1 INTRODUCTION

Colorectal cancer (CRC) is the third most common cancer, and the second leading cause of cancer death globally.^1^ More than 1.8 million new cases and 881 000 deaths were estimated to have occurred in 2018.^1^ Furthermore, although there are stable or descending trends in many high‐income countries, their age‐specific incidence and mortality rates remain among the highest in the world, especially the incidence among young adults.^2^, ^3^

A biomarker is defined as a cellular, biochemical, or molecular alteration that can be measured and is used to objectively evaluate normal biological or pathological processes.^4^ Different types of biomarkers have been investigated in relation to CRC risk. Environmental factors play an important role in the etiology of CRC through modulating differentiation, apoptosis, angiogenesis, proliferation, and immune processes against endothelial cells.^5^ Identifying specific biomarkers related to CRC risk is important for understanding cancer etiology and mechanisms of progression as well as early detection and cancer screening that could consequently reduce CRC mortality. The aims of this review were: (a) to identify meta‐analyses of observational studies, meta‐analyses of randomized clinical trials (RCTs), and Mendelian randomization (MR) studies on non‐genetic biomarkers and CRC risk; (b) to evaluate the observed associations and classify the level of credibility of the evidence; and (c) to integrate the evidence across different approaches using an evidence triangulation framework. Genetic risk factors have been recently explored in a number of field synopses^6^, ^7^ and meta‐analyses of genome wide association studies,^8^, ^9^and are not considered in this work.

## 2 METHODS

### 2.1 Search strategy and eligibility criteria

Two reviewers searched Medline and Embase to identify meta‐analyses of observational studies (1 January 2010 to 14 June 2019), meta‐analyses of RCTs (1 January 2010 to 14 June 2019), and MR studies (up to 20 June 2019) investigating the association between non‐genetic biomarkers and CRC risk. As no meta‐analyses of RCTs on non‐genetic biomarkers were identified, we included meta‐analyses of RCTs (1 January 2010 to 14 June 2019) on micronutrient intake as proxies of micronutrient blood levels. Systematic reviews without meta‐analyses were excluded. Meta‐analyses of observational studies on non‐genetic biomarkers and CRC risk published before 2010 had been previously reviewed in a published umbrella review.^10^ The main results of these studies were extracted from the published umbrella review and were further evaluated and assessed together with additional studies published from 2010 onwards. A parallel review was conducted by a third reviewer. In the case of any discrepancy in assessments, a final decision was made after discussion. The details of all search strategies are provided in Table S1. We first reviewed the title and abstract of the identified studies and then evaluated the full text of all potential eligible studies. We manually checked the references of all retrieved articles to include any missed relevant studies. Studies investigating the associations between genetic or non‐genetic biomarkers and CRC screening, diagnosis, survival, and prognosis were excluded.

### 2.2 Data extraction

One investigator extracted information from each eligible study and two other investigators checked the extracted data. A fourth investigator was involved to judge any discrepancies. For meta‐analyses of observational studies, we extracted the first author, year of publication, number of studies considered, epidemiological study design, biomarker details, outcome, and study population. We also recorded the study‐specific relative risk estimates (risk ratio, odds ratio, hazard ratio, standardized mean difference, weighted mean difference, standardized correlation coefficient), details of the applied statistical models, correspondent confidence intervals, and number of cases and participants. For meta‐analyses of RCTs on micronutrient intake, we further extracted the dose and duration of supplementation, number of events, and type of intervention in the control group. For MR studies, we extracted: the exposure, study design, effect estimate unit, sample size, population ethnicity for both exposure and outcome groups, main MR estimate and any sensitivity analyses for the associations of genetic instruments with the exposure and outcome, total variance level explained by the genetic instrument assuming an additive model (*R* ^2^), and the approximate statistical power (where presented).

### 2.3 Statistical analysis

For the meta‐analyses of observational studies, we re‐estimated the summary effect size and its confidence interval. As the most commonly used DerSimonian and Laird (DL) estimator tends to underestimate the 95% CI when less than 10 studies are included,^11^ we used the Hartung‐Knapp‐Sidik‐Jonkman (HKSJ) method as the main random effect estimator.^12^ The HKSJ estimator consistently results in more adequate error rates even when the number of studies is small or between studies heterogeneity exists.^12^, ^13^ The meta‐analysis *P* value threshold was set at .05. The Paule‐Mandel (PM) estimator could give an accurate result when between‐study heterogeneity is large but the number of studies is not small.^13^ Therefore, DL^14^ and PM^15^ methods were also applied as sensitivity analyses. We quantified the heterogeneity of each meta‐analysis by calculating the I^2^ value and its 95% prediction interval.^16^, ^17^ We used the Egger regression asymmetry test to estimate any small study effect.^18^ The excess significance test was performed to evaluate whether the observed number of studies with positive results was significantly greater than the expected number by using a chi‐square test.^19^ For both the small study effect and the excess significance test, we used *P* < .1 as the threshold.

Stata version 14.0 and “metafor” package^20^

in R 3.5.1 were used for statistical analysis. Two‐tailed *P* values were used.

### 2.4 Credibility assessment

If there were more than one meta‐analysis of observational studies or more than one MR study investigating the association between the same biomarker and CRC risk, we compared the direction, level of statistical significance (*P* ≤ .05), and effect size. The most recent meta‐analysis with the largest number of prospective studies was retained for further analysis. The most recent MR study (unless a previous MR study employed a stronger genetic instrument and/ or had a larger sample size at the outcome arm) was retained for further comparison.

If we identified meta‐analyses of observational studies and MR studies investigating the same biomarker, we compared the direction and level of statistical significance (*P* ≤ .05). All associations explored in meta‐analyses of observational studies and/ or MR studies are presented in an evidence triangulation plot.^21^, ^22^

We categorized the evidence from meta‐analyses of observational studies for each eligible biomarker in four categories according to previously defined criteria that considered the quantified evidence, statistical significance, heterogeneity, small study effect, excess significance bias, and prediction interval (convincing or class I, highly suggestive or class II, suggestive or class III, weak or class IV, and no association).^23^ The evidence classification criteria are described in Table 1. For each convincing or highly suggestive association, we rechecked the eligibility for each individual study, re‐evaluated the accuracy of extracted data, and reassessed the evidence after restricting the analysis to prospective cohort studies.

**TABLE 1:** Credibility assessment criteria for meta‐analyses of observational studies and Mendelian randomization studies

Evidence category	Criteria
Meta‐analyses of observational studies	
Convincing (class I)	*P* < .001; >1000 cases; *P* < .05 in the largest study A 95% PI that excluded the null; I^2^ < 50% No evidence of small‐study effect (*P* > .10); and no excess significance bias (*P* > .10)
Highly suggestive (class II)	*P* < .001; >1000 cases *P* < .05 in the largest study
Suggestive (class III)	*P* < .001; >1000 cases
Weak (class IV)	*P* < .05
No association	*P* ≥ .05
Mendelian randomization study	
Evidence of causality	*P* < .05 or threshold set up by individual study due to multiple testing; evaluated pleiotropy but have no evidence of directional pleiotropy.
Likely noncausal	*P* > .05 or threshold setup by individual study due to multiple testing; Power ≥0.8; consistent evidence between main MR analysis and sensitivity analyses; evaluated pleiotropy but have no evidence of directional pleiotropy
Unknown	Studies that cannot be classified as either “Evidence of causality” or “Likely noncausal”

Abbreviation: PI: prediction interval.

Associations detected from MR studies were categorized into “Evidence of causality,” “Likely noncausal,” and “Unknown” by considering statistical significance (*P* < .05), pre‐estimated power (Power ≥ 0.8 regarded as sufficient), and evidence of bias due to directional pleiotropy (Table 1).

## 3 RESULTS

**FIGURE 1:**
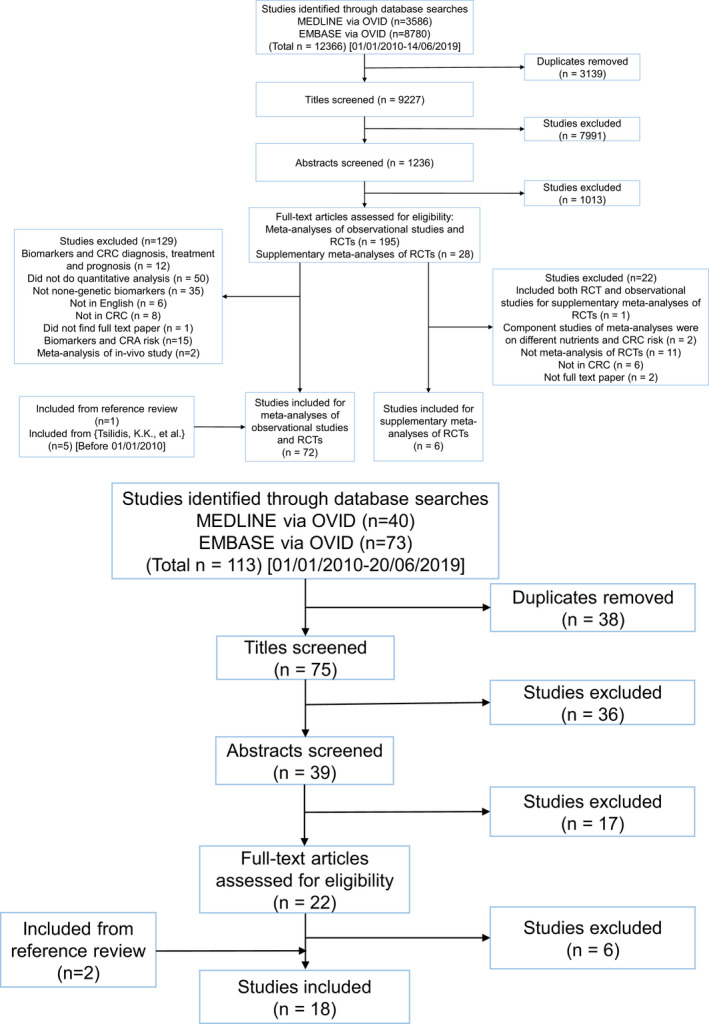
A, PRISMA flow diagram illustrating the study screening and selection process for meta‐analyses of observational studies and meta‐analyses of randomized clinical trials (performed on 14/06/2019). B, PRISMA flow diagram illustrating the study screening and selection process for Mendelian randomization studies (performed on 20/06/2019)

The literature search returned 9227 hits for the meta‐analyses of observational studies and RCTs, and returned 75 hits for MR studies. After applying the predefined inclusion and exclusion criteria, 72 meta‐analyses of observational studies, 18 MR studies, and six meta‐analyses of RCTs on supplementary micronutrients were identified (Figure 1).

### 3.1 Meta‐analyses of observational studies

A total of 145 effect estimates for 65 unique biomarkers were extracted from the 72 included meta‐analyses of observational studies (Table S4). The median number of included component studies for each meta‐analysis was 7 (range: 2‐31). The median number of cases was 1170 (range: 37‐62 814) and of participants was 4240 (range: 76‐7 725 310). More than one meta‐analysis of observational studies was identified for 20 biomarkers (Table S6): helicobacter pylori infection (H. pylori, n = 9), human papillomavirus infection (HPV, n = 8), blood levels of folate (n = 6), blood levels of vitamin B12 (n = 5), blood levels of vitamin B6 (n = 5), blood levels of vitamin B2 (n = 2), blood levels of 25‐hydroxyvitamin D (n = 10), C‐reactive protein (CRP, n = 3), interleukin‐6 (IL‐6, n = 2), fasting glucose (n = 6), C peptide (n = 3), insulin‐like growth factor‐1 (IGF‐1; n = 3), IGF‐2 (n = 2), insulin‐like growth factor‐binding protein 3 (IGFBP‐3, n = 2), triglycerides (n = 3), high‐density lipoprotein cholesterol (HDL‐cholesterol, n = 2), adiponectin (n = 7), leptin (n = 4), telomere length (n = 3), and homocysteine (n = 3). Seventeen of the 20 (85%) overlapping meta‐analyses agreed on the direction of the effect estimate, 12 of these 17 agreed on the level of statistical significance, and 10 of these 12 associations were statistically significant (Table S6).

After removing the overlapping meta‐analyses, a total of 65 unique biomarkers were retained for further statistical analysis (Figure 3; Tables S2 and S7). We categorized the biomarkers into seven categories: fatty acid/lipid metabolism biomarkers (n = 14), micronutrients (n = 10), infectious agents (n = 13), inflammatory markers (n = 2), insulin‐related biomarkers (n = 10), protein/amino acids (n = 10), and other biomarkers (n = 6).

**FIGURE 2:**
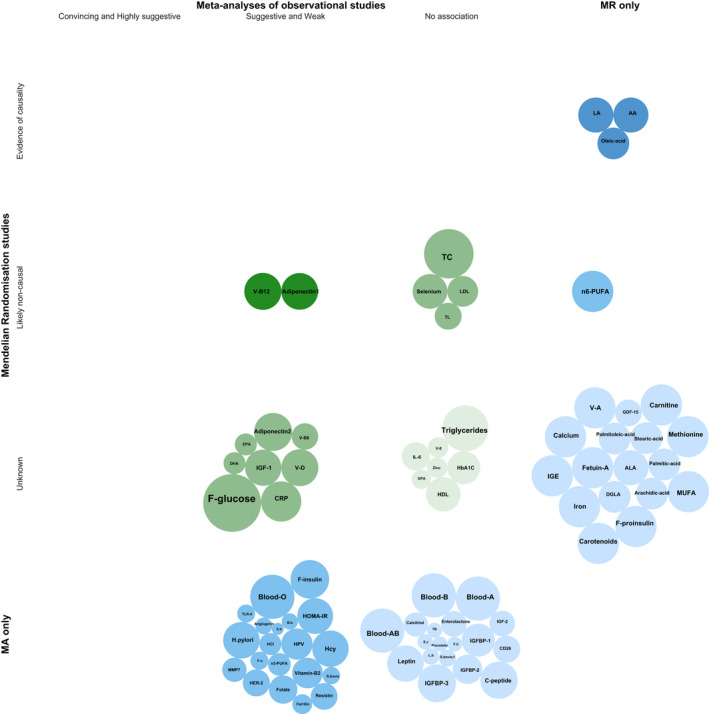
Evidence triangulation bubble plot for biomarkers detected from meta‐analyses of observational studies and MR studies. The bubble size of meta‐analyses of observational studies represents the number of cases and the bubble size of MR studies represents the number of CRC cases divided by 5. AA, arachidonic acid; Adiponectin1, Adiponectin in European and United State population; Adiponectin2, Adiponectin in European population only; ALA, α‐Linolenic acid; B.b, bifidobacterium; Blood‐A/B/AB/O, Blood group A/B/AB/O; CD26, dipeptidyl peptidase IV; CRP, C‐reactive protein; DGLA, dihomo‐γ‐linolenic acid; DHA, Docosahexaenoic acid; DPA, Docosapentaenoic acid; E.b, enterobacteriaceae; E.c, escherichia coli; EPA, Eicosapentaenoic acid; F.b, faecalibacterium prausnitzii; F‐glucose, fasting glucose; F‐insulin, fasting insulin; F.n, F. nucleatum; F‐proinsulin, fasting proinsulin; GDF‐15, Growth differentiation factor 15; HbA1C, glycated hemoglobin; HCI, human cytomegalovirus infection; Hcy, homocysteine; HDL, high‐density lipoprotein cholesterol; HOMA‐IR, homeostatic model assessment‐insulin resistance; HPV, Human papillomavirus; H.pylori, helicobacter pylori; IGE, serum immunoglobulin E; IGF‐1/2, insulin‐like growth factor 1/2; IGFBP 1/2/3, Insulin‐like growth factor‐binding protein 1/2/3; IL‐6, interleukin 6; LA, linoleic acid; L.b, lactobacillus; LDL, low‐density lipoprotein cholesterol; MA only, Biomarkers only detected in meta‐analyses of observational studies; MMP7, matrix metalloproteinase‐7; MR only, Biomarkers only detected in MR studies; MUFA, mono‐unsaturated fatty acids; n‐3 PUFA, long chain n‐3 polyunsaturated fatty acid; n‐6 PUFA, n‐6 polyunsaturated fatty acid; S.bovis, streptococcus bovis; S.bovis.f, Streptococcus bovis in feces; TB, total bacteria; TC, total cholesterol; TL, telomere length; V‐B12/B6/D/E/A, Vitamin B12/B6/D/E/A

**FIGURE 3:**
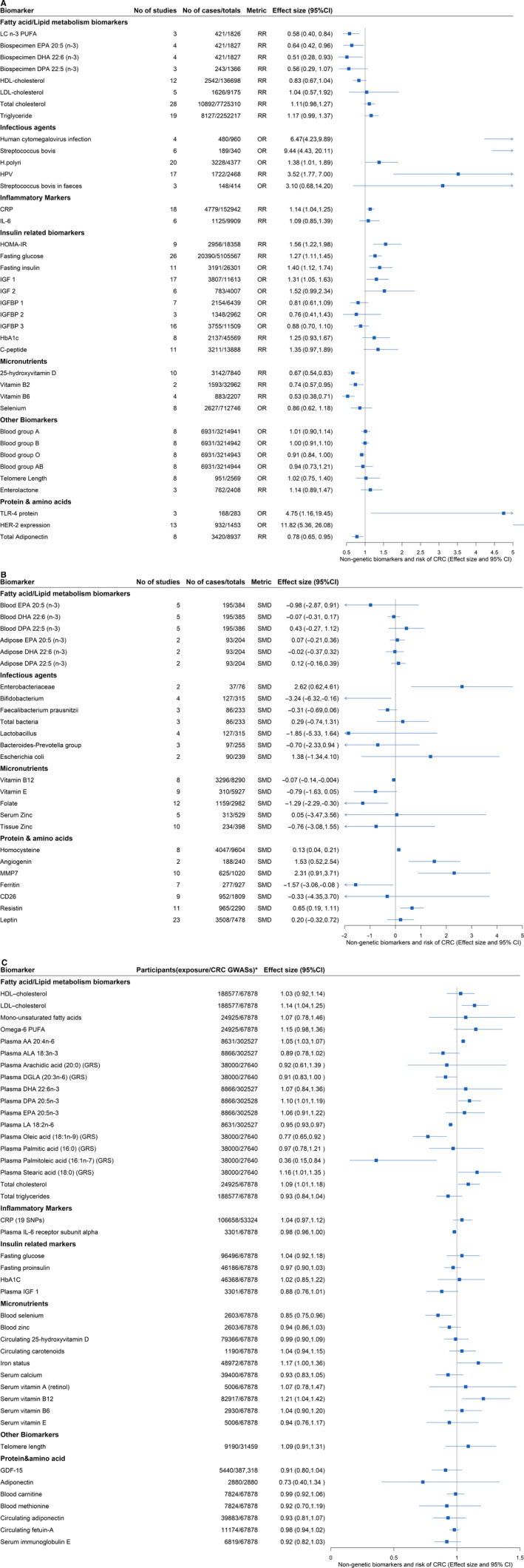
A, Forest plot for evidence of associations between non‐genetic biomarkers and CRC risk from meta‐analyses of observational studies (metric: odds ratio and risk ratio). B, Forest plot for evidence of associations between non‐genetic biomarkers and CRC risk from meta‐analyses of observational studies (metric: standardized mean difference). C, Forest plot for evidence of associations between non‐genetic biomarkers and CRC risk from MR studies (metric: odds ratio). *, total number of participants for exposure and CRC Genome‐wide association studies; AA, arachidonic acid; ALA, α‐Linolenic acid; CD26, dipeptidyl peptidase IV; CI, confidence interval, results of meta‐analyses were analyzed by using Hartung‐Knapp‐Sidik‐Jonkman method; CRC, colorectal cancer; CRP, C‐reactive protein; DGLA, dihomo‐γ‐linolenic acid; DHA, Docosahexaenoic acid; DPA, Docosapentaenoic acid; EPA, Eicosapentaenoic acid; GDF‐15, Growth differentiation factor 15; GRS, genetic risk score; H.pylori, Helicobacter pylori; HbA1c, glycated hemoglobin; HDL, high‐density lipoprotein cholesterol; HOMA‐IR, homeostatic model assessment‐insulin resistance; HPV, Human papillomavirus; IGF 1/2, Insulin‐like growth factor 1/2; IGFBP 1/2/3, Insulin‐like growth factor‐binding protein 1/2/3; IL‐6, Interleukin 6; LA, linoleic acid; LC n‐3 PUFA, long chain n‐3 polyunsaturated fatty acid; LDL, low‐density lipoprotein cholesterol; MMP7, matrix metalloproteinase‐7; OR, odds ratio; RR, risk ratio; SMD, standardized mean difference; SNP, Single‐Nucleotide Polymorphism

A total of 29 associations among the 65 non‐overlapping meta‐analyses of observational studies (45%) were statistically significant (*P* < .05) by using the HKSJ meta‐analysis estimator (Table S2; Figures 2 and 3). Sensitivity analyses using the DL and PM estimator are presented in Table S7. Eight and five associations were upgraded when using DL estimator or the PM estimator instead of the HKSJ estimator, respectively. Sixteen of the 29 significant biomarkers were associated with increased CRC risk. In these 29 statistically significant associations, 7 (24%) had *P* < .001, 24 (83%) had a 95% prediction interval that excluded null, 14 (48%) had more than 1000 cases, 13 (45%) had no obvious large heterogeneity (I^2^ < 50%), 20 (69%) were not subject to small‐study effect or excess significance bias (Table S2). After applying the credibility criteria, one biomarker (fasting glucose [RR (95% CI): 1.27 (1.11, 1.45)]) was classified as highly suggestive and three biomarkers were classified as suggestive (homeostatic model assessment‐insulin resistance [HOMA‐IR; RR (95% CI): 1.56(1.22, 1.98)], 25‐hydroxyvitamin D [RR (95% CI): 0.67(0.54, 0.83)], and HPV [RR (95% CI): 3.52(1.77, 7.00)]). For the associations classified as “highly suggestive,” we checked the eligibility of each component study, evaluated the accuracy of the extracted data and reassessed the evidence after restricting the analysis to only including prospective studies. The evidence of association between fasting glucose and CRC risk was downgraded to “weak.”

We identified six meta‐analyses of RCTs on associations between supplementary micronutrients and CRC risk, but none of them reported a statistically significant association (Table S8).

### 3.2 Mendelian randomization studies

Sixty‐six MR studies were extracted from 18 publications (Table S5). The median number of cases for the outcome arm of each included MR study was 13 012 (range: 329‐30 480), the median number of participants was 36 137 (range: 727‐382 756), and the median variance explained by each genetic instrument was 2.92% (range: 0.3%‐60.4%). Eight (12%) MR studies had enough power (≥0.8) to detect a statistically significant effect estimate. Overlapping MR studies were detected for 14 biomarkers (Table S6). Nine of the 14 MR studies agreed on the direction of the effect size and eight of which agreed on the level of statistical significance: overlapping MR studies for plasma arachidonic acid (n = 2) and plasma linoleic acid (n = 2) agreed on the direction of effect size and the effect size estimates were statistically significant; overlapping MR studies for adiponectin (n = 3), fetuin‐A (n = 2), docosapentaenoic acid (DPA, n = 2), docosahexaenoic acid (DHA; n = 2), low‐density lipoprotein cholesterol (LDL‐cholesterol, n = 3), and telomere length (n = 2) were concordant in the direction, but the effect size estimates were not statistically significant; overlapping MR studies for total cholesterol (n = 2) agreed on the direction but not on the level of statistical significance; MR studies for blood levels of 25‐hydroxyvitamin D (n = 8), eicosapentaenoic acid (EPA; n = 2), triglyceride (n = 3), HDL‐cholesterol (n = 3), and CRP (n = 3) neither agreed on direction nor on statistical significance.

The biomarkers of the 66 MR analyses were categorized into six categories: micronutrients (n = 17), fatty acid/lipid metabolism biomarkers (n = 30), inflammatory markers (n = 4), protein/amino acid (n = 9), insulin‐related markers (n = 4), and other biomarkers (n = 2) (Table S5). Nine (14%) biomarkers (stearic acid, arachidonic acid [n = 2], linoleic acid [n = 2], oleic acid, palmitoleic acid, total cholesterol, and CRP) reported statistically significant associations (at *P* < .05 or at a study specified threshold due to multiple testing). After removing the overlapping MR studies, 42 biomarkers were retained for analysis (Table S3; Figure 3), three biomarkers (arachidonic acid [OR (95% CI): 1.05 (1.03, 1.07)], linoleic acid [OR (95% CI): 0.95 (0.93, 0.97)], and oleic acid [OR (95% CI): 0.77 (0.65, 0.92)]) with statistically significant effect estimates and without evidence of biological pleiotropy were classified as having “Evidence of causality”. Seven MR studies were categorized as “Likely noncausal,” since these studies had enough statistical power and no evidence of biological pleiotropy, but they were statistically nonsignificant (LDL–cholesterol, omega‐6 polyunsaturated fatty acids, total cholesterol, selenium, vitamin B12, telomere length, adiponectin).

Twenty non‐genetic biomarkers were analyzed in both meta‐analyses of observational studies and MR studies (Table S6; Figure 2). Ten of the 20 biomarkers (25‐hydroxyvitamin D, selenium, vitamin E, total cholesterol, LDL‐cholesterol, CRP, fasting glucose, glycated hemoglobin [HbA1C], adiponectin, telomere length) agreed on the direction of the effect size, six (selenium, vitamin E, total cholesterol, LDL‐cholesterol, HbA1C, telomere length) of which agreed on the level of statistical significance (not significant). One biomarker (25‐hydroxyvitamin D) was analyzed by three different study types (meta‐analysis of observational study, MR studies, and meta‐analysis of RCTs on supplementary vitamin D), but only the meta‐analyses of observational studies reported a statistically significant association.

## 4 DISCUSSION

In this study, a comprehensive overview of associations between a wide range of non‐genetic biomarkers and CRC risk was conducted by triangulating evidence from meta‐analyses of observational studies, MR studies, and meta‐analyses of RCTs. The non‐genetic biomarkers for CRC risk which were studied covered seven categories and CRC risk was associated with 34 examined biomarkers. There is a gap of meta‐analyses of RCTs or even individual RCTs on biomarkers of CRC risk and these were only examined in observational studies. We, therefore, included meta‐analyses of RCTs of supplementary micronutrients as proxies.

### 4.1 Meta‐analyses of observational studies

Twenty‐nine biomarkers were associated with CRC risk at *P* < .05 from meta‐analyses of observational studies, but none of these association was classified as convincing or highly suggestive. Of these 29 statistically significant associations, three (25‐hydroxyvitamin D, HPV, and HOMA‐IR [HOMA‐IR = glucose ×insulin/405]) were classified as suggestive and 26 as weak.

The association between vitamin D concentration and CRC risk was classified as suggestive (Class III) and indicated that a higher blood concentration of vitamin D was associated with a 33% decrease in CRC risk. This result was consistent among all eight overlapping meta‐analyses.^24^, ^25^ Experimental studies based on mouse models have indicated that the potent steroid hormone Calcitriol (the active form of vitamin D) may play a protective role against CRC through the regulation of proliferation, pro‐differentiation, pro‐apoptosis, anti‐angiogenesis, and immune modulation.^26^ However, results from RCTs do not support a causal role between supplementary vitamin D (from 800 IU/d to 1000 IU/d with or without calcium supplementation for 1‐7 years) and CRC risk (Table S8). Similarly, the eight overlapping MR studies included in this review did not identify a causal association between blood level of vitamin D and CRC risk (Table S6). Therefore, currently, there is no evidence for a clear causal role of vitamin D on CRC risk. It is also possible that the nonsignificant results from RCTs and MR studies are due to the distinct limitations of these two study designs, such as limited follow‐up time, insufficient supplementary dose, and contamination of controls for RCTs and collider bias, limited power, and potential pleiotropy for MR studies.

A statistically significant association between diabetes and CRC risk has been previously identified by an umbrella review published in 2014.^27^ In the current study, among the insulin‐related biomarkers, HOMA‐IR (a method to quantify insulin resistance based on the blood concentration of glucose and insulin) showed suggestive evidence (Class III) for an association with a higher risk of CRC. Similarly, IGF‐1 and fasting glucose had weak evidence for an association with CRC risk. Elevated glucose and insulin levels may increase CRC risk through their pro‐proliferation, pro‐angiogenesis, and apoptosis inhabitation effects against tumor cell.^28^ For example, exposure to high glucose could lead to increased generation of reactive oxygen intermediates and subsequently could induce apoptosis of endothelial cells.^29^ In addition, hyperglycemia could increase the concentration of circulating inflammatory cytokines leading to chronic inflammation, which has been suggested to relate to tumor generation.^30^, ^31^ However, in this review, we did not find evidence of an association between inflammatory markers and CRC risk. The tumor cell growth simulated by high concentrations of insulin through the activation of IGF‐1, and the possible protective effect of the use of metformin (found in a meta‐analysis including 12 cohort studies, seven case‐controls studies, and one RCT)^32^ on CRC development further supports the insulin‐CRC association. In conclusion, preclinical and epidemiological evidence supports an association between insulin‐related biomarkers on CRC risk, but causality is not supported by MR studies. We should note that diabetes shares many risk factors with CRC, which could explain the observed associations from observational studies.

Interestingly, seven different types of pathogenic microorganisms were found to be related to CRC risk, but most of the evidence was classified as weak due to small number of cases. Only HPV showed a suggestive association with CRC risk. HPV is a non‐enveloped double‐stranded DNA virus with more than 170 types. Twelve of these types are considered as causal risk factors for cervical cancer (known as high‐risk HPV types) by IARC Monographs.^33^, ^34^ In addition, HPV 16, HPV 18, and HPV 33 have commonly been found in CRC cases.^35^, ^36^, ^37^ The potential mechanisms of HPV on colorectal carcinogenesis include viral integration in host cells and expression of E6 and E7 oncoproteins; however, evidences of whether HPV infection is truly involved in colorectal carcinogenesis are still not convincing.^38^ Furthermore, this finding should be interpreted with caution, since the HPV‐CRC association was analyzed without stratifying by HPV type. Meanwhile, all the included meta‐analyses synthesized retrospective observational studies; therefore, the observed associations could be due to reverse causality.

Overall, meta‐analyses of observational studies indicated weak associations between non‐genetic biomarkers and CRC risk. In this review, only seven of 65 associations fulfilled the *P*‐value threshold of convincing evidence, and of these three were based on evidence from less than 1000 cases, three did not have a statistically significant *P*‐value for their largest component study and for one there was evidence of small study effect bias and excess significance bias. Despite weak evidence after applying the predefined credibility criteria, we cannot ignore the clinical importance of these associations. Notably, most (85%) of the overlapping studies agreed on the direction of effect estimate and over half (60%) agreed on both the direction and statistical significance.

### 4.2 Mendelian randomization studies

Almost half of the biomarkers identified from MR studies were biomarkers of fatty acid/lipid metabolism. Most of the detected MR studies had insufficient power (<0.8). There were nine MR studies that reported statistically significant results. After retaining the largest MR study for each biomarker and applying the predefined assessment criteria, we found evidence that high blood levels of linoleic acid and oleic acid and low blood levels of arachidonic acid were associated with low CRC risk. Conversely, LDL‐cholesterol, omega‐6 polyunsaturated fatty acids (n‐6 PUFAs), total cholesterol, selenium, vitamin B12, telomere length, and adiponectin were not found to be associated with CRC risk.

n‐3 and n‐6 PUFAs are essential fatty acids and cannot be produced in the human body.^39^ The beneficial effects of high levels of n‐3 PUFAs and low levels of n‐6 PUFAs on CRC risk reduction remain debatable. In this review, a weak protective effect of n‐3 PUFAs on CRC risk was detected from meta‐analyses of prospective observational studies while MR analyses did not show any evidence of causality. Similarly, RCTs did not report any association between supplementation of n‐3 fatty acids (combination of EPA and DHA) and CRC incidence.^40^, ^41^ Arachidonic acid is an n‐6 PUFA, which in this review is suggested to causally increase the risk of CRC. The potential mechanism is that arachidonic acid can regulate CRC development through the inhibition of cyclooxygenase (COX)/lipoxygenase (LOX) and has a competitive relation to DPA in terms of COX enzyme activity.^39^, ^42^ Oleic acid and linoleic acid are two of the main components of olive oil and have been examined as protective biomarkers for CRC risk by MR studies in this review. These findings, along with evidence from a literature review on olive oil intake and a cohort study on Mediterranean diet,^43^, ^44^ support the beneficial effect of oleic acid and linoleic acid on CRC risk. However, the genetic instruments for the two n‐6 PUFAs are similar, which indicate the possibility that arachidonic acid and linoleic acid may share the same pathway to influence CRC risk and represent vertical pleiotropy.

Overall, we found that there was either lack of evidence or that the credibility of evidence varied across the three different study designs. For instance, evidence detected from meta‐analyses of observational studies was not confirmed by MR studies or meta‐analyses of RCTs on supplementary micronutrients (ie, in vitamin D). This may be either due to differences in the study designs (observational study tests the presence of associations while MR study and RCT explore causality) or due to their inherent distinct limitations and biases. Conversely, four “likely noncausal” associations identified from MR studies also were reported as negative results by meta‐analyses of observational studies, that is, selenium, total cholesterol, LDL‐cholesterol, and telomere length.

## 5 STRENGTHS AND LIMITATIONS

This umbrella review presents for the first time, integrated evidence from meta‐analyses of observational studies, MR studies, and RCTs with the aim to improve our understanding of the associations between non‐genetic biomarkers and CRC risk. Each of the included studies have different strength and limitations and, if consistent, could strengthen our confidence in findings.^45^ The umbrella review design has a number of strengths as previously summarized.^46^, ^47^, ^48^, ^49^ It also has several limitations. First, in an umbrella review, only systematic reviews with meta‐analyses and MR studies are included, thus associations with biomarkers that have not been included in meta‐analyses are not evaluated (ie, circulation sex hormone levels).^50^, ^51^ We did not search for pre‐print articles which are not peer reviewed, and we have therefore not included studies of newly detected CRC‐related biomarkers. Given that no meta‐analyses of RCTs on biomarkers were identified, we included meta‐analyses of RCTs on intake of micronutrients as proxies of micronutrient levels measured in blood. Along with the inclusion of MR studies, these might offset the absence of meta‐analyses of RCTs. A note of caution though is the uncertain association between supplementary dose and physiological dose of micronutrients across participants. Second, there might have been heterogeneity of effects based on anatomical site,^52^ gender, body mass index, diabetes mellitus, and other risk factors,^53^ but we did not perform any subgroup analysis. Third, we did not evaluate the quality assessment of the component studies of each meta‐analysis of observational studies (apart from meta‐analyses classified as convincing or highly suggestive) and the eligibility of component studies depended on the authors of each meta‐analysis. Most of the included meta‐analyses estimated the quality of the individual studies by applying the Newcastle‐Ottawa Scale, which has low reliability between independent reviewers.^54^ Fourth, the limitations of the adopted credibility assessment criteria have been described previously and also apply here.^46^, ^47^, ^48^, ^49^ Finally, evidence from meta‐analyses of observational studies could be biased by confounding factors or by reverse causality.

## 6 CONCLUSION

This umbrella review represents a comprehensive summary and evidence triangulation of a wide range of CRC risk‐associated non‐genetic biomarkers. In conclusion, we report and classify the evidence for non‐genetic biomarkers detected from meta‐analyses of observational studies, MR studies, and meta‐analyses of RCTs. Convincing evidence of a clear role of a non‐genetic biomarker in CRC risk has not been detected from meta‐analyses of observational studies. From MR studies, a likely causal increased CRC risk with arachidonic acid and a likely causal decreased risk with linoleic acid and oleic acid were suggested. Conversely, seven biomarkers (LDL‐cholesterol, n‐6 PUFAs, total cholesterol, selenium, vitamin B12, telomere length, and adiponectin) are likely noncausal. Four (LDL‐cholesterol, total cholesterol, selenium, and telomere length) of these seven biomarkers have consistent results (likely noncausal) from MR and meta‐analyses of observational studies.

## CONFLICT OF INTEREST

None declared.

## AUTHOR CONTRIBUTIONS

ET conceived this study. XZ and DG conducted the literature search. XZ and TY conducted literature screening. XZ extracted the data. YH, GM, and DG checked the extracted data. XZ analyzed the data and draft the manuscript. All authors interpret the data, revised the manuscript, and approved the final version. ET is guarantor.

## ETHICAL APPROVAL

Not required.

## Supporting information

Table S1‐S8

## Data Availability

No additional data available.
