# Supplementary material for: Non‐genetic biomarkers and colorectal cancer risk: Umbrella review and evidence triangulation
Source: Cancer Med. 2020 May 12;9(13):4823–35. doi: 10.1002/cam4.3051 (PMC7333850; doi:10.1002/cam4.3051)
Supplement: Supplementary file 1 — Table S1‐S8 [file CAM4-9-4823-s001.docx]

**Supplementary tables**

| **Supplementary Table 1 Search strategy** | | | |
| --- | --- | --- | --- |
| **Search strategy for meta-analyses of observational studies and randomised clinical trials** | | | |
|  | MEDLINE |  | EMBASE |
| 1 | ((rectal or rectum or colonic or colon or colorectal or bowel* or sigmoid or intestin*) adj3 (cancer* or carcinoma* or neoplas* or tumor* or tumour* or adenocarcinoma* or adeno?carcinoma* or adenom* or lesion* or CRC )).mp. | 1 | ((rectal or rectum or colonic or colon or colorectal or bowel* or sigmoid or intestin*) adj3 (cancer* or carcinoma* or neoplas* or tumor* or tumour* or adenocarcinoma* or adeno?carcinoma* or adenom* or lesion* or CRC )).mp. |
| 2 | exp Colorectal Neoplasms/ | 2 | exp colon tumor/ |
| 3 | 1 or 2 | 3 | exp rectum tumor/ |
| 4 | meta analy$.tw. | 4 | exp colon carcinoma/ |
| 5 | metaanaly$.tw. | 5 | exp colorectal carcinoma/ |
| 6 | (systematic adj (review$1 or overview$1)).tw. | 6 | exp rectum carcinoma/ |
| 7 | Meta-Analysis/ | 7 | exp colon cancer/ |
| 8 | exp "REVIEW LITERATURE AS TOPIC"/ | 8 | exp rectum cancer/ |
| 9 | exp Meta-Analysis as Topic/ | 9 | 1 or 2 or 3 or 4 or 5 or 6 or 7 or 8 |
| 10 | 4 or 5 or 6 or 7 or 8 or 9 | 10 | (meta adj analy$).tw. |
| 11 | 3 and 10 | 11 | metaanalys$.tw. |
| 12 | limit 11 to yr="2010 - 2019" | 12 | (systematic adj (review$1 or overview$1)).tw. |
|  |  | 13 | meta analysis/ |
|  |  | 14 | exp "systematic review"/ |
|  |  | 15 | exp "meta analysis (topic)"/ |
|  |  | 16 | 10 or 11 or 12 or 13 or 14 or 15 |
|  |  | 17 | 9 and 16 |
|  |  | 18 | limit 17 to yr="2010 - 2019" |
| **Search strategy for Mendelian randomisation studies** | | | |
| ((rectal or rectum or colonic or colon or colorectal or bowel* or sigmoid or intestin*) and (cancer or carcinoma* or neoplas* or tumor or tumour or adenocarcinoma* or adeno?carcinoma* or adenom* or lesion* or CRC) and (Mendelian and randomi*)).mp. | | | |

| **Supplementary Table 2 Characteristics and main findings of meta-analyses of observational studies reporting unique non-genetic biomarkers and CRC risk (HKSJ estimator)** | | | | | | | | | | | | | |
| --- | --- | --- | --- | --- | --- | --- | --- | --- | --- | --- | --- | --- | --- |
| First author, Year (Citation) | Biomarker | Comparison | No of studies | No of cases | Total No | Metric | Effect size (95% CI) | P value | I^2^ | ^1^P_Egger_ | 95% Prediction interval | ^2^P_sig_ | Evidence |
| **Fatty acid/Lipid metabolism biomarkers** | | | | | | | | | | | | | |
| Yang B,2014 | LC n-3 PUFA | Highest vs. lowest categories | 3 | 421 | 1,826 | RR | 0.58(0.40, 0.84) | 0.0042 | 0.21% | 0.78 | 0.578(0.398, 0.841) | NA | Weak |
| Yang B,2014 | Biospecimen EPA 20:5 (n-3) | Highest vs lowest categories | 4 | 421 | 1,826 | RR | 0.64(0.42, 0.96) | 0.0314 | 9.24% | 0.50 | 0.637(0.422, 0.961) | NA | Weak |
| Yang B,2014 | Biospecimen DHA 22:6 (n-3) | Highest vs. lowest categories | 4 | 421 | 1,826 | RR | 0.51(0.28, 0.93) | 0.0266 | 46.59% | 0.19 | 0.513(0.284, 0.925) | NA | Weak |
| Yang B,2014 | Biospecimen DPA 22:5 (n-3) | Highest vs. lowest categories | 3 | 243 | 1,366 | RR | 0.56(0.29, 1.07) | 0.0788 | 37.89% | 0.46 | 0.561(0.295, 1.069) | NA | No |
| Yang B,2014 | Blood EPA 20:5 (n-3) | Mean (% total fatty acids) | 5 | 195 | 384 | SMD | -0.98(-2.87, 0.91) | 0.308 | 98.87% | 0.01 | -0.983(-2.873, 0.907) | NA | No |
| Yang B,2014 | Blood DHA 22:6 (n-3) | Mean (% total fatty acids) | 5 | 195 | 384 | SMD | -0.07(-0.31, 0.17) | 0.5718 | 35.10% | 0.41 | -0.068(-0.306, 0.169) | NA | No |
| Yang B,2014 | Blood DPA 22:5 (n-3) | Mean (% total fatty acids) | 5 | 195 | 384 | SMD | 0.43(-0.27, 1.12) | 0.2292 | 92.11% | 0.00 | 0.427(-0.269, 1.122) | NA | No |
| Yang B,2014 | Adipose EPA 20:5 (n-3) | Mean (% total fatty acids) | 2 | 93 | 204 | SMD | 0.07(-0.21, 0.36) | 0.6083 | 3.39% | / | 0.074(-0.208, 0.356) | 0.75 | No |
| Yang B,2014 | Adipose DHA 22:6 (n-3) | Mean (% total fatty acids) | 2 | 93 | 204 | SMD | -0.02(-0.37, 0.32) | 0.8999 | 35.64% | / | -0.022(-0.368, 0.324) | NA | No |
| Yang B,2014 | Adipose DPA 22:5 (n-3) | Mean (% total fatty acids) | 2 | 93 | 204 | SMD | 0.12(-0.16, 0.39) | 0.4082 | 0.07% | / | 0.117(-0.160, 0.395) | 0.69 | No |
| Yao X, 2015 | HDL-cholesterol | Highest vs. lowest categories | 12 | 2,542 | 136,698 | RR | 0.83(0.67, 1.04) | 0.1043 | 53.08% | 0.39 | 0.83(0.67, 1.04) | 0.01 | No |
| Yao X, 2015 | LDL-cholesterol | Highest vs. lowest categories | 5 | 1,626 | 9,175 | RR | 1.04 (0.57, 1.92) | 0.8908 | 85.67% | 0.16 | 1.04(0.57, 1.92) | 0.0002 | No |
| Yao X, 2015 | Total cholesterol | Highest vs. lowest categories | 28 | 10,892 | 7,725,310 | RR | 1.11(0.98, 1.27) | 0.1043 | 76.10% | 0.60 | 1.12(0.98, 1.27) | NA | No |
| Yao X, 2015 | Triglyceride | Highest vs. lowest categories | 19 | 8,127 | 2,252,217 | RR | 1.17 (0.99, 1.37) | 0.0598 | 47.77% | 0.22 | 1.17(0.99, 1.37) | NA | No |
| **Infectious agents** | | | | | | | | | | | | | |
| Ibragimova MK,2018[^25^](#_ENREF_25) | HPV | CRC tissue vs Cancer-free tissue | 17 | 1,722 | 2,468 | RR | 3.52(1.77, 7.00) | 0.0003 | 75.66% | 0.6453 | 3.52(1.77, 7.00) | NA | Suggestive |
| Bai B,2016 | Human cytomegalovirus infection | Tumour tissue vs normal tissue | 4 | 480 | 960 | OR | 6.47(4.23, 9.89) | 6.7839E-18 | 13.74% | 0.70 | 6.47(4.23, 9.89) | 0.99 | Weak |
| Boleij A,2011 | Streptococcus bovis | CRC in S.bovis type 1 & type 2 | 6 | 189 | 340 | OR | 9.44(4.43, 20.11) | 5.95E-09 | 18.72% | 0.57 | 9.44(4.43, 20.11) | NA | Weak |
| Liu C,2016 | H.pylori | CRC vs non-CRC | 20 | 3,228 | 4,377 | OR | 1.38(1.01, 1.89) | 4.00E-02 | 82.00% | 0.90 | 1.38(1.02, 1.89) | 0.27 | Weak |
| Repass J,2018 | F. nucleatum | CRC tissue vs adjacent normal tissue | 2 | 139 | 278 | r | 0.40(0.16, 0.63) | 0.00100 | 40.05% | / | 0.38(0.16, 0.56) | NA | Weak |
| Liu H,2016 | Enterobacteriaceae | Healthy controls (logarithmic number of bacteria per gram stool) | 2 | 37 | 76 | SMD | 2.62(0.62, 4.61) | 0.0101 | 87.87% | / | 2.62(0.62, 4.61) | NA | Weak |
| Liu H,2016 | Bifidobacterium | Healthy controls (logarithmic number of bacteria per gram stool) | 4 | 127 | 315 | SMD | -3.24(-6.32, -0.16) | 0.039 | 98.08% | 0.07 | -3.24(-6.32, -0.16) | 0.00 | Weak |
| Liu H,2016 | Faecalibacterium prausnitzii | Healthy controls (logarithmic number of bacteria per gram stool) | 3 | 86 | 233 | SMD | -0.31(-0.69, 0.06) | 0.1 | 25.76% | 0.60 | -0.31(-0.69, 0.06) | 0.80 | No |
| Liu H,2016 | Total bacteria | Healthy controls (logarithmic number of bacteria per gram stool) | 3 | 86 | 233 | SMD | 0.29(-0.74, 1.31) | 0.5825 | 87.31% | 0.07 | 0.29(-0.74, 1.31) | 0.28 | No |
| Krishnan S,2014 | Streptococcus bovis in faeces | CRC vs non-CRC | 3 | 148 | 414 | OR | 3.10(0.68, 14.20) | 0.1456 | 56.37% | 0.23 | 3.10(0.68, 14.20) | NA | No |
| Liu H,2016 | Lactobacillus | Healthy controls (logarithmic number of bacteria per gram stool) | 4 | 127 | 315 | SMD | -1.85(-5.33, 1.64) | 0.2993 | 99.15% | 0.19 | -1.85(-5.33, 1.64) | 0.00 | No |
| Liu H,2016 | Bacteroides-Prevotella group | Healthy controls (logarithmic number of bacteria per gram stool) | 3 | 97 | 255 | SMD | -0.70(-2.33, 0.94) | 0.403 | 95.30% | 0.83 | -0.70(-2.33, 0.94) | NA | No |
| Liu H,2016 | Escherichia coli | Healthy controls (logarithmic number of bacteria per gram stool) | 2 | 90 | 239 | SMD | 1.38(-1.34, 4.10) | 0.3195 | 97.96% | / | 1.38(-1.34, 4.10) | 0.00 | No |
| **Inflammatory Markers** | | | | | | | | | | | | | |
| Zhou B,2014 | CRP | 1-unit change in ln (mg/l) | 18 | 4,779 | 152,942 | RR | 1.14(1.04, 1.25) | 0.0059 | 72.80% | 0.05 | 1.14(1.04, 1.25) | 0.03 | Weak |
| Zhou B,2014 | IL-6 | 1-unit change in ln (mg/l) | 6 | 1,125 | 9,909 | RR | 1.09(0.85, 1.39) | 0.4959 | 47.99% | 0.19 | 1.09(0.85, 1.39) | 0.595 | No |
| **Insulin related biomarkers** | | | | | | | | | | | | | |
| Xu J,2016 | Fasting glucose | Highest vs lowest categories (mmol/L) | 26 | 20,390 | 5,105,567 | RR | 1.27(1.11, 1.45) | 0.0006 | 66.17% | 0.0106 | 1.27(1.11, 1.45) | NA | Highly suggestive |
| Xu J,2016 | HOMA-IR | Highest vs lowest categories (fasting glucose (mmol/L) × fasting insulin (mIU/L) / 22.5) | 9 | 2,956 | 18,358 | RR | 1.56(1.22, 1.98) | 0.0003 | 38.91% | 0.18 | 1.56(1.22, 1.99) | NA | Suggestive |
| Xu J,2016 | Fasting insulin | Highest vs lowest categories (mIU/L) | 11 | 3,191 | 26,301 | OR | 1.40 (1.12, 1.74) | 0.0031 | 24.65% | 0.86 | 1.40(1.12, 1.74) | NA | Weak |
| Chi F,2013 | IGF 1 | Highest with lowest categories | 17 | 3,807 | 11,613 | OR | 1.31(1.05, 1.63) | 0.0187 | 48.42% | 0.28 | 1.31(1.05, 1.63) | NA | Weak |
| Chi F,2013 | IGF 2 | Highest with lowest categories | 6 | 783 | 4,007 | OR | 1.52(0.99, 2.34) | 0.0549 | 42.30% | 0.62 | 1.52 (0.99, 2.34) | NA | No |
| Chi F,2013 | IGFBP 1 | Highest with lowest categories | 7 | 2,154 | 6,439 | OR | 0.81(0.61, 1.09) | 0.1585 | 43.13% | 0.42 | 0.81(0.61, 1.09) | 0.2816 | No |
| Chi F,2013 | IGFBP 2 | Highest with lowest categories | 3 | 1,348 | 2,962 | OR | 0.76(0.41, 1.43) | 0.4016 | 68.77% | 0.02 | 0.77(0.41, 1.43) | NA | No |
| Chi F,2013 | IGFBP 3 | Highest with lowest categories | 16 | 3,755 | 11,509 | OR | 0.88 (0.70, 1.10) | 0.268 | 45.74% | 0.31 | 0.88(0.71, 1.10) | 0.5968 | No |
| Xu J,2016 | HbA1c | Highest vs lowest categories (%) | 8 | 2,137 | 45,569 | RR | 1.25(0.93, 1.67) | 0.1414 | 53.83% | 0.30 | 1.25(0.99, 1.67) | NA | No |
| Xu J,2016 | C-peptide | Highest vs lowest categories(ng/ml) | 11 | 3,211 | 13,888 | RR | 1.35(0.97, 1.89) | 0.0788 | 70.63% | 0.73 | 1.35(0.97, 1.89) | 0.776 | No |
| **Micronutrients** | | | | | | | | | | | | | |
| Ma YL,2011 | 25-hydroxyvitamin D | Highest with lowest categories | 10 | 3,142 | 7,840 | RR | 0.67(0.54, 0.83) | 0.0002 | 20.49% | 0.79 | 0.670 (0.541, 0.830) | NA | Suggestive |
| Ben S,2018 | Vitamin B2 | Highest with lowest categories | 2 | 1,593 | 32,962 | RR | 0.74(0.57, 0.95) | 0.02 | 9.32% | / | 0.74(0.58, 0.95) | NA | Weak |
| Larsson SC, 2010 | Vitamin B6 | Highest vs lowest categories (pmol/) | 4 | 883 | 2,207 | RR | 0.53(0.38, 0.71) | 0.0012 | 0.89% | 0.97 | 0.52(0.38, 0.72) | NA | Weak |
| Shiao SPK,2018 | Vitamin B12 | Mean (pmol/L) | 8 | 3,296 | 8,290 | SMD | -0.07(-0.14, -0.004) | 0.0384 | 54.91% | 0.03 | -0.07(-0.19, 0.05) | 0.47 | Weak |
| Zhang D, 2015 | Folate | CRC vs healthy controls | 12 | 1,159 | 2,982 | SMD | -1.29(-2.29, -0.30) | 0.0105 | 99.57% | 0.0442 | -1.29(-3.09, 0.51) | NA | Weak |
| Dong YH,2017 | Vitamin E | Mean (µmol/L) | 9 | 310 | 5,927 | SMD | -0.79(-1.63, 0.05) | 0.065 | 96.61% | 0.01 | -0.79(-1.63, 0.05) | 0.3192 | No |
| Lee JE,2011 | 1,25-dihydroxyvitamin D | Highest with lowest categories | 4 | 625 | 1,801 | OR | 1.01(0.66, 1.59) | 0.9302 | 42.59% | 0.64 | 1.020 (0.656, 1.585) | NA | No |
| Vinceti M,2018 | Selenium | Highest vs. lowest category | 8 | 2,627 | 712,746 | OR | 0.86(0.62, 1.18) | 0.3509 | 42.92% | 0.7895 | 0.859(0.624, 1.182) | NA | No |
| Gumulec J,2014 | Serum Zinc | Largest variation among serum levels | 5 | 313 | 529 | SMD | 0.05(-3.47, 3.56) | 0.9788 | 99.40% | 0.7342 | 0.05(-3.47, 3.56) | 0.00 | No |
| Gumulec J,2014 | Tissue Zinc | Largest variation among tissue levels | 10 | 234 | 398 | SMD | -0.76(-3.08, 1.55) | 0.5187 | 98.75% | 0.00 | -0.76(-3.08, 1.55) | 0.1503 | No |
| **Other Biomarkers** | | | | | | | | | | | | | |
| Zhang BL,2015 | Blood group A | A vs non-A | 8 | 6,931 | 3,214,941 | OR | 1.01(0.90, 1.14) | 0.8533 | 75.87% | 0.12 | 1.01(0.90, 1.14) | 0.09 | No |
| Zhang BL,2015 | Blood group AB | AB vs non-AB | 8 | 6,931 | 3,191,289 | OR | 0.94(0.73, 1.21) | 0.6416 | 61.18% | 0.44 | 0.94(0.73, 1.21) | NA | No |
| Zhang BL,2015 | Blood group B | B vs non-B | 8 | 6,931 | 3,193,532 | OR | 1.00(0.91, 1.10) | 0.9963 | 18.12% | 0.41 | 1.00(0.91, 1.10) | NA | No |
| Zhang BL,2015 | Blood group O | O vs non-O | 8 | 6,931 | 3,219,151 | OR | 0.91(0.84, 1.00) | 0.0445 | 54.58% | 0.23 | 0.91(0.84, 1.00) | NA | Weak |
| Naing C,2017 | Telomere Length | Shortest Q4 vs longest Q2 | 8 | 951 | 2,569 | OR | 1.02(0.75, 1.40) | 0.8842 | 42.82% | 0.34 | 1.02(0.75, 1.40) | NA | No |
| Jiang R,2016 | Enterolactone | Per doubling (nmol/l) | 3 | 762 | 2,408 | RR | 1.14(0.89, 1.47) | 0.2924 | 66.71% | 0.06 | 1.14(0.89, 1.47) | NA | No |
| **Protein & amino acids** | | | | | | | | | | | | | |
| Lu S,2017 | Total Adiponectin | Highest vs lowest categories | 8 | 3,420 | 8,937 | RR | 0.78(0.65, 0.95) | 0.0143 | 39.81% | 0.38 | 0.79(0.65, 0.95) | 0.578 | Weak |
| Yang G,2016 | Resistin | Mean (ng/mL) | 11 | 965 | 2,290 | SMD | 0.65(0.19, 1.11) | 0.0059 | 94.79% | 0.94 | 0.65(0.19, 1.11) | NA | Weak |
| Shiao SPK,2018 | Homocysteine | Mean (mmol/L) | 8 | 4,047 | 9,604 | SMD | 0.13(0.04, 0.21) | 0.003 | 68.35% | 0.69 | 0.13(-0.03, 0.28) | NA | Weak |
| Yu DD,2018 | Angiogenin | Mean (ng/ml) | 2 | 188 | 240 | SMD | 1.53(0.52, 2.54) | 0.003 | 87.41% | / | 1.53(0.52, 2.54) | NA | Weak |
| Xing XJ,2014 | MMP7 | Mean (% total fatty acids) | 10 | 625 | 1,020 | SMD | 2.31(0.91, 3.71) | 0.0013 | 98.80% | 0.00 | 2.31(-0.24, 4.86) | 0.4128 | Weak |
| Li XX,2014 | TLR-4 protein | CRC vs healthy controls | 3 | 168 | 283 | OR | 4.75(1.16, 19.45) | 0.0304 | 77.55% | 0.01 | 4.75(1.16, 19.46) | 0.0181 | Weak |
| Sun SJ,2016 | HER‑2 expression | CRC vs healthy controls | 13 | 932 | 1,453 | OR | 11.82(5.36, 26.08) | 9.33E-10 | 63.02% | 0.00 | 11.82(5.36, 26.08) | 0.103 | Weak |
| Feng Z,2015 | Ferritin | Mean (ng/ml) | 7 | 277 | 927 | SMD | -1.57(-3.06, -0.08) | 0.0388 | 98.50% | 0.00 | -1.57(-3.06, -0.08) | NA | Weak |
| Ouyang Z,2017 | CD26 | Tumour cell vs normal cell | 9 | 952 | 1,809 | SMD | -0.33(-4.35, 3.70) | 0.8737 | 99.84% | 0.45 | -0.33(-7.62, 6.97) | 0.0098 | No |
| Gialamas SP,2013 | Leptin | Mean | 23 | 3,508 | 7,478 | SMD | 0.20(-0.32, 0.72) | 0.4551 | 99.03% | 0.58 | 0.20(-0.75, 1.14) | 0.000000 | No |
| CRC: colorectal cancer, CI: confidence interval, HKSJ: Hartung-Knapp-Sidik-Jonkman, ^1^PEgger: The P value for small study effect test, ^2^Psig: The P value for excess significance test, RR: risk ratio, SMD: standard mean difference, OR: odds ratio, r: standardized correlation coefficient, LC n-3 PUFA: long chain n-3 polyunsaturated fatty acid, EPA: Eicosapentaenoic acid, DHA: Docosahexaenoic acid, DPA: Docosapentaenoic acid, HDL: high-density lipoprotein cholesterol, LDL: low-density lipoprotein cholesterol, HPV: Human papillomavirus, H.pylori: Helicobacter pylori, CRP: C-reactive protein, IL-6: Interleukin 6, HOMA-IR: homeostatic model assessment-insulin resistance, IGF 1/2: Insulin-like growth factor 1/2, IGFBP 1/2/3: Insulin-like growth factor-binding protein 1/2/3, HbA1c: glycated hemoglobin, MMP7: matrix metalloproteinase-7, CD26: dipeptidyl peptidase IV. | | | | | | | | | | | | | |

| **Supplementary Table 3 Characteristics and main findings of 42 non-overlapped Mendelian randomisation analyses** | | | | | | | | | | | | | | | |
| --- | --- | --- | --- | --- | --- | --- | --- | --- | --- | --- | --- | --- | --- | --- | --- |
| First author, Year (Citation) | Biomarker (Unit) | Power to detect given effect estimates | Exposure | | | Outcome | | | Study design | Main method | Main estimate | P value | Sensitivity analyses | Consistent evidence of a causal effect | Evidence |
|  |  |  | Sample size | Population | Variance (R^2^) explained by GI (%) | Sample size | Metric | Population |  |  |  |  |  |  |  |
| **Micronutrients** | | | | | | | | | | | | | | | |
| Cornish AJ, 2019 | Blood selenium | 1.000\|OR≤0.75 or OR≥1.33 | 2,603 participants | Queensland | 2 | 26,397 cases and 41,481 controls | OR per SD | European | Two-sample | Wald ratio | 0.85 (0.75, 0.96) | 0.008** | Weighted median, mode-based estimates, MR-Egger, leave-one-out analysis | No | Likely non-causal |
| Cornish AJ, 2019 | Blood zinc | 1.000\|OR≤0.75 or OR≥1.33 | 2,603 participants | Queensland | 4.6 | 26,397 cases and 41,481 controls | OR per SD | European | Two-sample | Maximum likelihood | 0.94 (0.86, 1.03) | 0.179** | Weighted median, mode-based estimates, MR-Egger, leave-one-out analysis | No | Unknown |
| Cornish AJ, 2019 | Circulating 25-hydroxyvitamin D | 1.000\|OR≤0.75 or OR≥1.33 | 79,366 participants | European | 2.6 | 26,397 cases and 41,481 controls | OR per SD | European | Two-sample | Maximum likelihood | 0.99 (0.90, 1.09) | 0.895** | Weighted median, mode-based estimates, MR-Egger, leave-one-out analysis | No | Unknown |
| Cornish AJ, 2019 | Circulating carotenoids | 1.000\|OR≤0.75 or OR≥1.33 | 1,190 participants | Italian | 2.8 | 26,397 cases and 41,481 controls | OR per SD | European | Two-sample | Wald ratio | 1.04 (0.94, 1.15) | 0.451** | Weighted median, mode-based estimates, MR-Egger, leave-one-out analysis | No | Unknown |
| Cornish AJ, 2019 | Iron status | 0.981\|OR≤0.75 or OR≥1.33 | 48,972 participants | European | 1.2 | 26,397 cases and 41,481 controls | OR per SD | European | Two-sample | Maximum likelihood | 1.17 (1.00, 1.36) | 0.049** | Weighted median, mode-based estimates, MR-Egger, leave-one-out analysis | No | Unknown |
| Cornish AJ, 2019 | Serum calcium | 1.000\|OR≤0.75 or OR≥1.33 | 39,400 participants | European | 2.6 | 26,397 cases and 41,481 controls | OR per SD | European | Two-sample | Maximum likelihood | 0.93 (0.83, 1.05) | 0.264** | Weighted median, mode-based estimates, MR-Egger, leave-one-out analysis | No | Unknown |
| Cornish AJ, 2019 | Serum vitamin A (retinol) | 0.879\|OR≤0.75 or OR≥1.33 | 5,006 participants | Finland, US | 0.7 | 26,397 cases and 41,481 controls | OR per SD | European | Two-sample | Maximum likelihood | 1.07 (0.78, 1.47) | 0.663** | Weighted median, mode-based estimates, MR-Egger, leave-one-out analysis | No | Unknown |
| Cornish AJ, 2019 | Serum vitamin B12 | 1.000\|OR≤0.75 or OR≥1.33 | 45,576 & 37,341 participants | Iceland, Denmark | 4.7 | 26,397 cases and 41,481 controls | OR per SD | European | Two-sample | Maximum likelihood | 1.21 (1.04, 1.42) | 0.016** | Weighted median, mode-based estimates, MR-Egger, leave-one-out analysis | No | Likely non-causal |
| Cornish AJ, 2019 | Serum vitamin B6 | 0.994\|OR≤0.75 or OR≥1.33 | 2,930 participants |  | 1.4 | 26,397 cases and 41,481 controls | OR per SD | European | Two-sample | Wald ratio | 1.04 (0.90, 1.20) | 0.592** | Weighted median, mode-based estimates, MR-Egger, leave-one-out analysis | No | Unknown |
| Cornish AJ, 2019 | Serum vitamin E | 0.857\|OR≤0.75 or OR≥1.33 | 5,006 participants | European | 0.7 | 26,397 cases and 41,481 controls | OR per SD | European | Two-sample | Maximum likelihood | 0.94 (0.76, 1.17) | 0.600** | Weighted median, mode-based estimates, MR-Egger, leave-one-out analysis | No | Unknown |
| **Fatty acid/Lipid metabolism biomarkers** | | | | | | | | | | | | | | | |
| May-Wilson S, 2017 | Plasma Arachidic acid (20:0) (GRS) | Limited power (not specified) | 38,000 participants | European | / | 9,254 cases and 18,386 controls | OR per SD | European | Two-sample | Meta-analysis of statistics for each specific fatty acid generated for each CRC cohort was combined under fixed-effects models | 0.92(0.61, 1.39) | 0.700 | Where more than one instrument variant was available: heterogeneity assessment, random-effects inverse-variance weighted MR; assessing impact of pleiotropy by using IVW and MR-Egger methods | No | Unknown |
| May-Wilson S, 2017 | Plasma Palmitic acid (16:0) (GRS) | Limited power (not specified) | 38,000 participants | European | 0.21-0.98 | 9,254 cases and 18,386 controls | OR per SD | European | Two-sample | Meta-analysis of statistics for each specific fatty acid generated for each CRC cohort was combined under fixed-effects models | 0.97(0.78, 1.21) | 0.820 | Where more than one instrument variant was available: heterogeneity assessment, random-effects inverse-variance weighted MR; assessing impact of pleiotropy by using IVW and MR-Egger methods | No | Unknown |
| May-Wilson S, 2017 | Plasma Stearic acid (18:0) (GRS) | Limited power (not specified) | 38,000 participants | European | 0.01-1.39 per SNP | 9,254 cases and 18,386 controls | OR per SD | European | Two-sample | Meta-analysis of statistics for each specific fatty acid generated for each CRC cohort was combined under fixed-effects models | 1.16(1.01, 1.35) | 0.040 | Where more than one instrument variant was available: heterogeneity assessment, random-effects inverse-variance weighted MR; assessing impact of pleiotropy by using IVW and MR-Egger methods | No | Unknown |
| Liyanage UE, 2019 | Plasma DHA 22:6n-3 | / | 8,866 participants | European | 0.65 | 4,545 cases and 270,342 controls combined 9,254 cases and 18,386 controls | OR per SD | European | Two-sample | Wald-type ratio estimator (combining estimate with those from May-Wilson et al 2017) | 1.07(0.84, 1.36) | 0.583* | IVW estimate without combining with data from other study (May-Wilson et al 2017) | No | Unknown |
| Liyanage UE, 2019 | Plasma EPA 20:5n-3 | / | 8,866 participants | European | 2.05 | 4,545 cases and 270,342 controls combined 9,254 cases and 18,386 controls | OR per SD | European | Two-sample | IVW (combining estimate with those from May-Wilson et al 2017) | 1.06(0.91, 1.22) | 0.455* | IVW estimate without combining with data from other study (May-Wilson et al 2017) | No | Unknown |
| Liyanage UE, 2019 | Plasma DPA 20:5n-3 | / | 8,866 participants | European | 11.12 | 4,545 cases and 270,342 controls combined 9,254 cases and 18,386 controls | OR per SD | European | Two-sample | IVW (combining estimate with those from May-Wilson et al 2017) | 1.10(1.01, 1.19) | 0.034* | IVW estimate without combining with data from other study (May-Wilson et al 2017) | No | Unknown |
| Liyanage UE, 2019 | Plasma AA 20:4n-6 | / | 8,631 participants | White adults | 33.07 | 4,545 cases and 270,342 controls combined 9,254 cases and 18,386 controls | OR per SD | European | Two-sample | IVW (combining estimate with those from May-Wilson et al 2017) | 1.05(1.03, 1.07) | 2.00E-05* | IVW estimate without combining with data from other study (May-Wilson et al 2017) | No | Evidence of causality |
| May-Wilson S, 2017 | Plasma DGLA (20:3n-6) (GRS) | Limited power (not specified) | 38,000 participants | European | 2-11.1 per SNP | 9,254 cases and 18,386 controls | OR per SD | European | Two-sample | Meta-analysis of statistics for each specific fatty acid generated for each CRC cohort was combined under fixed-effects models | 0.91(0.83, 1.00) | 0.060 | Where more than one instrument variant was available: heterogeneity assessment, random-effects inverse-variance weighted MR; assessing impact of pleiotropy by using IVW and MR-Egger methods | No | Unknown |
| Liyanage UE, 2019 | Plasma LA 18:2n-6 | / | 8,631 participants | White adults | 8.3 | 4,545 cases and 270,342 controls combined 9,254 cases and 18,386 controls | OR per SD | European | Two-sample | IVW (combining estimate with those from May-Wilson et al 2017) | 0.95(0.93, 0.97) | 9.60E-05* | IVW estimate without combining with other study | No | Evidence of causality |
| May-Wilson S, 2017 | Plasma Oleic acid (18:1n-9) (GRS) | Limited power (not specified) | 38,000 participants | European | 0.32-2.14 per SNP | 9,254 cases and 18,386 controls | OR per SD | European | Two-sample | Meta-analysis of statistics for each specific fatty acid generated for each CRC cohort was combined under fixed-effects models | 0.77(0.65, 0.92) | 0.004 | Where more than one instrument variant was available: heterogeneity assessment, random-effects IVW MR; assessing impact of pleiotropy by using IVW and MR-Egger methods | Yes | Evidence of causality |
| May-Wilson S, 2017 | Plasma Palmitoleic acid (16:1n-7) (GRS) | Limited power (not specified) | 38,000 participants | European | 0.01-1.57 per SNP | 9,254 cases and 18,386 controls | OR per SD | European | Two-sample | Meta-analysis of statistics for each specific fatty acid generated for each CRC cohort was combined under fixed-effects models | 0.36(0.15, 0.84) | 0.018 | Where more than one instrument variant was available: heterogeneity assessment, random-effects IVW MR; assessing impact of pleiotropy by using IVW and MR-Egger methods | No | Unknown |
| Liyanage UE, 2019 | Plasma ALA 18:3n-3 | / | 8,866 participants | European | 1.03 | 4,545 cases and 270,342 controls combined 9,254 cases and 18,386 controls | OR per SD | European | Two-sample | Wald-type ratio estimator (combining estimate with those from May-Wilson et al 2017) | 0.89(0.78, 1.02) | 0.098* | IVW estimate without combining with other study | No | Unknown |
| Cornish AJ, 2019 | HDL | 1.000\|OR≤0.75 or OR≥1.33 | 188,577 participants | European | 6.1 | 26,397 cases and 41,481 controls | OR per SD | European | Two-sample | Maximum likelihood | 1.03 (0.92, 1.14) | 0.620** | Weighted median, mode-based estimates, MR-Egger, leave-one-out analysis | No | Unknown |
| Cornish AJ, 2019 | LDL | 1.000\|OR≤0.75 or OR≥1.33 | 188,577 participants | European | 7.9 | 26,397 cases and 41,481 controls | OR per SD | European | Two-sample | Maximum likelihood | 1.14 (1.04, 1.25) | 0.006** | Weighted median, mode-based estimates, MR-Egger, leave-one-out analysis | No | Likely non-causal |
| Cornish AJ, 2019 | Mono-unsaturated fatty acids | 0.493\|OR≤0.75 or OR≥1.33 | 24,925 participants | European | 0.3 | 26,397 cases and 41,481 controls | OR per SD | European | Two-sample | Wald ratio | 1.07 (0.78, 1.46) | 0.672** | Weighted median, mode-based estimates, MR-Egger, leave-one-out analysis | No | Unknown |
| Cornish AJ, 2019 | Omega-6 polyunsaturated fatty acids | 1.000\|OR≤0.75 or OR≥1.33 | 24,925 participants | European | 2.4 | 26,397 cases and 41,481 controls | OR per SD | European | Two-sample | Maximum likelihood | 1.15 (0.98, 1.36) | 0.095** | Weighted median, mode-based estimates, MR-Egger, leave-one-out analysis | No | Likely non-causal |
| Cornish AJ, 2019 | Total cholesterol | 1.000\|OR≤0.75 or OR≥1.33 | 24,925 participants | European | 9.5 | 26,397 cases and 41,481 controls | OR per SD | European | Two-sample | Maximum likelihood | 1.09 (1.01, 1.18) | 0.025** | Weighted median, mode-based estimates, MR-Egger, leave-one-out analysis | No | Likely non-causal |
| Cornish AJ, 2019 | Total triglycerides | 1.000\|OR≤0.75 or OR≥1.33 | 188,577 participants | European | 6.1 | 26,397 cases and 41,481 controls | OR per SD | European | Two-sample | Maximum likelihood | 0.93 (0.84, 1.04) | 0.192** | Weighted median, mode-based estimates, MR-Egger, leave-one-out analysis | No | Unknown |
| **Inflammatory Markers** | | | | | | | | | | | | | | | |
| Wang X, 2018 | C-reactive protein (19 SNPs) | 0.825\|OR≥1.12 | 66,185 & 40,473 participants | European | 5 | 30,480 cases and 22,844 controls | OR per SD | European | Two-sample | IVW | 1.04(0.97, 1.12) | 0.256 | Subgroup analyses stratified by cancer sites and stages, sex, BMI, smoking, NSAID use, aspirin use, history of endoscopy, family history of CRC; Egger regression | No | Unknown |
| Cornish AJ, 2019 | Plasma IL-6 receptor subunit alpha | 1.000\|OR≤0.75 or OR≥1.33 | 3,301 participants | European | 60.4 | 26,397 cases and 41,481 controls | OR per SD | European | Two-sample | Wald ratio | 0.98 (0.96, 1.00) | 0.035** | Weighted median, mode-based estimates, MR-Egger, leave-one-out analysis | No | Unknown |
| **Insulin related markers** | | | | | | | | | | | | | | | |
| Cornish AJ, 2019 | Fasting glucose | 1.000\|OR≤0.75 or OR≥1.33 | 96,496 participants | European | 3.6 | 26,397 cases and 41,481 controls | OR per SD | European | Two-sample | Maximum likelihood | 1.04 (0.92, 1.18) | 0.519** | Weighted median, mode-based estimates, MR-Egger, leave-one-out analysis | No | Unknown |
| Cornish AJ, 2019 | Fasting proinsulin | 1.000\|OR≤0.75 or OR≥1.33 | 46,186 participants | European | 6.1 | 26,397 cases and 41,481 controls | OR per SD | European | Two-sample | Maximum likelihood | 0.97 (0.90, 1.03) | 0.310** | Weighted median, mode-based estimates, MR-Egger, leave-one-out analysis | No | Unknown |
| Cornish AJ, 2019 | HbA1C levels | 0.999\|OR≤0.75 or OR≥1.33 | 46,368 participants | European | 1.8 | 26,397 cases and 41,481 controls | OR per SD | European | Two-sample | Maximum likelihood | 1.02 (0.85, 1.22) | 0.866** | Weighted median, mode-based estimates, MR-Egger, leave-one-out analysis | No | Unknown |
| Cornish AJ, 2019 | Plasma IGF−I | 0.995\|OR≤0.75 or OR≥1.33 | 3301 participants | European | 1.4 | 26,397 cases and 41,481 controls | OR per SD | European | Two-sample | Wald ratio | 0.88 (0.76, 1.01) | 0.064** | Weighted median, mode-based estimates, MR-Egger, leave-one-out analysis | No | Unknown |
| **Other Biomarkers** | | | | | | | | | | | | | | | |
| Haycock PC, 2017 | Telomere length | 1 | 9,190 participants | European | / | 14,537 cases and 16,922 controls | OR per SD | European | Two-sample | Maximum likelihood | 1.09(0.91, 1.31) | 0.340 | Heterogeneity analysis, weighted median, MR-Egger | No | Likely non-causal |
| **Protein& amino acid** | | | | | | | | | | | | | | | |
| Au Yeung SL, 2019 | GDF-15 | 0.8\|OR≥1.11 (R2=0.15) or OR≥1.10 (R2=0.21) | 5,440 participants | European | 15 or 21 | 4,562 cases and 382,756 controls | OR per SD | European | Two-sample | IVW (Fixed effect) | 0.91(0.80, 1.04) | / | Multiplicative random effect IVW; Lead SNP analysis; MR-Egger intercept test; Mendelian randomisation restricted to instruments from the same gene region (PGPEP1 or GDF15) | No | Unknown |
| Nimptsch K, 2017 | Adiponectin (Incorporating the ADIPOQ allele score and plasma adiponectin concentrations) | 0.8\|OR≤0.76 | 2,880 participants | European and US | / | 1,253 cases and 1,627 controls | OR per score unit | European and US | One-sample | Conditional logistic regression | 0.73(0.40, 1.34) | / | Restriction to Caucasians; Effect estimate in different study (HPFS and NHS) | No | Likely non-causal |
| Cornish AJ, 2019 | Blood carnitine | 1.000\|OR≤0.75 or OR≥1.33 | 7,824 participants | European | 13.9 | 26,397 cases and 41,481 controls | OR per SD | European | Two-sample | Maximum likelihood | 0.99 (0.92, 1.06) | 0.682** | Weighted median, mode-based estimates, MR-Egger, leave-one-out analysis | No | Unknown |
| Cornish AJ, 2019 | Blood methionine | 0.676\|OR≤0.75 or OR≥1.33 | 7,824 participants | European | 0.4 | 26,397 cases and 41,481 controls | OR per SD | European | Two-sample | Wald ratio | 0.92 (0.70, 1.19) | 0.505** | Weighted median, mode-based estimates, MR-Egger, leave-one-out analysis | No | Unknown |
| Cornish AJ, 2019 | Circulating adiponectin | 1.000\|OR≤0.75 or OR≥1.33 | 39,883 participants | European | 1.8 | 26,397 cases and 41,481 controls | OR per SD | European | Two-sample | Maximum likelihood | 0.93 (0.81, 1.07) | 0.309** | Weighted median, mode-based estimates, MR-Egger, leave-one-out analysis | No | Unknown |
| Cornish AJ, 2019 | Circulating fetuin-A | 1.000\|OR≤0.75 or OR≥1.33 | 9,055 & 2,119 participants | European, African American | 14.3 | 26,397 cases and 41,481 controls | OR per SD | European | Two-sample | Wald ratio | 0.98 (0.94, 1.02) | 0.370** | Weighted median, mode-based estimates, MR-Egger, leave-one-out analysis | No | Unknown |
| Cornish AJ, 2019 | Serum immunoglobulin E | 0.997\|OR≤0.75 or OR≥1.33 | 6,819 participants | European | 1.6 | 26,397 cases and 41,481 controls | OR per SD | European | Two-sample | Maximum likelihood | 0.92 (0.82, 1.03) | 0.159** | Weighted median, mode-based estimates, MR-Egger, leave-one-out analysis | No | Unknown |
| *: statistically significant threshold set up at P≤0.0001; **: statistically significant threshold set up at P≤0.0013, GI: genetic instrument, SNP: Single Nucleotide Polymorphism, OR: odds ratio, HR: hazard ratio, SD: standard deviation, GRS: genetic risk score, BMI: body mass index, IVW: inverse variance weighted, EPA: Eicosapentaenoic acid, DHA: Docosahexaenoic acid, DPA: Docosapentaenoic acid, AA: arachidonic acid, DGLA: dihomo-γ-linolenic acid, LA: linoleic acid, ALA: α-Linolenic acid, HDL: high-density lipoprotein cholesterol, LD: linkage disequilibrium, LDL: low-density lipoprotein cholesterol, IL-6: interleukin 6, HbA1C: glycated hemoglobin, IGF-1: insulin-like growth factor 1, GDF-15: Growth differentiation factor 15. | | | | | | | | | | | | | | | |

| **Supplementary Table 4 General characteristics of 145 meta-analyses of observational studies** | | | | | | | | | | | |
| --- | --- | --- | --- | --- | --- | --- | --- | --- | --- | --- | --- |
| First author/Year (citation) | Population | Location | Biomarker | No of cases | No of participants | Unit of comparison | No of studies | Study design | Metric | Model | Effect size (95%CI) |
| **Fatty acid/Lipid metabolism biomarkers** | | | | | | | | | | | |
| Yang B,2014^1^ | CRC & controls | America Europe Asia | Adipose DHA 22:6 (n-3) | 93 | 204 | Mean (% total fatty acids) | 2 | CC | SMD | Random | 0.19(-0.09, 0.47) |
| Yang B,2014^1^ | CRC & controls | America Europe Asia | Adipose DPA 22:5 (n-3) | 93 | 204 | Mean (% total fatty acids) | 2 | CC | SMD | Random | -0.02(-0.35, 0.30) |
| Yang B,2014^1^ | CRC & controls | America Europe Asia | Adipose EPA 20:5 (n-3) | 93 | 204 | Mean (% total fatty acids) | 2 | CC | SMD | Random | -0.07(-0.35, 0.20) |
| Yang B,2014^1^ | General/CRC & controls | America Europe Asia | Biospecimen DHA 22:6 (n-3) | 1,315 | 84,114 | Highest vs lowest categories | 6 | CC/CS | RR | Random | 0.68(0.54, 0.84) |
| Yang B,2014^1^ | CRC & controls | America Europe Asia | Biospecimen DHA 22:6 (n-3) | 623 | 1,938 | Mean (% total fatty acids) | 7 | CC | SMD | Random | -0.23(-0.34, -0.11) |
| Yang B,2014^1^ | General/CRC & controls | America Europe Asia | Biospecimen DHA 22:6 (n-3) | 675 | 58,713 | Highest vs lowest categories | 3 | CS | RR | Random | 0.76(0.56, 1.01) |
| Yang B,2014^1^ | General/CRC & controls | America Europe Asia | Biospecimen DPA 22:5 (n-3) | 446 | 15,593 | Highest vs lowest categories | 3 | CC/CS | OR | Random | 0.80(0.42, 1.52) |
| Yang B,2014^1^ | CRC & controls | America Europe Asia | Biospecimen DPA 22:5 (n-3) | 587 | 1,404 | Mean (% total fatty acids) | 6 | CC | SMD | Random | -0.08(-0.22, 0.06) |
| Yang B,2014^1^ | General/CRC & controls | America Europe Asia | Biospecimen EPA 20:5 (n-3) | 1,367 | 84,223 | Highest vs lowest categories | 6 | CC/CS | RR | Random | 0.78(0.64, 0.96) |
| Yang B,2014^1^ | CRC & controls | America Europe Asia | Biospecimen EPA 20:5 (n-3) | 623 | 1,938 | Mean (% total fatty acids) | 7 | CC | SMD | Random | -0.27(-0.41, -0.13) |
| Yang B,2014^1^ | General/CRC & controls | America Europe Asia | Biospecimen EPA 20:5 (n-3) | 675 | 58,713 | Highest vs lowest categories | 3 | CS | RR | Random | 0.77(0.58, 1.00) |
| Yang B,2014^1^ | General/CRC & controls | America Europe Asia | Biospecimens LC n-3 PUFA | 1,502 | 60,360 | Highest vs lowest categories | 7 | CC/CS | RR | Random | 0.74(0.63, 0.87) |
| Yang B,2014^1^ | CRC & controls | America Europe Asia | Biospecimens LC n-3 PUFA | 329 | 786 | Mean (% total fatty acids) | 4 | CC | SMD | Random | 0.22(0.07, 0.37) |
| Yang B,2014^1^ | General/CRC & controls | America Europe Asia | Biospecimens LC n-3 PUFA | 675 | 58,713 | Highest vs lowest categories | 3 | CS | RR | Random | 0.76(0.59, 0.97) |
| Yang B,2014^1^ | CRC & controls | America Europe Asia | Blood DHA 22:6 (n-3) | 646 | 1,425 | Mean (% total fatty acids) | 5 | CC | SMD | Random | -0.24(-0.40, -0.10) |
| Yang B,2014^1^ | CRC & controls | America Europe Asia | Blood DPA 22:5 (n-3) | 646 | 1,425 | Mean (% total fatty acids) | 4 | CC | SMD | Random | 0.04(- 0.11,0.20) |
| Yang B,2014^1^ | CRC & controls | America Europe Asia | Blood EPA 20:5 (n-3) | 283 | 564 | Mean (% total fatty acids) | 5 | CC | SMD | Random | -0.30(-0.44, -0.15) |
| Esposito K,2013^2^ | General/CRC & controls | America Europe Asia | HDL-cholesterol | 1,335 | / | Highest vs lowest categories (mg/dl) | 9 | CC/CS | RR | Random | 0.89(0.78, 1.02) |
| Yao X,2015^3^ | General/CRC & controls | North America, Europe, and Asia | HDL-cholesterol | 2,542 | 136,698 | Highest vs lowest categories | 6 | PS | RR | Random | 0.84(0.69, 1.02) |
| Yao X,2015^3^ | General/CRC & controls | North America, Europe, and Asia | LDL-cholesterol | 1,626 | 9,175 | Highest vs lowest categories | 3 | PS | RR | Random | 1.04(0.60, 1.81) |
| Yao X,2015^3^ | General/CRC & controls | North America, Europe, and Asia | Total cholesterol | 10,892 | 7,725,310 | Highest vs lowest categories | 10 | PS | RR | Random | 1.11(1.01, 1.21) |
| Yao X,2015^3^ | General/CRC & controls | North America, Europe, and Asia | Total cholesterol | / | / | 100 mg/dL increment | 5 | PS | RR | Random | 1.01(0.97, 1.05) |
| Esposito K,2013^2^ | General/CRC & controls | America Europe Asia | Triglycerides | 8,164 | / | Highest vs lowest categories (mg/dl) | 13 | CC/CS | RR | Random | 1.06(0.95, 1.17) |
| Yao X,2015^3^ | General/CRC & controls | North America, Europe, and Asia | Triglycerides | 8,127 | 2,252,217 | High versus low | 9 | PS | RR | Random | 1.18(1.04, 1.34) |
| Yao X,2015^3^ | General/CRC & controls | North America, Europe, and Asia | Triglycerides | / | / | 50 mg/dL increment | 3 | PS | RR | Fixed | 1.01(1.00, 1.03) |
| **Infectious agents** | | | | | | | | | | | |
| Repass J,2018^4^ | CRC patients (CRC sample with normal tissue) | North America | F. nucleatum | 139 | 139 | CRC tissue to adjacent normal tissue | 2 | CC | r | Random | 0.38(0.17, 0.56) |
| Liu C,2016^20^ | H.pylori-infected patients and controls | America Europe Asia | H. pylori infection | 5,380 | 17,189 | CRC vs non-CRC | 19 | NCC/CC/CSS | OR | Random | 1.57(1.29, 1.83) |
| Wang F,2014^6^ | CRC & Controls | America Europe Asia | H. pylori infection | 3,450 | 10,808 | Infected vs non-infected | 17 | CC | OR | Fixed | 1.28(1.16, 1.41) |
| Wang F,2014^6^ | CRC & Controls | America Europe Asia | H. pylori infection_CagA | / | / | CRC vs non-CRC | 6 | CC | OR | Fixed | 1.22(1.08, 1.37) |
| Wang X,2017^7^ | H.pylori infected and controls | / | H. pylori infection | 3,300 | 16,857 | CRC vs non-CRC | 10 | CC/CSS | OR | Random | 1.29(1.06, 1.51) |
| Zhao Y,2016^8^ | CRC & Controls | America Europe Asia Australia | H. pylori infection | 1,457 | 3,762 | CRC vs non-CRC | 14 | CC | OR | Random | 1.33(1.01, 1.77) |
| Wu Q,2013^9^ | CRC & Controls | America Europe Asia | H. pylori infection | 3,488 | 7,836 | CRC vs non-CRC | 20 | CC/NCC/CSS | OR | Random | 1.39(1.18, 1.64) |
| Rokkas T,2013^10^ | CRC & Controls | America Europe Asia | H. pylori infection | / | / | Infected vs non-infected | 17 | CC/CSS | OR | Random | 1.30(1.07, 1.59) |
| Guo Y,2014^11^ | CRC & Controls | Asia | H. pylori infection | / | / | Infected vs non-infected |  | CC | OR | Random | 1.08(0.89, 1.68) |
| Zhao YS, 2008^12^ | CRC & Controls | America Europe Asia | H. pylori infection | 1,709 | 3,581 | CRC vs controls | 14 | CC/NCC | OR | Random | 1.49(1.17, 1.91) |
| Baandrup L,2017^13^ | CRC/CRA tissue and tumour adjacent tissue or cancer free control tissue | America Europe Asia | HPV | 699 | 1,156 | Tumour tissue vs normal tissue | 8 | CC | OR | Random | 2.10(1.30, 3.20) |
| Baandrup L,2017^13^ | CRC/CRA tissue and tumour adjacent tissue or cancer free control tissue | America Europe Asia | HPV | 419 | 575 | Infected vs non-infected | 8 | CC | OR | Random | 6.00(2.00, 17.90) |
| Damin DC,2013^14^ | CRC & controls | America Europe Asia | HPV | 289 | 393 | Infected vs non-infected | 5 | CC | OR | Random | 10.04(3.67, 27.46) |
| Damin DC,2013^14^ | CRC & Controls | America Europe Asia | HPV | / | / | Tumour tissue vs normal tissue | 6 | CC | OR | Random | 4.05(1.79, 9.14) |
| Pelizzer T,2016^15^ | HPV & Controls | America Europe Asia | HPV | 126 | 612 | CRC vs non-CRC | 4 | CC/CSS | OR | Random | 4.66(2.50, 8.69) |
| Zhang XH,2018^16^ | CRC & Controls | China | HPV | 766 | 1,236 | Tumour tissue vs normal tissue | 10 | CC | OR | Random | 10.78(4.22, 27.53) |
| Peder LD,2018^17^ | CRC & Controls | Brazil | HPV | 216 | / | Infected vs control | 2 | CC | RR | Random | 2.03(1.77, 2.33) |
| Ibragimova MK,2018^18^ | CRC & Controls | America Europe Asia | HPV | 2,049 | 2,879 | Tumour tissue vs Cancer-free tissue | 19 | CC | RR | Random | 2.97(1.42, 6.22) |
| Bai B,2016^19^ | CRC patients | America Europe Asia | Human cytomegalovirus infection | 480 | 960 | Tumour tissue vs normal tissue | 4 | CC | OR | Fixed | 6.59(4.48, 9.69) |
| Liu H,2016^5^ | CRC & Controls | Europe Asia | Bacteroides-Prevotella group | 97 | 255 | Healthy controls (logarithmic number of bacteria per gram stool) | 3 | CC | SMD | Random | -0.71(-2.56, 1.14) |
| Liu H,2016^5^ | CRC & Controls | Europe Asia | Bifidobacterium | 127 | 315 | Healthy controls (logarithmic number of bacteria per gram stool) | 4 | CC | SMD | Random | -3.3(-6.57, -0.03) |
| Liu H,2016^5^ | CRC & Controls | Europe Asia | Enterobacteriaceae | 37 | 76 | Healthy controls (logarithmic number of bacteria per gram stool) | 2 | CC | SMD | Random | 2.69(0.66, 4.72) |
| Liu H,2016^5^ | CRC & Controls | Europe Asia | Escherichia coli | 90 | 239 | Healthy controls (logarithmic number of bacteria per gram stool) | 2 | CC | SMD | Random | 1.4(-1.38, 4.18) |
| Liu H,2016^5^ | CRC & Controls | Europe Asia | Faecalibacterium prausnitzii | 86 | 233 | Healthy controls (logarithmic number of bacteria per gram stool) | 3 | CC | SMD | Random | -0.33(-0.6, 0.05) |
| Liu H,2016^5^ | CRC & Controls | Europe Asia | Lactobacillus | 127 | 315 | Healthy controls (logarithmic number of bacteria per gram stool) | 4 | CC | SMD | Random | -2.72(-5.94, 0.50) |
| Liu H,2016^5^ | CRC & Controls | Europe Asia | Total bacteria | 86 | 233 | Healthy controls (logarithmic number of bacteria per gram stool) | 3 | CC | SMD | Random | 0.23(-0.54, 1.00) |
| Boleij A,2011^21^ | S. bovis–infected patients | / | Streptococcus bovis | 189 | 340 | CRC vs non-CRC | 6 | case-series | OR | Fixed | 7.26(3.94, 13.36) |
| Krishnan S,2014^22^ | CRC & controls | / | Streptococcus bovis in faeces | 148 | 414 | / | 3 | CC | OR | Random | 2.46(0.72, 8.46) |
| **Inflammatory Markers** | | | | | | | | | | | |
| Guo YZ,2013^23^ | General | America Europe | CRP | 1,140 | 135,794 | Per natural log unit change | 7 | CS | HR | Random | 1.10(0.97, 1.23) |
| Zhou B,2014^24^ | General/CRC & controls | America Europe Asia | CRP | 4,779 | 152,942 | 1-unit change in ln(mg/l) | 17 | CS/NCC | RR | Random | 1.12(1.05, 1.21) |
| Tsilidis KK, 2008^25^ | General/CRC & controls | America Europe Asia | CRP | 1,159 | 39,145 | 1-unit increase in ln-transformed (mg/L) | 8 | NCC/CS | OR | Random | 1.12(1.01, 1.25) |
| Zhou B,2014^24^ | General/CRC & controls | America Europe Asia | IL-6 | 1,125 | 9,909 | 1-unit change in ln (pg/ml) | 6 | CS/NCC | RR | Random | 1.10(0.88, 1.36) |
| Kakourou A,2015^26^ | General/CRC & controls | America Europe | IL-6 | 1,308 | 9,728 | Per 1 U change in ln pg/mL | 7 | CC/CS/NCC | RR | Random | 1.10(0.94, 1.28) |
| **Insulin related biomarkers** | | | | | | | | | | | |
| Xu J,2016^27^ | CRC & Controls | America Europe Asia | C peptide | 3,191 | 1,375,980 | Highest vs lowest categories(ng/ml) | 9 | CC/NCC | RR | Random | 1.27(1.08, 1.49) |
| Pisani, 2008^28^ | General/CRC & controls | / | C peptide | 1,309 | 5,542 | Highest vs lowest categories | 12 | CC/NCC/CS | RR | Fixed | 1.35(1.13, 1.61) |
| Chen L,2013^29^ | CRC & Controls | America Europe Asia | C peptide | 3,109 | 7,394 | Highest vs lowest categories | 9 | NCC | OR | Random | 1.39(1.04, 1.87) |
| Xu J,2016^27^ | General/CRC & controls | America Europe Asia | HbA1c | 2,137 | 820,317 | Highest vs lowest categories (%) | 8 | CC/NCC/CS | RR | Fixed | 1.22(1.02, 1.47) |
| Xu J,2016^27^ | General/CRC & controls | America Europe Asia | HOMA-IR | 2,956 | 347,326 | Highest vs lowest categories (fasting glucose (mmol/L) × fasting insulin (mIU/L) / 22.5) | 8 | CC/NCC/CS | OR | Fixed | 1.47(1.24, 1.74) |
| Xu J,2016^27^ | General/CRC & controls | America Europe Asia | Fasting insulin | 3,239 | 354,870 | Highest vs lowest categories (mIU/L) | 10 | CC/NCC/CS | OR | Fixed | 1.42(1.19, 1.69) |
| Xu J,2016^27^ | General/CRC & controls | America Europe Asia | Fasting glucose | 17,764 | 3,805,861 | Highest vs lowest categories (mmol/L) | 18 | CC/NCC/CS | OR | Random | 1.12(1.06, 1.18) |
| Shi J,2015^30^ | General/CRC & controls | / | Fasting glucose | 62,814 | 2,969,306 | Per 20 mg/dL increase | 6 | CC/CS/NCC | RR | Fixed | 1.02(1.01, 1.02) |
| Shi J,2015^30^ | General/CRC & controls | / | Fasting glucose | 62,814 | 2,969,306 | Highest vs lowest categories (FPG category ≥ 3) | 6 | CC/CS/NCC | RR | Fixed | 1.15(1.02, 1.31) |
| Shi J,2015^30^ | General/CRC & controls | / | Fasting glucose | / | / | Highest vs lowest categories (FPG category ≥=2) | 5 | CC/CS/NCC | RR | Random | 1.57(1.31, 1.89) |
| Crawley DJ,2014^31^ | / | America Europe Asia | Fasting glucose | 908 | / | ‘high’ and ‘normal’ <6.1 mmol/L cut off | 5 | CC/CS | RR | Random | 1.35(1.21, 1.51) |
| Pisani, 2008^28^ | General/CRC & controls | / | Fasting glucose | 1,741 | 1,381,741 | Highest vs lowest categories | 11 | CC/NCC/CS | RR | Fixed | 1.18(1.07, 1.31) |
| Chi F,2013^32^ | CRC & Controls | Caucasians, Asian, mixed population | IGF 1 | 3,807 | 11,613 | Highest vs lowest categories | 16 | CC/NCC | OR | Fixed | 1.25(1.08, 1.45) |
| Rinaldi S,2010^33^ | CRC & Controls | Europe | IGF I | 1,741 | 5,586 | 1 standard deviation change of average IGF-I distribution | 10 | CC/CS | RR | Random | 1.10(1.01, 1.19) |
| Morris J, 2006^34^ | CRC & Controls | America, Europe, Asia | IGF 1 | 1,106 | 3,501 | Highest vs lowest categories | 7 | NCC | OR | Random | 1.37(1.05, 1.78) |
| Chi F,2013^32^ | CRC & Controls | Caucasians, Asian, mixed population | IGF 2 | 783 | 4,007 | Highest vs lowest categories | 6 | CC/NCC | OR | Fixed | 1.52(1.16, 2.01) |
| Morris J, 2006^34^ | CRC & Controls | America, Europe, Asia | IGF 2 | 384 | 1,685 | Highest vs lowest categories | 3 | NCC | OR | Random | 1.95(1.26, 3.00) |
| Chi F,2013^32^ | CRC & Controls | Caucasians, Asian, mixed population | IGFBP 1 | 2,154 | 6,439 | Highest vs lowest categories | 7 | CC/NCC | OR | Fixed | 0.85(0.70, 1.03) |
| Chi F,2013^32^ | CRC & Controls | Caucasians, Asian, mixed population | IGFBP 2 | 1,348 | 2,962 | Highest vs lowest categories | 3 | CC/NCC | OR | Random | 0.77(0.41, 1.43) |
| Chi F,2013^32^ | CRC & Controls | Caucasians, Asian, mixed population | IGFBP 3 | 3,755 | 11,509 | Highest vs lowest categories | 15 | CC/NCC | OR | Random | 0.88(0.71, 1.10) |
| Morris J, 2006^34^ | CRC & Controls | America, Europe, Asia | IGFBP 3 | 1,106 | 3,501 | Highest vs lowest categories | 7 | NCC | OR | Random | 0.98(0.64, 1.51) |
| **Micronutrients** | | | | | | | | | | | |
| Chuang SC,2013^35^ | CRC & Controls | America Europe | Folate | 3,477 | 10,516 | Dose-response (per 10 nmol/L ) | 8 | CS | RR | Fixed | 0.94(0.88, 1.01) |
| Chuang SC,2013^35^ | CRC & Controls | America Europe | Folate | 3,477 | 10,516 | Highest vs lowest categories | 8 | CS | RR | Fixed | 0.91(0.77, 1.05) |
| Shiao SPK,2018^36^ | CRC & Controls | Europe, Asia | Folate | 1,466 | 3,393 | Mean (nmol/L) | 8 | CC | SMD | Random | -0.46(-0.92, -0.00) |
| Shiao SPK,2018^36^ | CRC & Controls | Europe, Caucasian, Asia | Folate | 3,515 | 8,764 | Mean (nmol/L) | 9 | CS | SMD | Random | 0.01(-0.06, 0.08) |
| Moazzen S,2017^37^ | CRC & Controls | / | Folate | / | / | / | 22 | CC | RR | Random | 0.85(0.85, 1.30) |
| Zhang D, 2015^38^ | CRC & Controls | / | Folate | 1,181 | 3,139 | CRC vs healthy controls | 11 | CC | SMD | Random | -1.10(-1.60, -0.60) |
| Shiao SPK,2018^36^ | CRC & Controls | Europe, Asia | Vitamin B12 | 360 | 1,068 | Mean (pmol/L) | 6 | CC | SMD | Random | -0.99(-1.74, 0.25) |
| Shiao SPK,2018^36^ | CRC & Controls | Europe, Caucasian | Vitamin B12 | 3,299 | 8,309 | Mean (pmol/L) | 8 | CS | SMD | Random | -0.04(-0.09, 0.00) |
| Sun NH,2016^39^ | CRC & Controls | Europe USA | Vitamin B12 | / | / | Per 150 pmol/l increment | 3 | CC | RR | Random | 1.02(0.88, 1.19) |
| Sun NH,2016^39^ | CRC & Controls | Europe USA | Vitamin B12 | 682 | 1,732 | Highest vs lowest categories | 3 | CC | RR | Fixed | 0.93(0.56, 1.53) |
| Zhang D, 2015^38^ | CRC & Controls | / | Vitamin B12 | 873 | 2,198 | CRC vs healthy controls | 10 | CC | OR | Random | -28.52(-50.60, -6.43) |
| Shiao SPK,2018^36^ | CRC & Controls | Globe | Vitamin B2 | 1,643 | 4,240 | Mean (nmol/L) | 3 | CS | SMD | Random | 0.00(-0.05, 0.07) |
| Ben S, 2018^40^ | General/CRC & controls | Europe | Vitamin B2 | 1,593 | 32,962 | Highest vs lowest categories | 2 | NCC | RR | Fixed | 0.74(0.59, 0.92) |
| Shiao SPK,2018^36^ | CRC & Controls | Globe | Vitamin B6 | 2,658 | 7,361 | Mean (nmol/L) | 5 | CS | SMD | Random | -0.06(-0.11, 0.01) |
| Larsson SC,2010^41^ | General | America Europe | Vitamin B6 | 883 | 2,207 | Highest vs lowest categories (pmol/) | 4 | PS | RR | Random | 0.52(0.38, 0.71) |
| Larsson SC,2010^41^ | General | America Europe | Vitamin B6 | 883 | 2,207 | 100pmol/mL increment | 4 | PS | RR | Random | 0.51(0.38, 0.69) |
| Mocellin S,2017^42^ | CRC & Controls | Europe USA | Vitamin B6 | 425 | / | Highest vs lowest categories | 5 | RS | RR | Random | 0.56(0.46, 0.67) |
| Mocellin S,2017^42^ | CRC & Controls | Europe USA | Vitamin B6 | 2,203 | / | Per 100 nmol/L decrease | 5 | RS | RR | Random | 0.52(0.43, 0.64) |
| Vinceti M,2014^43^ | General | US Netherlands Finland | selenium | 762 | 383,137 | Highest vs lowest categories | 5 | CS/NCC | OR | Random | 0.89(0.65, 1.23) |
| Vinceti M,2018^44^ | General | Europe USA | selenium | 2,627 | 712,746 | Highest vs lowest categories | 5 | CS/NCC | OR | / | 0.82(0.72, 0.94) |
| Chung M,2011^45^ | General/CRC & controls | / | 25-hydroxyvitamin D | 1,127 | 2,249 | Per 10-nmol/L increase | 9 | NCC | OR | Random | 0.94(0.91, 0.97) |
| Ekmekcioglu C,2017^46^ | CRC & Controls | Europe USA | 25-hydroxyvitamin D | / | / | 20–30 ng/mL 25(OH)D status vs those in the lowest category | 24 | CC/CS | RR | Fixed | 0.83(0.76, 0.90) |
| Ekmekcioglu C,2017^46^ | CRC & Controls | Europe USA | 25-hydroxyvitamin D | / | / | Highest vs lowest categories | 24 | CC/CS | RR | Fixed | 0.62(0.56, 0.70) |
| Gandini S,2011^47^ | General/CRC & controls | Europe USA | 25-hydroxyvitamin D | 2,630 | / | 10 ng/ml increase | 9 | CC/NCC | SRR | Random | 0.85(0.79, 0.91) |
| Garland CF,2017^48^ | CRC & Controls | / | 25-hydroxyvitamin D | 6,691 | 175,127 | Highest vs lowest categories | 15 | NCC | OR | Random | 0.67(0.59, 0.76) |
| Ma Y,2011^49^ | CRC & controls | America Europe Asia | 25-hydroxyvitamin D | 3,142 | 7,840 | Highest vs lowest categories | 9 | CS/NCC | RR | Random | 0.67(0.54, 0.80) |
| Ma Y,2011^49^ | CRC & controls | America Europe Asia | 25-hydroxyvitamin D | 2,767 | 6,715 | 10 ng/mL increment | 9 | CS/NCC | RR | Random | 0.74(0.63, 0.89) |
| Touvier M,2011^50^ | CRC & Controls | / | 25-hydroxyvitamin D | 2,318 | / | Per 100 IU/L increment | 6 | NCC | RR | Random | 0.96(0.94, 0.97) |
| Lee JE,2011^51^ | CRC & Controls | America Europe Asia | 25-hydroxyvitamin D | 2,622 | 6,560 | Highest vs lowest categories | 9 | PS | OR | Random | 0.66(0.54, 0.81) |
| Lee JE,2011^51^ | CRC & Controls | America Europe | 1,25-dihydroxyvitamin D | 625 | 1,801 | Highest vs lowest categories | 4 | PS | OR | Random | 1.01(0.59, 1.73) |
| Yin L, 2009^52^ | General/CRC & controls | America Europe Asia | 25-hydroxyvitamin D | 984 | 2,944 | Per 20 ng ⁄mL increase in serum 25(OH)D | 7 | NCC/CS | OR | Random | 0.57(0.43, 0.76) |
| Dong Y,2017^53^ | General/CRC & controls | Caucasian Asian | Vitamin E | 520 | 6,440 | Mean (µmol/L) | 10 | CC | WMD | Random | -3.00(-4.40, -1.59) |
| Gumulec J,2014^54^ | CRC & Controls | / | Serum Zinc | 313 | 529 | Largest variation among serum levels | 5 | CC | SMD | Random | 0.04(-2.57, 2.64) |
| Gumulec J,2014^54^ | CRC & Controls | / | Tissue Zinc | 234 | 398 | Largest variation among tissue levels | 7 | CC | SMD | Random | 0.37(-0.97, 1.72) |
| **Other Biomarkers** | | | | | | | | | | | |
| Jiang R,2016^55^ | CRC & Controls | Canada Europe Asia | Enterolactone | 762 | 2,408 | Per doubling (nmol/l) | 3 | PS | RR | Random | 1.04(0.98, 1.10) |
| Zhang BL,2015^56^ | General/CRC & controls | / | Blood group A | 6,931 | 3,214,941 | A vs non-A | 8 | CC/CS | OR | Fixed | 1.05(0.98, 1.13) |
| Zhang BL,2015^56^ | General/CRC & controls | / | Blood group AB | 6,931 | 3,191,289 | AB vs non-AB | 8 | CC/CS | OR | Random | 1.07(0.88, 1.26) |
| Zhang BL,2015^56^ | General/CRC & controls | / | Blood group B | 6,931 | 3,193,532 | B vs non-B | 8 | CC/CS | OR | Random | 1.12(0.94, 1.29) |
| Zhang BL,2015^56^ | General/CRC & controls | / | Blood group O | 6,931 | 3,219,151 | O vs non-O | 8 | CC/CS | OR | Random | 0.89(0.81, 0.96) |
| Zhang X,2017^57^ | General | Europe, North America | Telomere Length | 319 | / | Longest or shortest TL group | 2 | PS | OR | Random | 1.01(0.68, 1.50) |
| Naing C,2017^58^ | General/CRC & controls | America Europe Asia | Telomere Length | 951 | 2,569 | Shortest Q4 vs longest Q1 | 4 | NCC | OR | Fixed | 1.01(0.77, 1.34) |
| Naing C,2017^58^ | General/CRC & controls | America Europe Asia | Telomere Length | 4,000 | 10,375 | Shortest Q4 vs longest Q1 | 4 | CC | OR | Random | 1.65(0.96, 2.83) |
| **Protein&amino acids** | | | | | | | | | | | |
| Lu S,2017^59^ | CRC & Controls | Caucasian | Adiponectin | 4,076 | 9,585 | Highest vs lowest categories | 8 | NCC | RR | Fixed | 0.81(0.71, 0.93) |
| Lu S,2017^59^ | CRC & Controls | Caucasian | Adiponectin | 4,076 | 9,585 | Per 5 µg/mL increase (dose reponse) | 8 | NCC | RR | Fixed | 0.86(0.77, 0.95) |
| Lu W,2018 ^60^ | CRC & Controls | / | Adiponectin | 7,554 | 17,352 | Mean (µg/mL) | 31 | CC | WMD | Random | -0.76(-1.20, -0.32) |
| Xu XT,2011^61^ | CRC & Controls | America Europe Asia | Adiponectin | 1,343 | 2,996 | Mean (µg/mL) | 11 | CC/CSS/NCC | WMD | Random | -1.08(-1.84, -0.33) |
| Joshi RK,2014^62^ | CRC & Controls | / | Adiponectin | 3,416 | 8,265 | μg/mL | 10 | CC/CSS/NCC | OR | Random | 1.03(0.72, 1.47) |
| An W,2012^63^ | CRC & Controls | America Europe Asia | Adiponectin | 488 | 827 | Mean (µg/mL) | 10 | CC/NCC | WMD | Random | -1.51(-2.42, -0.59) |
| Joshi RK,2014^62^ | CRC & Controls | / | Adiponectin | 3,416 | 11,681 | Reference group and compared group (μg/mL) | 9 | CC/NCC/CSS | OR | Fixed | 0.91(0.83, 1.00) |
| Yu DH,2018^64^ | CRC & Controls | Japan UK | Angiogenin | 188 | 240 | Mean (ng/ml) | 2 | CC | SMD | Random | 1.54(0.50, 2.59) |
| Yang G,2016^65^ | CRC & Controls | / | Resistin | 965 | 3,255 | Mean (ng/mL) | 11 | CC/CS | WMD | Random | 1.47(0.78, 2.16) |
| Joshi RK,2014^62^ | CRC & Controls | / | Leptin | / | / | ng/mL | 9 | CC/CSS/NCC | OR | Random | 1.36(0.95, 1.94) |
| Gialamas SP,2013^66^ | CRC & Controls | America Europe Asia | Leptin | 3,508 | 7,478 | Mean | 23 | CC/CS/CSS/NCC | SMD | Random | 0.18(-0.04, 0.40) |
| Gialamas SP,2013^66^ | CRC & Controls | Europe Asia | Leptin | / | / | Highest vs lowest categories | 10 | CC/CSS/NCC | RR | Random | 1.04(0.65, 1.65) |
| Joshi RK,2014^62^ | CRC & Controls | / | Leptin | 2,597 | 6,077 | Reference group and compared group (ng/mL) | 8 | CC/NCC/CSS | OR | Random | 1.06(0.87, 1.28) |
| Feng Z, 2015^67^ | CRC & Controls | Europe Asia | Ferritin | 277 | 927 | Mean (ng/ml) | 6 | CC | SMD | Random | -1.57(-2.72, -0.42) |
| Shiao SPK,2018^36^ | CRC & Controls | Europe, Asia | Homocysteine | 2,438 | 5,419 | Mean (mmol/L) | 10 | CC | SMD | Random | 0.71(0.41, 1.02) |
| Shiao SPK,2018^36^ | CRC & Controls | Europe, Caucasian | Homocysteine | 4,047 | 9,604 | Mean (mmol/L) | 8 | CS | SMD | Random | 0.11(0.02, 0.21) |
| Zhang DH, 2015^38^ | CRC & Controls | / | Homocysteine | 3,954 | 8,992 | CRC vs healthy controls | 22 | CC | OR | Random | 2.63 (1.74, 3.51) |
| Shiao SPK,2018^36^ | CRC & Controls | Europe | Methionine | 1,980 | 5,493 | Mean (mmol/L) | 2 | CC | SMD | Random | -0.29(-0.56, 0.02) |
| Sun SJ,2016^68^ | CRC & Controls | Asia Caucasians Africa | HER‑2(human epidermal growth factor receptor 2) expression | 932 | 1,453 | Categories | 13 | CC | OR | Random | 10.44(5.50, 19.81) |
| Xing XJ,2014^69^ | CRC & Controls | Europe Asia | MMP7 | 625 | 1,020 | Mean (% total fatty acids) | 7 | CC | SMD | Random | 2.15(1.46, 2.84) |
| Li XX,2014^70^ | CRC & Controls | Asian | TLR-4 protein | 168 | 283 | CRC vs healthy controls | 3 | CC | OR | Random | 4.75(1.16, 19.36) |
| Ouyang Z,2017^71^ | CRC & Controls | Asian Caucasian | CD26 | 952 | 1,809 | Tumour cell vs normal cell | 7 | CC | SMD | Random | -0.33(-2.97, 2.30) |
| Ouyang Z,2017^71^ | CRC & Controls | Asian | CD26 | 239 | 421 | Tumour cell vs normal cell | 3 | CC | SMD | Random | 3.94(1.55, 6.33) |
| Ouyang Z,2017^71^ | CRC & Controls | Caucasian | CD26 | 475 | 1,150 | Tumour cell vs normal cell | 4 | CC | SMD | Random | -3.77(-7.67, 0.12) |
| CRC: colorectal cancer, RR: risk ratio, SMD: standard mean difference, OR: odds ratio, r: standardized correlation coefficient, CC: case-control study, CS: cohort study, NCC: nested case-control study, CSS: cross-sectional study, PS: prospective study, LC n-3 PUFA: long chain n-3 polyunsaturated fatty acid, EPA: Eicosapentaenoic acid, DHA: Docosahexaenoic acid, DPA: Docosapentaenoic acid, HDL: high-density lipoprotein cholesterol, LDL: low-density lipoprotein cholesterol, H.pylori: helicobacter pylori , HPV: Human papillomavirus, CRP: C-reactive protein, IL-6: Interleukin 6, HOMA-IR: homeostatic model assessment-insulin resistance, IGF 1/2: Insulin-like growth factor 1/2, IGFBP 1/2/3: Insulin-like growth factor-binding protein 1/2/3, HbA1c: glycated hemoglobin, MMP7: matrix metalloproteinase-7, CD26: dipeptidyl peptidase IV. | | | | | | | | | | | |

| **Supplementary Table 5 Characteristics and main findings of 66 Mendelian randomisation analyses** | | | | | | | | | | | | | | | |
| --- | --- | --- | --- | --- | --- | --- | --- | --- | --- | --- | --- | --- | --- | --- | --- |
| First author, Year (Citation) | Biomarker (Unit) | Power to detect given effect estimates | Exposure | | | Outcome | | | Study design | Main method | Main estimate | P value | Sensitivity analyses | Consistent evidence of a causal effect | Evidence |
|  |  |  | Sample size | Population | Variance (R^2^) explained by GI (%) | Sample size | Metric | Population |  |  |  |  |  |  |  |
| Micronutrients | | | | | | | | | | | | | | | |
| He Y,2018^72^ | 25-hydroxyvitamin D (log-transformed nmol/L) | 0.72\|OR=0.83 | 2,821 participants | European | 2.84 | 10,725 cases, 30,794 controls | OR per SD | European | One-sample | Coefficient ratio method (Weighted GRS adjusted age, sex and BMI) | 1.03(0.51,2.07) | 0.931 | Without adjustment for age, sex and BMI; unweighted GRS | No | Unknown |
| He Y,2018^72^ | 25-hydroxyvitamin D (log-transformed nmol/L) | 0.93\|OR=0.83 | 77,354 participants | European | 2.84 | 18,967 cases,48,168 controls | OR per SD | European | Two-sample | IVW | 0.91(0.69,1.19) | 0.475 | Weighted median, MR-Egger, robust regression, as well as linear regression, different combinations of SNPs | No | Unknown |
| Chandler PD, 2018^73^ | 25-hydroxyvitamin D (GRS) | / | 1,782 participants | European women | / | 329 cases and 23,294 participants | HR per score unit | European women | One-sample | Age-adjusted Cox-proportional hazard regression by using unweighted GRS (continuous) | 1.06(1.00,1.13 ) | 0.070 | Categorical assessment of the GRS (0–5 (reference group), 6–7 and 8–10 points), adjustment for BMI, exclusion of one SNP and reporting HR per 20 nmol/L increase in 25(OH)D | No | Unknown |
| Dimitrakopoulou V, 2017^74^ | 25-hydroxyvitamin D (weighted multi-polymorphism score) | ≥0.8\|OR≤0.85 (OR≥1.18) (R^2^=0.05) or OR≤0.81 (OR≥1.23) (R^2^=0.03) | 4501 & 33,996 participants | European | 3 to 5 | 11488 cases and 11679 controls | OR per 25nmol/L increase | European | Two-sample | IVW and likelihood-based method | IVW: 0.92(0.76,1.10) Likelihood: 0.92 (0.76, 1.10) | IVW: 0.36; Likelihood: 0.36 | Colorectal cancer in men and women, colon cancer, rectal cancer, proximal colon cancer, distal colon cancer; MR-Egger, weighted median approach, and over-identification tests; Two separate allelic scores: vitamin D synthesis allele score and metabolism allele score | No | Unknown |
| Dimitrakopoulou V, 2017^74^ | 25-hydroxyvitamin D (weighted multi-polymorphism score) | ≥0.8\|OR≤0.85 (OR≥1.18) (R^2^=0.05) or OR≤0.81 (OR≥1.23) (R^2^=0.03) | 4501 & 33,996 participants | European | 3 to 5 | 5100 cases and 4831 controls | OR per 25nmol/L increase | European | Two-sample | IVW and likelihood-based method | IVW: 1.04 (0.78, 1.38); Likelihood: 1.04 (0.78, 1.38) | IVW: 0.81; Likelihood: 0.81 | Colorectal cancer in men and women, colon cancer, rectal cancer, proximal colon cancer, distal colon cancer; MR-Egger, weighted median approach, and over-identification tests; Two separate allelic scores: vitamin D synthesis allele score and metabolism allele score | No | Unknown |
| Theodoratou E, 2012^75^ | 25-hydroxyvitamin D (GRS) | <0.35 | 2001 cases and 2237 controls | Scottish | 3.6-5.2 | 2001 cases and 2237 controls | OR per 1ng/ml increase | Scottish | One-sample | Logistic regression | 1.16 (0.60,2.23) | / | Unadjusted age and sex; formed three different allele scores | No | Unknown |
| Ong JS,2018^76^ | 25-hydroxyvitamin D | / | 4501 & 33,996 & 8,711 participants | European | 3.5 | 4,442 cases and 264,638 controls | OR per score unit | European | Two-sample | IVW | 0.94(0.79,1.13) | 0.520 | Meta-analysed UK-Biobank individual cancer estimates with those previously published using data from various studies and consortia | No | Unknown |
| Cornish AJ, 2019^77^ | Blood selenium | 1.000\|OR≤0.75 or OR≥1.33 | 2,603 participants | Queensland | 2 | 26,397 cases and 41,481 controls | OR per SD | European | Two-sample | Wald ratio | 0.85 (0.75-0.96) | 0.008** | Weighted median, mode-based estimates, MR-Egger, leave-one-out analysis | No | *Likely non-causal* |
| Cornish AJ, 2019^77^ | Blood zinc | 1.000\|OR≤0.75 or OR≥1.33 | 2,603 participants | Queensland | 4.6 | 26,397 cases and 41,481 controls | OR per SD | European | Two-sample | Maximum likelihood | 0.94 (0.86-1.03) | 0.179** | Weighted median, mode-based estimates, MR-Egger, leave-one-out analysis | No | Unknown |
| Cornish AJ, 2019^77^ | Circulating 25-hydroxyvitamin D | 1.000\|OR≤0.75 or OR≥1.33 | 79,366 participants | European | 2.6 | 26,397 cases and 41,481 controls | OR per SD | European | Two-sample | Maximum likelihood | 0.99 (0.90-1.09) | 0.895** | Weighted median, mode-based estimates, MR-Egger, leave-one-out analysis | No | Unknown |
| Cornish AJ, 2019^77^ | Circulating carotenoids | 1.000\|OR≤0.75 or OR≥1.33 | 1,190 participants | Italian | 2.8 | 26,397 cases and 41,481 controls | OR per SD | European | Two-sample | Wald ratio | 1.04 (0.94-1.15) | 0.451** | Weighted median, mode-based estimates, MR-Egger, leave-one-out analysis | No | Unknown |
| Cornish AJ, 2019^77^ | Iron status | 0.981\|OR≤0.75 or OR≥1.33 | 48,972 participants | European | 1.2 | 26,397 cases and 41,481 controls | OR per SD | European | Two-sample | Maximum likelihood | 1.17 (1.00-1.36) | 0.049** | Weighted median, mode-based estimates, MR-Egger, leave-one-out analysis | No | Unknown |
| Cornish AJ, 2019^77^ | Serum calcium | 1.000\|OR≤0.75 or OR≥1.33 | 39,400 participants | European | 2.6 | 26,397 cases and 41,481 controls | OR per SD | European | Two-sample | Maximum likelihood | 0.93 (0.83-1.05) | 0.264** | Weighted median, mode-based estimates, MR-Egger, leave-one-out analysis | No | Unknown |
| Cornish AJ, 2019^77^ | Serum vitamin A (retinol) | 0.879\|OR≤0.75 or OR≥1.33 | 5,006 participants | Finland, US | 0.7 | 26,397 cases and 41,481 controls | OR per SD | European | Two-sample | Maximum likelihood | 1.07 (0.78-1.47) | 0.663** | Weighted median, mode-based estimates, MR-Egger, leave-one-out analysis | No | Unknown |
| Cornish AJ, 2019^77^ | Serum vitamin B12 | 1.000\|OR≤0.75 or OR≥1.33 | 45,576 & 37,341 participants | Iceland, Denmark | 4.7 | 26,397 cases and 41,481 controls | OR per SD | European | Two-sample | Maximum likelihood | 1.21 (1.04-1.42) | 0.016** | Weighted median, mode-based estimates, MR-Egger, leave-one-out analysis | No | *Likely non-causal* |
| Cornish AJ, 2019^77^ | Serum vitamin B6 | 0.994\|OR≤0.75 or OR≥1.33 | 2,930 participants |  | 1.4 | 26,397 cases and 41,481 controls | OR per SD | European | Two-sample | Wald ratio | 1.04 (0.90-1.20) | 0.592** | Weighted median, mode-based estimates, MR-Egger, leave-one-out analysis | No | Unknown |
| Cornish AJ, 2019^77^ | Serum vitamin E | 0.857\|OR≤0.75 or OR≥1.33 | 5,006 participants | European | 0.7 | 26,397 cases and 41,481 controls | OR per SD | European | Two-sample | Maximum likelihood | 0.94 (0.76-1.17) | 0.600** | Weighted median, mode-based estimates, MR-Egger, leave-one-out analysis | No | Unknown |
| Fatty acid/Lipid metabolism biomarkers | | | | | | | | | | | | | | | |
| May-Wilson S, 2017^78^ | Plasma Arachidic acid (20:0) (GRS) | Limited power (not specified) | 38,000 participants | European | / | 9,254 cases and 18,386 controls | OR per SD | European | Two-sample | Meta-analysis of statistics for each specific fatty acid generated for each CRC cohort was combined under fixed-effects models | 0.92(0.61,1.39) | 0.700 | Where more than one instrument variant was available: heterogeneity assessment, random-effects inverse-variance weighted MR; assessing impact of pleiotropy by using IVW and MR-Egger methods | No | Unknown |
| May-Wilson S, 2017^78^ | Plasma Palmitic acid (16:0) (GRS) | Limited power (not specified) | 38,000 participants | European | 0.21-0.98 | 9,254 cases and 18,386 controls | OR per SD | European | Two-sample | Meta-analysis of statistics for each specific fatty acid generated for each CRC cohort was combined under fixed-effects models | 0.97(0.78,1.21) | 0.820 | Where more than one instrument variant was available: heterogeneity assessment, random-effects inverse-variance weighted MR; assessing impact of pleiotropy by using IVW and MR-Egger methods | No | Unknown |
| May-Wilson S, 2017^78^ | Plasma Stearic acid (18:0) (GRS) | Limited power (not specified) | 38,000 participants | European | 0.01-1.39 per SNP | 9,254 cases and 18,386 controls | OR per SD | European | Two-sample | Meta-analysis of statistics for each specific fatty acid generated for each CRC cohort was combined under fixed-effects models | 1.16(1.01,1.35) | 0.040 | Where more than one instrument variant was available: heterogeneity assessment, random-effects inverse-variance weighted MR; assessing impact of pleiotropy by using IVW and MR-Egger methods | No | Unknown |
| May-Wilson S, 2017^78^ | Plasma DHA (22:6n-3) (GRS) | Limited power (not specified) | 38,000 participants | European | 0.7 | 9,254 cases and 18,386 controls | OR per SD | European | Two-sample | Meta-analysis of statistics for each specific fatty acid generated for each CRC cohort was combined under fixed-effects models | 1.32(0.94,1.87) | 0.110 | Where more than one instrument variant was available: heterogeneity assessment, random-effects IVW MR; assessing impact of pleiotropy by using IVW and MR-Egger methods | No | Unknown |
| Liyanage UE, 2019^79^ | Plasma DHA 22:6n-3 | / | 8,866 participants | European | 0.65 | 4,545 cases and 270,342 controls combined 9,254 cases and 18,386 controls | OR per SD | European | Two-sample | Wald-type ratio estimator (combining estimate with those from May-Wilson et al 2017) | 1.07(0.84,1.36) | 0.583* | IVW estimate without combining with data from other study (May-Wilson et al 2017) | No | Unknown |
| May-Wilson S, 2017^78^ | Plasma DPA (22:5n-3) (GRS) | Limited power (not specified) | 38,000 participants | European | 2.8-8.6 per SNP | 9,254 cases and 18,386 controls | OR per SD | European | Two-sample | Meta-analysis of statistics for each specific fatty acid generated for each CRC cohort was combined under fixed-effects models | 1.58(0.99,2.52) | 0.060 | Where more than one instrument variant was available: heterogeneity assessment, random-effects inverse-variance weighted MR; assessing impact of pleiotropy by using IVW and MR-Egger methods | No | Unknown |
| Liyanage UE, 2019^79^ | Plasma EPA 20:5n-3 | / | 8,866 participants | European | 2.05 | 4,545 cases and 270,342 controls combined 9,254 cases and 18,386 controls | OR per SD | European | Two-sample | IVW (combining estimate with those from May-Wilson et al 2017) | 1.06(0.91,1.22) | 0.455* | IVW estimate without combining with data from other study (May-Wilson et al 2017) | No | Unknown |
| May-Wilson S, 2017^78^ | Plasma EPA (20:5n-3) (GRS) | <0.1 | 38,000 participants | European | 0.4 | 9,254 cases and 18,386 controls | OR per SD | European | Two-sample | Meta-analysis of statistics for each specific fatty acid generated for each CRC cohort was combined under fixed-effects models | 0.39(0.13,1.21) | 0.100 | Where more than one instrument variant was available: heterogeneity assessment, random-effects inverse-variance weighted MR; assessing impact of pleiotropy by using IVW and MR-Egger methods | No | Unknown |
| Liyanage UE, 2019^79^ | Plasma DPA 20:5n-3 | / | 8,866 participants | European | 11.12 | 4,545 cases and 270,342 controls combined 9,254 cases and 18,386 controls | OR per SD | European | Two-sample | IVW (combining estimate with those from May-Wilson et al 2017) | 1.10(1.01,1.19) | 0.034* | IVW estimate without combining with data from other study (May-Wilson et al 2017) | No | Unknown |
| May-Wilson S, 2017^78^ | Plasma AA (20:4n-6) (GRS) | Limited power (not specified) | 38,000 participants | European | 0.1-37.6 per SNP | 9,254 cases and 18,386 controls | OR per SD | European | Two-sample | Meta-analysis of statistics for each specific fatty acid generated for each CRC cohort was combined under fixed-effects models | 1.05(1.02,1.07) | 1.70E-04 | Where more than one instrument variant was available: heterogeneity assessment, random-effects inverse-variance weighted MR; assessing impact of pleiotropy by using IVW and MR-Egger methods | Yes | Evidence of causality |
| Liyanage UE, 2019^79^ | Plasma AA 20:4n-6 | / | 8,631 participants | White adults | 33.07 | 4,545 cases and 270,342 controls combined 9,254 cases and 18,386 controls | OR per SD | European | Two-sample | IVW (combining estimate with those from May-Wilson et al 2017) | 1.05(1.03,1.07) | 2.00E-05* | IVW estimate without combining with data from other study (May-Wilson et al 2017) | No | Unknown |
| May-Wilson S, 2017^78^ | Plasma DGLA (20:3n-6) (GRS) | Limited power (not specified) | 38,000 participants | European | 2-11.1 per SNP | 9,254 cases and 18,386 controls | OR per SD | European | Two-sample | Meta-analysis of statistics for each specific fatty acid generated for each CRC cohort was combined under fixed-effects models | 0.91(0.83,1.00) | 0.060 | Where more than one instrument variant was available: heterogeneity assessment, random-effects inverse-variance weighted MR; assessing impact of pleiotropy by using IVW and MR-Egger methods | No | Unknown |
| May-Wilson S, 2017^78^ | Plasma LA (18:2n-6) (GRS) | Limited power (not specified) | 38,000 participants | European | 0.2-18.1 per SNP | 9,254 cases and 18,386 controls | OR per SD | European | Two-sample | Meta-analysis of statistics for each specific fatty acid generated for each CRC cohort was combined under fixed-effects models | 0.95(0.93,0.98) | 3.70E-04 | Where more than one instrument variant was available: heterogeneity assessment, random-effects IVW MR; assessing impact of pleiotropy by using IVW and MR-Egger methods | Yes | Evidence of causality |
| Liyanage UE, 2019^79^ | Plasma LA 18:2n-6 | / | 8,631 participants | White adults | 8.3 | 4,545 cases and 270,342 controls combined 9,254 cases and 18,386 controls | OR per SD | European | Two-sample | IVW (combining estimate with those from May-Wilson et al 2017) | 0.95(0.93,0.97) | 9.60E-05* | IVW estimate without combining with other study | No | Unknown |
| May-Wilson S, 2017^78^ | Plasma Oleic acid (18:1n-9) (GRS) | Limited power (not specified) | 38,000 participants | European | 0.32-2.14 per SNP | 9,254 cases and 18,386 controls | OR per SD | European | Two-sample | Meta-analysis of statistics for each specific fatty acid generated for each CRC cohort was combined under fixed-effects models | 0.77(0.65,0.92) | 0.004 | Where more than one instrument variant was available: heterogeneity assessment, random-effects IVW MR; assessing impact of pleiotropy by using IVW and MR-Egger methods | Yes | Evidence of causality |
| May-Wilson S, 2017^78^ | Plasma Palmitoleic acid (16:1n-7) (GRS) | Limited power (not specified) | 38,000 participants | European | 0.01-1.57 per SNP | 9,254 cases and 18,386 controls | OR per SD | European | Two-sample | Meta-analysis of statistics for each specific fatty acid generated for each CRC cohort was combined under fixed-effects models | 0.36(0.15,0.84) | 0.018 | Where more than one instrument variant was available: heterogeneity assessment, random-effects IVW MR; assessing impact of pleiotropy by using IVW and MR-Egger methods | No | Unknown |
| Liyanage UE, 2019^79^ | Plasma ALA 18:3n-3 | / | 8,866 participants | European | 1.03 | 4,545 cases and 270,342 controls combined 9,254 cases and 18,386 controls | OR per SD | European | Two-sample | Wald-type ratio estimator (combining estimate with those from May-Wilson et al 2017) | 0.89(0.78,1.02) | 0.098* | IVW estimate without combining with other study | No | Unknown |
| Rodriguez-Broadbent H, 2017^80^ | Total cholesterol (GRS) | / | 188,577 participants | European | 8-11 per SNP | 9,254 cases and 18,386 controls | OR per SD | European | Two-sample | IVW | 1.46(1.20,1.79) | 1.68E-04 | Restricted allele score based on SNPs exclusively associated with total cholesterol; LD regression; MR-Egger; omission two strongest SNPs | Yes | Evidence of causality |
| Rodriguez-Broadbent H, 2017^80^ | Triglyceride (GRS) | 0.13 | 188,577 participants | European | 8-11 per SNP | 9,254 cases and 18,386 controls | OR per SD | European | Two-sample | IVW | 0.98(0.85,1.12) | 0.752 | Restricted allele score based on SNPs exclusively associated with triglyceride; LD regression; MR-Egger | No | Unknown |
| Orho-Melander M, 2018^81^ | Triglyceride (GRS) | 0.8\|OR≥1.77 | 96,598 participants | European | 4.9 | 497 cases, 26,904 participants | OR per SD | Swedish | Two-sample | Logistic regression | 1.16(0.78,1.74) | / | Multivariable MR analysis adjusting for the three lipid traits. | No | Unknown |
| Rodriguez-Broadbent H, 2017^80^ | LDL (GRS) | 0.68 | 188,577 participants | European | 8-11 per SNP | 9,254 cases and 18,386 controls | OR per SD | European | Two-sample | IVW | 1.05(0.92,1.18) | 0.485 | Restricted allele score based on SNPs exclusively associated with LDL; LD regression; MR-Egger | No | Unknown |
| Orho-Melander M, 2018^81^ | LDL (GRS) | 0.8\|OR≥1.61 | 95,454 participants | European | 7.1 | 497 cases, 26,904 participants | OR per SD | Swedish | Two-sample | Logistic regression | 1.18(0.85,1.65) | / | Multivariable MR analysis adjusting for the three lipid traits. | No | Unknown |
| Orho-Melander M, 2018^81^ | HDL (GRS) | 0.8\|OR≥1.68 | 99,900 participants | European | 6 | 497 cases, 26,904 participants | OR per SD | Swedish | Two-sample | Logistic regression | 0.92(0.65,1.33) | / | Multivariable MR analysis adjusting for the three lipid traits. | No | Unknown |
| Rodriguez-Broadbent H, 2017^80^ | HDL (GRS) | 0.31 | 188,577 participants | European | 8-11 per SNP | 9,254 cases and 18,386 controls | OR per SD | European | Two-sample | IVW | 0.94(0.84,1.05) | 0.273 | Restricted allele score based on SNPs exclusively associated with HDL; LD regression; MR-Egger | No | Unknown |
| Cornish AJ, 2019^77^ | HDL | 1.000\|OR≤0.75 or OR≥1.33 | 188,577 participants | European | 6.1 | 26,397 cases and 41,481 controls | OR per SD | European | Two-sample | Maximum likelihood | 1.03 (0.92-1.14) | 0.620** | Weighted median, mode-based estimates, MR-Egger, leave-one-out analysis | No | Unknown |
| Cornish AJ, 2019^77^ | LDL | 1.000\|OR≤0.75 or OR≥1.33 | 188,577 participants | European | 7.9 | 26,397 cases and 41,481 controls | OR per SD | European | Two-sample | Maximum likelihood | 1.14 (1.04-1.25) | 0.006** | Weighted median, mode-based estimates, MR-Egger, leave-one-out analysis | No | *Likely non-causal* |
| Cornish AJ, 2019^77^ | Mono-unsaturated fatty acids | 0.493\|OR≤0.75 or OR≥1.33 | 24,925 participants | European | 0.3 | 26,397 cases and 41,481 controls | OR per SD | European | Two-sample | Wald ratio | 1.07 (0.78-1.46) | 0.672** | Weighted median, mode-based estimates, MR-Egger, leave-one-out analysis | No | Unknown |
| Cornish AJ, 2019^77^ | Omega-6 polyunsaturated fatty acids | 1.000\|OR≤0.75 or OR≥1.33 | 24,925 participants | European | 2.4 | 26,397 cases and 41,481 controls | OR per SD | European | Two-sample | Maximum likelihood | 1.15 (0.98-1.36) | 0.095** | Weighted median, mode-based estimates, MR-Egger, leave-one-out analysis | No | Likely non-causal |
| Cornish AJ, 2019^77^ | Total cholesterol | 1.000\|OR≤0.75 or OR≥1.33 | 24,925 participants | European | 9.5 | 26,397 cases and 41,481 controls | OR per SD | European | Two-sample | Maximum likelihood | 1.09 (1.01-1.18) | 0.025** | Weighted median, mode-based estimates, MR-Egger, leave-one-out analysis | No | *Likely non-causal* |
| Cornish AJ, 2019^77^ | Total triglycerides | 1.000\|OR≤0.75 or OR≥1.33 | 188,577 participants | European | 6.1 | 26,397 cases and 41,481 controls | OR per SD | European | Two-sample | Maximum likelihood | 0.93 (0.84-1.04) | 0.192** | Weighted median, mode-based estimates, MR-Egger, leave-one-out analysis | No | Unknown |
| Inflammatory Markers | | | | | | | | | | | | | | | |
| Nimptsch K, 2015^82^ | C-reactive protein (Unweighted GRS) | / | 727 participants | European | 2 to 3 | 727 cases and 727 controls | OR per 2-fold higher | European | One-sample | Conditional logistic regression | 1.74(1.06,2.85) | / | Investigation for sex-specific associations; adjustments for observationally measured smoking, education, alcohol consumption, dietary intake and physical activity inﬂuenced the risk estimates; probit regression models, analysis of individual single-nucleotide polymorphisms; using weighted GRS or Haplotype frequency as instrumental variable | No | Unknown |
| Wang X, 2018^83^ | C-reactive protein (19 SNPs) | 0.825\|OR≥1.12 | 66,185 & 40,473 participants | European | 5 | 30,480 cases and 22,844 controls | OR per SD | European | Two-sample | IVW | 1.04(0.97,1.12) | 0.256 | Subgroup analyses stratified by cancer sites and stages, sex, BMI, smoking, NSAID use, aspirin use, history of endoscopy, family history of CRC; Egger regression | No | Unknown |
| Cornish AJ, 2019^77^ | Circulating C-reactive protein | 1.000\|OR≤0.75 or OR≥1.33 | 66,185 participants | European | 3.6 | 26,397 cases and 41,481 controls | OR per SD | European | Two-sample | Maximum likelihood | 0.95 (0.83-1.10) | 0.527** | Weighted median, mode-based estimates, MR-Egger, leave-one-out analysis | No | Unknown |
| Cornish AJ, 2019^77^ | Plasma IL-6 receptor subunit alpha | 1.000\|OR≤0.75 or OR≥1.33 | 3,301 participants | European | 60.4 | 26,397 cases and 41,481 controls | OR per SD | European | Two-sample | Wald ratio | 0.98 (0.96-1.00) | 0.035** | Weighted median, mode-based estimates, MR-Egger, leave-one-out analysis | No | Unknown |
| Insulin related markers | | | | | | | | | | | | | | | |
| Cornish AJ, 2019^77^ | Fasting glucose | 1.000\|OR≤0.75 or OR≥1.33 | 96,496 participants | European | 3.6 | 26,397 cases and 41,481 controls | OR per SD | European | Two-sample | Maximum likelihood | 1.04 (0.92-1.18) | 0.519** | Weighted median, mode-based estimates, MR-Egger, leave-one-out analysis | No | Unknown |
| Cornish AJ, 2019^77^ | Fasting proinsulin | 1.000\|OR≤0.75 or OR≥1.33 | 46,186 participants | European | 6.1 | 26,397 cases and 41,481 controls | OR per SD | European | Two-sample | Maximum likelihood | 0.97 (0.90-1.03) | 0.310** | Weighted median, mode-based estimates, MR-Egger, leave-one-out analysis | No | Unknown |
| Cornish AJ, 2019^77^ | HbA1C levels | 0.999\|OR≤0.75 or OR≥1.33 | 46,368 participants | European | 1.8 | 26,397 cases and 41,481 controls | OR per SD | European | Two-sample | Maximum likelihood | 1.02 (0.85-1.22) | 0.866** | Weighted median, mode-based estimates, MR-Egger, leave-one-out analysis | No | Unknown |
| Cornish AJ, 2019^77^ | Plasma IGF−I | 0.995\|OR≤0.75 or OR≥1.33 | 3301 participants | European | 1.4 | 26,397 cases and 41,481 controls | OR per SD | European | Two-sample | Wald ratio | 0.88 (0.76-1.01) | 0.064** | Weighted median, mode-based estimates, MR-Egger, leave-one-out analysis | No | Unknown |
| Other Biomarkers | | | | | | | | | | | | | | | |
| Zhang C, 2015^84^ | Telomere length (GRS) | 0.8\|OR≥1.22 | 37,684 &9,190 & 2,240 participants | European | 0.06-0.2 per SNP | 5,100 cases and 4,831 controls | OR per 1000 base pair increase | European | Two-sample | IVW | 1.25(0.92,1.69) | 0.150 | Stratified by age and sex; likelihood-based Mendelian randomization method; Alternative instrument selection strategy | No | Likely non-causal |
| Haycock PC, 2017^85^ | Telomere length | 1 | 9,190 participants | European | / | 14,537 cases and 16,922 controls | OR per SD | European | Two-sample | Maximum likelihood | 1.09(0.91,1.31) | 0.340 | Heterogeneity analysis, weighted median and MR-Egger | No | Likely non-causal |
| Protein& amino acid | | | | | | | | | | | | | | | |
| Au Yeung SL, 2019^86^ | GDF-15 | 0.8\|OR≥1.11 (R2=0.15) or OR≥1.10 (R2=0.21) | 5,440 participants | European | 15 or 21 | 4,562 cases and 382,756 controls | OR per SD | European | Two-sample | IVW (Fixed effect) | 0.91(0.80,1.04) | / | Multiplicative random effect IVW; Lead SNP analysis; MR-Egger intercept test; Mendelian randomisation restricted to instruments from the same gene region (PGPEP1 or GDF15) | No | Unknown |
| Nimptsch K, 2017^87^ | Adiponectin (ADIPOQ allele score) | 0.8\|OR≤0.61 | 2,880 participants | European and US | 3 | 1,253 cases and 1,627 controls | OR per score unit | European and US | One-sample | Conditional logistic regression | 0.97(0.91,1.04) | 0.430 | Restriction to Caucasians; Effect estimate in different study (HPFS and NHS); summary instrumental variable analysis using published data on genetic associations with adiponectin and colorectal cancer in a likelihood-based approach | No | Unknown |
| Nimptsch K, 2017^87^ | Adiponectin (Incorporating the ADIPOQ allele score and plasma adiponectin concentrations) | 0.8\|OR≤0.76 | 2,880 participants | European and US | / | 1,253 cases and 1,627 controls | OR per score unit | European and US | One-sample | Conditional logistic regression | 0.73(0.40,1.34) | / | Restriction to Caucasians; Effect estimate in different study (HPFS and NHS) | No | Likely non-causal |
| Nimptsch K, 2015^88^ | Fetuin-A (AHSG allele-score) | Limited power (not specified) | 1,367 cases and 1,367 controls | Western European | 21 | 1,367 cases and 1,367 controls | RR per 40mg/ml higher (aapprox 1 SD) | Western European | One-sample | Logistic regression | 0.98(0.73,1.33) | / | Conditional logistic regression models additionally adjusted for matching factors and BMI | No | Unknown |
| Cornish AJ, 2019^77^ | Blood carnitine | 1.000\|OR≤0.75 or OR≥1.33 | 7,824 participants | European | 13.9 | 26,397 cases and 41,481 controls | OR per SD | European | Two-sample | Maximum likelihood | 0.99 (0.92-1.06) | 0.682** | Weighted median, mode-based estimates, MR-Egger, leave-one-out analysis | No | Unknown |
| Cornish AJ, 2019^77^ | Blood methionine | 0.676\|OR≤0.75 or OR≥1.33 | 7,824 participants | European | 0.4 | 26,397 cases and 41,481 controls | OR per SD | European | Two-sample | Wald ratio | 0.92 (0.70-1.19) | 0.505** | Weighted median, mode-based estimates, MR-Egger, leave-one-out analysis | No | Unknown |
| Cornish AJ, 2019^77^ | Circulating adiponectin | 1.000\|OR≤0.75 or OR≥1.33 | 39,883 participants | European | 1.8 | 26,397 cases and 41,481 controls | OR per SD | European | Two-sample | Maximum likelihood | 0.93 (0.81-1.07) | 0.309** | Weighted median, mode-based estimates, MR-Egger, leave-one-out analysis | No | Unknown |
| Cornish AJ, 2019^77^ | Circulating fetuin-A | 1.000\|OR≤0.75 or OR≥1.33 | 9,055 & 2,119 participants | European, African American | 14.3 | 26,397 cases and 41,481 controls | OR per SD | European | Two-sample | Wald ratio | 0.98 (0.94-1.02) | 0.370** | Weighted median, mode-based estimates, MR-Egger, leave-one-out analysis | No | Unknown |
| Cornish AJ, 2019^77^ | Serum immunoglobulin E | 0.997\|OR≤0.75 or OR≥1.33 | 6,819 participants | European | 1.6 | 26,397 cases and 41,481 controls | OR per SD | European | Two-sample | Maximum likelihood | 0.92 (0.82-1.03) | 0.159** | Weighted median, mode-based estimates, MR-Egger, leave-one-out analysis | No | Unknown |
| *: statistically significant threshold set up at P≤0.0001; **: statistically significant threshold set up at P≤0.0013, GI: genetic instrument, SNP: Single Nucleotide Polymorphism, OR: odds ratio, HR: hazard ratio, SD: standard deviation, GRS: genetic risk score, BMI: body mass index, IVW: inverse variance weighted, EPA: Eicosapentaenoic acid, DHA: Docosahexaenoic acid, DPA: Docosapentaenoic acid, AA: arachidonic acid, DGLA: dihomo-γ-linolenic acid, LA: linoleic acid, ALA: α-Linolenic acid, HDL: high-density lipoprotein cholesterol, LD: linkage disequilibrium, LDL: low-density lipoprotein cholesterol, IL-6: interleukin 6, HbA1C: glycated hemoglobin, IGF-1: insulin-like growth factor 1, GDF-15: Growth differentiation factor 15. | | | | | | | | | | | | | | | |

| **Supplementary Table 6 Summary of overlapped meta-analyses of observational studies and Mendelian randomization studies** | | | | |
| --- | --- | --- | --- | --- |
| **Overlapped results from meta-analyses of observational studies** | | | | |
| Biomarker | No. of overlapping studies | Agreement of Direction of Point Estimate | Agreement of Presence of Nominal Significance (p<0.05) | Reference |
| H. pylori infection | 9 | Y | N | ^6-12,20^ |
| HPV | 8 | Y | Y | ^13-19^ |
| Vitamin B9 [Folate] | 6 | N | N | ^35-38^ |
| Vitamin B12 | 5 | N | N | ^36,38,39^ |
| Vitamin B6 | 5 | Y | Y | ^36,41,42^ |
| Vitamin B2 | 2 | Y | N | ^36,40^ |
| 25-hydroxyvitamin D | 10 | Y | Y | ^45-51^ |
| CRP | 3 | Y | Y | ^23-25^ |
| IL-6 | 2 | Y | NON | ^24,26^ |
| Fasting Glucose | 6 | Y | Y | ^27,28,30,31^ |
| C peptide | 3 | Y | Y | ^27-29^ |
| IGF 1 | 3 | Y | Y | ^32,33^ |
| IGF 2 | 2 | Y | Y | ^32,34^ |
| IGFBP 3 | 2 | Y | NON | ^32,89^ |
| Triglycerides | 3 | Y | N | ^2,3^ |
| HDL–cholesterol | 2 | Y | Y | ^2,3^ |
| Adiponectin | 7 | N | N | ^59-63,90^ |
| Leptin | 4 | Y | N | ^62,66,90^ |
| Homocysteine | 3 | Y | Y | ^36,38^ |
| Telomere Length | 3 | Y | N | ^57,58^ |
| **Overlapped results from Mendelian randomisation studies** | | | | |
| 25-hydroxyvitamin D | 8 | N | NON | ^72-77^ |
| Adiponectin | 3 | Y | NON | ^77,87,91^ |
| Fetuin-A | 2 | Y | NON | ^77,88^ |
| DHA 22:6n-3 | 2 | Y | NON | ^78,79^ |
| DPA 20:5n-3 | 2 | Y | NON | ^78,79^ |
| EPA 20:5n-3 | 2 | N | NON | ^78,79^ |
| AA 20:4n-6 | 2 | Y | Y | ^78,79^ |
| LA 18:2n-6 | 2 | Y | Y | ^78,79^ |
| Triglyceride | 3 | N | NON | ^77,80,81^ |
| LDL–cholesterol | 3 | Y | NON | ^80,81^ |
| HDL–cholesterol | 3 | N | NON | ^80,81^ |
| Total cholesterol | 2 | Y | N | ^77,80^ |
| C-reactive protein | 3 | N | N | ^82,83^ |
| Telomere length | 2 | Y | NON | ^84,85^ |
| **Comparison between the largest meta-analyses of observational studies and the largest Mendelian randomisation studies** | | | | |
| 25-hydroxyvitamin D | / | Y | N | ^49,72^ |
| Selenium | / | Y | NON | ^44,77^ |
| Vitamin B12 | / | N | N | ^36,77^ |
| Vitamin B6 | / | N | N | ^41,77^ |
| Vitamin E | / | Y | NON | ^53,77^ |
| Zinc | / | N | NON | ^54,77^ |
| DHA 22:6n-3 | / | N | N | ^1,78^ |
| EPA 20:5n-3 | / | N | N | ^1,78^ |
| DPA 20:5n-3 | / | N | NON | ^1,78^ |
| Total cholesterol | / | Y | NON | ^3,80^ |
| Triglyceride | / | N | NON | ^3,80^ |
| LDL–cholesterol | / | Y | NON | ^3,80^ |
| HDL–cholesterol | / | N | NON | ^3,80^ |
| C-reactive protein | / | Y | N | ^24,83^ |
| IL-6 | / | N | NON | ^24,77^ |
| Fasting glucose | / | Y | N | ^27,77^ |
| HbA1C | / | Y | NON | ^27,77^ |
| IGF−I | / | N | N | ^32,77^ |
| Adiponectin | / | Y | N | ^59,77^ |
| Telomere length | / | Y | NON | ^58,85^ |
| Y: agree, N: disagree, NON: non-significant association has been identified, CRC: colorectal cancer, H. pylori: helicobacter pylori, HPV: human papillomavirus infection, CRP: C-reactive protein, IL-6: Interleukin 6, IGF 1/2: Insulin-like growth factor 1/2, IGFBP 3: Insulin-like growth factor-binding protein 3, HDL: high-density lipoprotein cholesterol, LDL: low-density lipoprotein cholesterol, DHA: Docosahexaenoic acid, DPA: Docosapentaenoic acid, EPA: Eicosapentaenoic acid, AA: arachidonic acid, LA: linoleic acid, HbA1c: glycated hemoglobin | | | | |

| **Supplementary Table 7 Characteristics and main findings of meta-analyses of observational studies reporting unique non-genetic biomarkers and CRC risk (DL, PM, HKSJ estimators)** | | | | | | | | | | | |
| --- | --- | --- | --- | --- | --- | --- | --- | --- | --- | --- | --- |
| Author/Year | Biomarker | No of studies | No of cases | No of participants | Metric | Effect size, DL (95%CI) | P, DL | I^2^, DL | P_Egger_, DL | 95%PI, DL | Effect size, PM (95%CI) |
| **Fatty acid/Lipid metabolism biomarkers** | | | | | | | | | | | |
| Yang B,2014 | LC n-3 PUFA | 3 | 421 | 1,826 | RR | 0.58(0.40,0.84) | 0.0042 | 0.00% | 0.78 | 0.58(0.40,0.84) | 0.58(0.40,0.84) |
| Yang B,2014 | Biospecimen EPA 20:5 (n-3) | 4 | 421 | 1,826 | RR | 0.64(0.43,0.94) | 0.0242 | 0.00% | 0.48 | 0.64(0.43,0.94) | 0.64(0.43,0.94) |
| Yang B,2014 | Biospecimen DHA 22:6 (n-3) | 4 | 421 | 1,826 | RR | 0.53(0.32,0.87) | 0.0124 | 27.00% | 0.12 | 0.53(0.32,0.87) | 0.53(0.32,0.88) |
| Yang B,2014 | Biospecimen DPA 22:5 (n-3) | 3 | 243 | 1,366 | RR | 0.57(0.33,0.98) | 0.0433 | 15.54% | 0.44 | 0.57(0.33,0.98) | 0.57(0.32,0.99) |
| Yang B,2014 | Blood EPA 20:5 (n-3) | 5 | 195 | 384 | SMD | -0.76(-1.55,0.03) | 0.0603 | 93.24% | 0.00 | -0.76(-1.56,0.03) | -0.99(-2.89,0.92) |
| Yang B,2014 | Blood DHA 22:6 (n-3) | 5 | 195 | 384 | SMD | -0.09(-0.27,0.09) | 0.3098 | 0.00% | 0.31 | -0.09(-0.27,0.09) | -0.10(-0.27,0.09) |
| Yang B,2014 | Blood DPA 22:5 (n-3) | 5 | 195 | 384 | SMD | 0.36(-0.10,0.83) | 0.1245 | 81.83% | 0.00 | 0.36(-0.10,0.83) | 0.43(-0.27,1.12) |
| Yang B,2014 | Adipose EPA 20:5 (n-3) | 2 | 93 | 204 | SMD | 0.07(-0.20,0.35) | 0.6024 | 0.00% | / | 0.07(-0.20,0.35) | 0.08(-0.20,0.35) |
| Yang B,2014 | Adipose DHA 22:6 (n-3) | 2 | 93 | 204 | SMD | -0.02(-0.35,0.30) | 0.892 | 26.74% | / | -0.02(-0.35,0.30) | -0.02(-0.35,0.30) |
| Yang B,2014 | Adipose DPA 22:5 (n-3) | 2 | 93 | 204 | SMD | 0.12(-0.16,0.39) | 0.4081 | 0.00% | / | 0.12(-0.16,0.39) | 0.12(-0.16,0.39) |
| Yao X,2015 | HDL | 12 | 2,542 | 136,698 | RR | 0.84(0.69,1.02) | 0.0768 | 42.55% | 0.39 | 0.84(0.69,1.02) | 0.84(0.69,1.02) |
| Yao X,2015 | LDL | 5 | 1,626 | 9,175 | RR | 1.04(0.60,1.81) | 0.8906 | 82.66% | 0.13 | 1.04(0.60,1.81) | 1.04(0.57,1.92) |
| Yao X,2015 | Total cholesterol | 28 | 10,892 | 7,725,310 | RR | 1.11(1.01,1.21) | 0.0294 | 46.75% | 0.54 | 1.11(1.01,1.21) | 1.11(0.99,1.24) |
| Yao X,2015 | Triglyceride | 19 | 8,127 | 2,252,217 | RR | 1.18(1.04, 1.34) | 0.012 | 47.77% | 0.20 | 1.18(1.04,1.33) | 1.17(1.02,1.35) |
| **Infectious agents** | | | | | | | | | | | |
| Ibragimova MK,2018 | HPV | 17 | 1,722 | 2,468 | RR | 2.69(1.86,3.90) | 1.61E-07 | 25.50% | 0.10 | 2.70(1.86,3.90) | 2.93(1.87,4.57) |
| Bai B,2016 | Human cytomegalovirus infection | 4 | 480 | 960 | OR | 6.47(4.40,9.52) | 2.91E-21 | 0.00% | 0.73 | 6.47(4.40,9.52) | 6.47(4.40,9.52) |
| Boleij A,2011 | Streptococcus bovis | 6 | 189 | 340 | OR | 9.68(4.99,18.81) | 2.03E-11 | 0.00% | 0.51 | 9.68(4.99,18.81) | 9.68(4.99,18.81) |
| Liu C,2016 | H.pylori | 20 | 3,228 | 4,377 | OR | 1.37(1.10,1.71) | 0.0048 | 61.09% | 0.78 | 1.37(1.10,1.71) | 1.38(1.04,1.84) |
| Repass J,2018 | F. nucleatum | 2 | 139 | 278 | r | 0.40(0.17,0.62) | 0.0005 | 34.95% | / | 0.38(0.17,0.55) | 0.40(0.17,0.62) |
| Liu H,2016 | Enterobacteriaceae | 2 | 37 | 76 | SMD | 2.62(0.55,4.69) | 0.0133 | 88.74% | / | 2.62(0.55,4.69) | 2.62(0.55,4.69) |
| Liu H,2016 | Bifidobacterium | 4 | 127 | 315 | SMD | -3.24(-6.47, -0.02) | 0.0486 | 98.25% | 0.06 | -3.24(-6.47, -0.02) | -3.24(-6.33, -0.15) |
| Liu H,2016 | Faecalibacterium prausnitzii | 3 | 86 | 233 | SMD | -0.32(-0.59, -0.05) | 0.0192 | 0.00% | 0.63 | -0.32(-0.59, -0.05) | -0.32(-0.59, -0.05) |
| Liu H,2016 | Total bacteria | 3 | 86 | 233 | SMD | 0.21(-0.53,0.96) | 0.5758 | 75.81% | 0.05 | 0.21(-0.53,0.96) | 0.29(-0.75,1.32) |
| Krishnan S,2014 | Streptococcus bovis in faeces | 3 | 148 | 414 | OR | 2.84(0.48,16.74) | 0.2483 | 66.82% | 0.18 | 2.84(0.48,16.74) | 3.10(0.68,14.20) |
| Liu H,2016 | Lactobacillus | 4 | 127 | 315 | SMD | -1.78(-3.76, 0.20) | 0.0787 | 97.31% | 0.03 | -1.78(-3.76,0.20) | -1.85(-5.35,1.66) |
| Liu H,2016 | Bacteroides-Prevotella group | 3 | 97 | 255 | SMD | -0.70(-2.53,1.14) | 0.4552 | 96.27% | 0.84 | -0.70(-2.53,1.14) | -0.70(-2.35,0.95) |
| Liu H,2016 | Escherichia coli | 2 | 90 | 239 | SMD | 1.38(-1.36,4.12) | 0.3239 | 98.00% | / | 1.38(-1.36,4.12) | 1.38(-1.36,4.12) |
| **Inflammatory Markers** | | | | | | | | | | | |
| Zhou B,2014 | CRP | 18 | 4,779 | 152,942 | RR | 1.12(1.05,1.21) | 0.0013 | 51.74% | 0.02 | 1.12(1.05,1.20) | 1.13(1.04,1.23) |
| Zhou B,2014 | IL-6 | 6 | 1,125 | 9,909 | RR | 1.10(0.88,1.36) | 0.4012 | 34.82% | 0.11 | 1.10(0.88,1.36) | 1.10(0.88,1.36) |
| **Insulin related biomarkers** | | | | | | | | | | | |
| Xu J,2016 | Fasting glucose | 26 | 20,390 | 5,105,567 | RR | 1.23(1.11,1.36) | 8.92E-05 | 40.90% | 0.00 | 1.23(1.11,1.36) | 1.23(1.11,1.36) |
| Xu J,2016 | HOMA-IR | 9 | 2,956 | 18,358 | RR | 1.54(1.23,1.92) | 0.0001 | 30.60% | 0.12 | 1.54(1.23,1.92) | 1.52(1.24,1.86) |
| Xu J,2016 | Fasting insulin | 11 | 3,191 | 26,301 | OR | 1.15(1.19,1.69) | 1.28E-04 | 0.00% | 0.70 | 1.42(1.19,1.69) | 1.42(1.19,1.69) |
| Chi F,2013 | IGF 1 | 17 | 3,807 | 11,613 | OR | 1.28(1.07,1.53) | 0.0061 | 20.62% | 0.17 | 1.28(1.07,1.53) | 1.28(1.07,1.54) |
| Chi F,2013 | IGF 2 | 6 | 783 | 4,007 | OR | 1.52(1.09,2.11) | 0.0134 | 14.94% | 0.64 | 1.52(1.09,2.11) | 1.52(1.08,2.12) |
| Chi F,2013 | IGFBP 1 | 7 | 2,154 | 6,439 | OR | 0.85(0.69,1.04) | 0.1043 | 3.68% | 0.26 | 0.85(0.69,1.04) | 0.84(0.69,1.04) |
| Chi F,2013 | IGFBP 2 | 3 | 1,348 | 2,962 | OR | 0.77(0.41,1.43) | 0.4006 | 68.21% | 0.01 | 0.77(0.41,1.43) | 0.77(0.41,1.43) |
| Chi F,2013 | IGFBP 3 | 16 | 3,755 | 11,509 | OR | 0.88(0.71,1.10) | 0.2680 | 45.74% | 0.31 | 0.88(0.71,1.10) | 0.88(0.69,1.11) |
| Xu J,2016 | HbA1c | 8 | 2,137 | 45,569 | RR | 1.23(0.98,1.54) | 0.0719 | 24.77% | 0.31 | 1.23(0.98,1.54) | 1.23(0.97,1.55) |
| Xu J,2016 | C-peptide | 11 | 3,211 | 13,888 | RR | 1.32(1.02,1.70) | 0.0325 | 48.09% | 0.50 | 1.32(1.02,1.70) | 1.34(0.99,1.79) |
| **Micronutrients** | | | | | | | | | | | |
| Ma YL,2011 | 25-hydroxyvitamin D | 10 | 3,142 | 7,840 | RR | 0.68(0.57,0.81) | 2.87E-05 | 0.00% | 0.61 | 0.68(0.57,0.82) | 0.68(0.57,0.81) |
| Lee JE,2011 | 1,25-dihydroxyvitamin D | 4 | 625 | 1,801 | OR | 1.02(0.66,1.56) | 0.9357 | 39.13% | 0.63 | 1.02(0.66,1.56) | 1.01(0.67,1.53) |
| Ben S, 2018 | Vitamin B2 | 2 | 1,593 | 32,962 | RR | 0.73(0.59,0.92) | 0.0069 | 0.00% | / | 0.74(0.59,0.92) | 0.73(0.59,0.92) |
| Larsson SC,2010 | Vitamin B6 | 4 | 883 | 2,207 | RR | 0.52(0.38,0.71) | 4.39E-05 | 0.00% | 0.97 | 0.52(0.38,0.71) | 0.52(0.38,0.71) |
| S. Shiao SPK,2018 | Vitamin B12 | 8 | 3,296 | 8,290 | SMD | -0.07(-0.13, -0.003) | 0.0378 | 52.44% | 0.00 | -0.07(-0.19,0.05) | -0.05(-0.12,-0.002) |
| Zhang D, 2015 | Folate | 12 | 1,159 | 2,982 | SMD | -1.01(-1.52, -0.51) | 7.66E-05 | 98.01% | 0.00 | -1.01(-1.93, -0.10) | -1.29(-2.27, -0.31) |
| Dong YH,2017 | Vitamin E | 9 | 310 | 5,927 | SMD | -0.74(-1.30, -0.17) | 0.0108 | 92.33% | 0.00 | -0.74(-1.31, -0.17) | -0.79(-1.63,0.05) |
| Vinceti M,2018 | Selenium | 8 | 2,627 | 712,746 | OR | 0.86(0.70,1.06) | 0.1585 | 0.00% | 0.84 | 0.86(0.70,1.06) | 0.86(0.70,1.06) |
| Gumulec J,2014 | Serum Zinc | 5 | 313 | 529 | SMD | 0.05(-2.55,2.64) | 0.9725 | 98.98% | 0.66 | 0.05(-2.55,2.64) | 0.05(-3.47,3.56) |
| Gumulec J,2014 | Tissue Zinc | 10 | 234 | 398 | SMD | -0.24(-1.50,1.02) | 0.7088 | 95.34% | 0.00 | -0.24(-1.50,1.02) | -0.70(-2.84,1.45) |
| **Other Biomarkers** | | | | | | | | | | | |
| Zhang BL,2015 | Blood group O | 8 | 6,931 | 3,219,151 | OR | 0.90(0.86,0.95) | 5.78E-05 | 0.00% | 0.22 | 0.90(0.86,0.95) | 0.90(0.86,0.95) |
| Zhang BL,2015 | Blood group AB | 8 | 6,931 | 3,191,289 | OR | 0.97(0.86,1.10) | 0.6443 | 0.00% | 0.41 | 0.97(0.86,1.10) | 0.97(0.86,1.10) |
| Zhang BL,2015 | Blood group A | 8 | 6,931 | 3,214,941 | OR | 1.03(0.96,1.12) | 0.3794 | 43.35% | 0.07 | 1.04(0.96,1.12) | 1.02(0.92,1.13) |
| Zhang BL,2015 | Blood group B | 8 | 6,931 | 3,193,532 | OR | 1.01(0.93,1.09) | 0.8975 | 0.00% | 0.40 | 1.01(0.93,1.09) | 1.01(0.93,1.09) |
| Naing C,2017 | Telomere Length | 8 | 951 | 2,569 | OR | 1.01(0.77,1.34) | 0.9270 | 30.40% | 0.24 | 1.01(0.77,1.34) | 1.01(0.77,1.34) |
| Jiang R,2016 | Enterolactone | 3 | 762 | 2,408 | RR | 1.11(0.93, 1.32) | 0.2668 | 44.60% | 0.06 | 1.11(0.93,1.32) | 1.12(0.92,1.36) |
| **Protein& amino acids** | | | | | | | | | | | |
| Lu S,2017 | Total Adiponectin | 8 | 3,420 | 8,937 | RR | 0.79(0.68,0.92) | 0.0024 | 5.37% | 0.35 | 0.79(0.68,0.92) | 0.79(0.68,0.92) |
| Yang G,2016 | Resistin | 11 | 965 | 2,290 | SMD | 0.65(0.24,1.05) | 0.0016 | 93.12% | 0.94 | 0.65(0.24,1.05) | 0.65(0.19,1.10) |
| Shiao SPK,2018 | Homocysteine | 8 | 4,047 | 9,604 | SMD | 0.13(0.03, 0.22) | 0.0072 | 74.80% | 0.73 | 0.13(-0.04,0.29) | 0.12(0.05,0.20) |
| Yu DH,2018 | Angiogenin | 2 | 188 | 240 | SMD | 1.53(0.49,2.58) | 0.0043 | 88.33% | / | 1.53(0.48,2.58) | 1.53(0.48,2.58) |
| Xing XJ,2014 | MMP7 | 10 | 625 | 1,020 | SMD | 2.15(1.46,2.84) | 9.65E-10 | 94.65% | 0.00 | 2.15(0.90,3.40) | 2.31(0.90,3.72) |
| Li XX,2014 | TLR-4 protein | 3 | 168 | 283 | OR | 4.75(1.16,19.37) | 0.0300 | 77.43% | 0.00 | 4.75(1.16,19.37) | 4.76(1.14,19.83) |
| Sun SJ,2016 | HER‑2(human epidermal growth factor receptor 2) expression | 13 | 932 | 1,453 | OR | 10.43(5.48,19.89) | 8.77E-11 | 63.02% | 0.00 | 10.43(5.48,19.88) | 11.33(5.44,23.59) |
| Feng Z, 2015 | Ferritin | 7 | 277 | 927 | SMD | -1.56(-2.70, -0.41) | 0.0079 | 97.47% | 0.00 | -1.56(-2.70, -0.41) | -1.57(-3.06, -0.08) |
| Ouyang Z,2017 | CD26 | 9 | 952 | 1,809 | SMD | -0.33(-2.97,2.30) | 0.8033 | 99.63% | 0.27 | -0.34(-5.11,4.44) |  |
| Gialamas SP,2013 | Leptin | 23 | 3,508 | 7,478 | SMD | 0.18(-0.04,0.40) | 0.1094 | 94.01% | 0.41 | 0.18(-0.22,0.58) | 0.20(-0.32,0.72) |
| CRC: colorectal cancer, HKSJ: Hartung-Knapp-Sidik-Jonkman, 1PEgger: The P value for small study effect test, 2Psig: The P value for excess significance test, RR: risk ratio, SMD: standard mean difference, OR: odds ratio, r: standardized correlation coefficient, LC n-3 PUFA: long chain n-3 polyunsaturated fatty acid, EPA: Eicosapentaenoic acid, DHA: Docosahexaenoic acid, DPA: Docosapentaenoic acid, HDL: high-density lipoprotein cholesterol, LDL: low-density lipoprotein cholesterol, HPV: Human papillomavirus, CRP: C-reactive protein, IL-6: Interleukin 6, HOMA-IR: homeostatic model assessment-insulin resistance, IGF 1/2: Insulin-like growth factor 1/2, IGFBP 1/2/3: Insulin-like growth factor-binding protein 1/2/3, HbA1c: glycated hemoglobin, MMP7: matrix metalloproteinase-7, CD26: dipeptidyl peptidase IV. | | | | | | | | | | | |

| **Supplementary Table 7 (Continued)** | | | | | | | | | |
| --- | --- | --- | --- | --- | --- | --- | --- | --- | --- |
| Biomarker | P, PM | I^2^, PM | P_Egger_, PM | 95%PI, PM | Effect size, HKSJ (95%CI) | P, HKSJ | I^2^, HKSJ | P_Egger_, HKSJ | 95%PI, HKSJ |
| **Fatty acid/Lipid metabolism biomarkers** | | | | | | | | | |
| LC n-3 PUFA | 0.0042 | 0.00% | 0.78 | 0.58(0.40,0.84) | 0.58(0.40,0.84) | 0.0042 | 0.21% | 0.78 | 0.58(0.40,0.84) |
| Biospecimen EPA 20:5 (n-3) | 0.0242 | 0.00% | 0.48 | 0.64(0.43,0.94) | 0.64(0.42,0.96) | 0.0314 | 9.24% | 0.50 | 0.64(0.42,0.96) |
| Biospecimen DHA 22:6 (n-3) | 0.0142 | 30.68% | 0.12 | 0.53(0.32,0.88) | 0.51(0.28,0.93) | 0.0266 | 46.59% | 0.19 | 0.51(0.28,0.93) |
| Biospecimen DPA 22:5 (n-3) | 0.0458 | 17.69% | 0.44 | 0.57(0.33,0.99) | 0.56(0.29,1.07) | 0.0788 | 37.89% | 0.46 | 0.56(0.30,1.07) |
| Blood EPA 20:5 (n-3) | 0.3104 | 98.89% | 0.01 | -0.98(-2.89,0.92) | -0.98(-2.87,0.91) | 0.3080 | 98.87% | 0.01 | -0.98(-2.87,0.91) |
| Blood DHA 22:6 (n-3) | 0.3098 | 0.00% | 0.31 | -0.09(-0.27,0.09) | -0.07(-0.31,0.17) | 0.5718 | 35.10% | 0.41 | -0.07(-0.31,0.17) |
| Blood DPA 22:5 (n-3) | 0.2293 | 92.11% | 0.00 | 0.43(-0.27,1.12) | 0.43(-0.27,1.12) | 0.2292 | 92.11% | 0.00 | 0.43(-0.27,1.12) |
| Adipose EPA 20:5 (n-3) | 0.6024 | 0.00% | / | 0.07(-0.20,0.35) | 0.07(-0.21,0.36) | 0.6083 | 3.39% | / | 0.07(-0.21,0.36) |
| Adipose DHA 22:6 (n-3) | 0.8920 | 26.74% | / | -0.02(-0.35,0.30) | -0.02(-0.37,0.32) | 0.8999 | 35.64% | / | -0.02(-0.37,0.32) |
| Adipose DPA 22:5 (n-3) | 0.4081 | 0.00% | / | 0.12(-0.16,0.39) | 0.12(-0.16,0.39) | 0.4082 | 0.07% | / | 0.12(-0.16,0.40) |
| HDL | 0.0727 | 40.66% | 0.40 | 0.84(0.69,1.02) | 0.83(0.67,1.04) | 0.1043 | 53.08% | 0.39 | 0.83(0.67,1.04) |
| LDL | 0.8909 | 85.73% | 0.15 | 1.04(0.57,1.92) | 1.04(0.57,1.92) | 0.8908 | 85.67% | 0.16 | 1.04(0.57,1.92) |
| Total cholesterol | 0.0688 | 66.89% | 0.57 | 1.11(0.99,1.25) | 1.11(0.98,1.27) | 0.1043 | 76.10% | 0.60 | 1.12(0.98,1.27) |
| Triglyceride | 0.0239 | 55.92% | 0.20 | 1.17(1.02,1.35) | 1.17(0.99,1.36) | 0.0598 | 47.77% | 0.22 | 1.17(0.99,1.37) |
| **Infectious agents** | | | | | | | | | |
| HPV | 2.41E-06 | 41.28% | 0.15 | 2.93(1.87,4.57) | 3.52(1.77,7.00) | 0.0003 | 75.66% | 0.6453 | 3.52(1.77,7.00) |
| Human cytomegalovirus infection | 2.91E-21 | 0.00% | 0.73 | 6.47(4.40,9.52) | 6.47(4.23,9.89) | 6.78E-18 | 13.74% | 0.70 | 6.47(4.23,9.89) |
| Streptococcus bovis | 2.03E-11 | 0.00% | 0.51 | 9.68(4.99,18.81) | 9.44(4.43,20.11) | 5.95E-09 | 18.72% | 0.57 | 9.44(4.43,20.11) |
| H.pylori | 0.0277 | 78.87% | 0.88 | 1.38(1.04,1.84) | 1.38(1.01,1.89) | 4.00E-02 | 82.00% | 0.90 | 1.38(1.02,1.89) |
| F. nucleatum | 0.0005 | 34.95% | / | 0.38(0.17,0.55) | 0.40(0.16,0.63) | 0.0010 | 40.05% | / | 0.38(0.16,0.56) |
| Enterobacteriaceae | 0.0133 | 88.74% | / | 2.62(0.55,4.69) | 2.62(0.62,4.61) | 0.0101 | 87.87% | / | 2.62(0.62,4.61) |
| Bifidobacterium | 0.0397 | 98.09% | 0.07 | -3.24(-6.33, -0.15) | -3.24(-6.32, -0.16) | 0.0390 | 98.08% | 0.07 | -3.24(-6.32,-0.16) |
| Faecalibacterium prausnitzii | 0.0192 | 0.00% | 0.63 | -0.32(-0.59,-0.05) | -0.31(-0.69,0.06) | 0.1000 | 25.76% | 0.60 | -0.31(-0.69,0.06) |
| Total bacteria | 0.5842 | 87.64% | 0.05 | 0.29(-0.75,1.32) | 0.29(-0.74,1.31) | 0.5825 | 87.31% | 0.07 | 0.29(-0.74,1.31) |
| Streptococcus bovis in faeces | 0.1457 | 56.38% | 0.18 | 3.10(0.68,14.20) | 3.10(0.68,14.20) | 0.1456 | 56.37% | 0.23 | 3.10(0.68,14.20) |
| Lactobacillus | 0.3014 | 99.15% | 0.19 | -1.85(-5.35,1.66) | -1.85(-5.33,1.64) | 0.2993 | 99.15% | 0.19 | -1.85(-5.33,1.64) |
| Bacteroides-Prevotella group | 0.4072 | 95.38% | 0.84 | -0.70(-2.35,0.95) | -0.70(-2.33,0.94) | 0.4030 | 95.30% | 0.83 | -0.70(-2.33,0.94) |
| Escherichia coli | 0.3239 | 98.00% | / | 1.38(-1.36,4.12) | 1.38(-1.34,4.10) | 0.3195 | 97.96% | / | 1.38(-1.34,4.10) |
| **Inflammatory Markers** | | | | | | | | | |
| CRP | 0.0031 | 65.11% | 0.03 | 1.13(1.04,1.23) | 1.14(1.04,1.25) | 0.0059 | 72.80% | 0.05 | 1.14(1.04,1.25) |
| IL-6 | 0.4029 | 35.06% | 0.11 | 1.10(0.88,1.36) | 1.09(0.85,1.39) | 0.4959 | 47.99% | 0.19 | 1.09(0.85,1.39) |
| **Insulin related biomarkers** | | | | | | | | | |
| Fasting glucose | 9.60E-05 | 42.10% | 0.00 | 1.23(1.11,1.36) | 1.27(1.11,1.45) | 0.0006 | 66.17% | 0.0106 | 1.27(1.11,1.45) |
| HOMA-IR | 5.76E-05 | 20.89% | 0.10 | 1.52(1.24,1.86) | 1.56(1.22,1.98) | 0.0003 | 38.91% | 0.18 | 1.56(1.22,1.98) |
| Fasting insulin | 1.28E-04 | 0.00% | 0.70 | 1.42(1.19,1.69) | 1.40(1.12,1.74) | 0.0031 | 24.65% | 0.86 | 1.40(1.12,1.74) |
| IGF 1 | 0.0069 | 23.68% | 0.18 | 1.28(1.07,1.54) | 1.31(1.05,1.63) | 0.0187 | 48.42% | 0.28 | 1.31(1.05,1.63) |
| IGF 2 | 0.0153 | 16.80% | 0.65 | 1.52(1.08,2.12) | 1.52(0.99,2.34) | 0.0549 | 42.30% | 0.62 | 1.52(0.99,2.34) |
| IGFBP 1 | 0.1056 | 4.90% | 0.26 | 0.84(0.69,1.04) | 0.81(0.61,1.08) | 0.1585 | 43.13% | 0.42 | 0.81(0.61,1.09) |
| IGFBP 2 | 0.4008 | 68.29% | 0.01 | 0.77(0.41,1.43) | 0.76(0.41,1.43) | 0.4016 | 68.77% | 0.02 | 0.77(0.41,1.43) |
| IGFBP 3 | 0.2844 | 51.93% | 0.32 | 0.88(0.69,1.11) | 0.88(0.70,1.10) | 0.2680 | 45.74% | 0.31 | 0.88 0.71 1.10 |
| HbA1c | 0.0814 | 89.56% | 0.31 | 1.23(0.97,1.56) | 1.25(0.93,1.67) | 0.1414 | 53.83% | 0.30 | 1.25(0.93,1.67) |
| C-peptide | 0.0514 | 60.90% | 0.63 | 1.34(0.99,1.79) | 1.35(0.97,1.89) | 0.0788 | 70.63% | 0.73 | 1.35(0.97,1.89) |
| **Micronutrients** | | | | | | | | | |
| 25-hydroxyvitamin D | 2.87E-05 | 0.00% | 0.61 | 0.68(0.57,0.82) | 0.67(0.54,0.83) | 0.0002 | 20.49% | 0.79 | 0.67(0.54,0.83) |
| 1,25-dihydroxyvitamin D | 0.9443 | 34.36% | 0.64 | 1.02(0.67,1.53) | 1.02(0.66,1.59) | 0.9302 | 42.59% | 0.64 | 1.02(0.66,1.59) |
| Vitamin B2 | 0.0069 | 0.00% | / | 0.74(0.59,0.92) | 0.74(0.57,0.95) | 0.0200 | 9.32% | / | 0.74(0.58,0.95) |
| Vitamin B6 | 4.75E-05 | 0.00% | 0.97 | 0.52(0.38,0.71) | 0.52(0.38,0.71) | 0.0012 | 0.89% | 0.97 | 0.52(0.38,0.72) |
| Vitamin B12 | 0.0397 | 39.77% | 0.00 | -0.06(-0.16,0.04) | -0.07(-0.14, -0.003) | 0.0384 | 54.91% | 0.03 | -0.07(-0.19,0.05) |
| Folate | 0.0097 | 99.56% | 0.04 | -1.29(-3.06,0.48) | -1.29(-2.29, -0.30) | 0.0105 | 99.57% | 0.04 | -1.29(-3.09,0.51) |
| Vitamin E | 0.0650 | 96.62% | 0.01 | -0.79(-1.63,0.05) | -0.79(-1.63,0.05) | 0.0650 | 96.61% | 0.01 | -0.79(-1.63,0.05) |
| Selenium | 0.1585 | 0.00% | 0.84 | 0.86(0.70,1.06) | 0.86(0.62,1.18) | 0.3509 | 42.92% | 0.7895 | 0.86(0.62,1.18) |
| Serum Zinc | 0.9788 | 99.44% | 0.73 | 0.05(-3.47,3.56) | 0.05(-3.47,3.56) | 0.9788 | 99.40% | 0.73 | 0.05(-3.47,3.56) |
| Tissue Zinc | 0.5236 | 98.52% | 0.00 | -0.70(-2.84,1.45) | -0.76(-3.08,1.55) | 0.5187 | 98.75% | 0.00 | -0.76(-3.08,1.55) |
| **Other Biomarkers** | | | | | | | | | |
| Blood group O | 5.78E-05 | 0.00% | 0.22 | 0.90(0.86,0.95) | 0.91(0.84,0.99) | 0.0445 | 54.58% | 0.23 | 0.91(0.84,0.99) |
| Blood group AB | 0.6443 | 0.00% | 0.41 | 0.97(0.86,1.10) | 0.94(0.73,1.21) | 0.6416 | 61.18% | 0.44 | 0.94(0.73,1.21) |
| Blood group A | 0.6917 | 65.94% | 0.08 | 1.02(0.92,1.13) | 1.01(0.90,1.14) | 0.8533 | 75.87% | 0.12 | 1.01(0.90,1.14) |
| Blood group B | 0.8975 | 0.00% | 0.40 | 1.01(0.93,1.09) | 1.00(0.91,1.10) | 0.9963 | 18.12% | 0.41 | 1.00(0.91,1.10) |
| Telomere Length | 0.9272 | 30.34% | 0.26 | 1.01(0.77,1.34) | 1.02(0.75,1.40) | 0.8842 | 42.82% | 0.34 | 1.02(0.75,1.40) |
| Enterolactone | 0.2710 | 51.36% | 0.06 | 1.12(0.92,1.36) | 1.14(0.89,1.47) | 0.2924 | 66.71% | 0.06 | 1.14(0.89,1.47) |
| **Protein& amino acids** | | | | | | | | | |
| Total Adiponectin | 0.0025 | 6.14% | 0.35 | 0.79(0.68,0.92) | 0.78(0.65,0.95) | 0.0143 | 39.81% | 0.38 | 0.79(0.65,0.95) |
| Resistin | 0.0059 | 94.79% | 0.94 | 0.65(0.19,1.11) | 0.65(0.19,1.11) | 0.0059 | 94.79% | 0.94 | 0.65(0.19,1.11) |
| Homocysteine | 2.10E-03 | 65.62% | 0.68 | 0.13(-0.02, 0.27) | 0.13(0.04,0.21) | 0.0030 | 68.35% | 0.69 | 0.13(-0.03,0.28) |
| Angiogenin | 0.0043 | 88.33% | / | 1.53(0.48,2.58) | 1.53(0.52,2.54) | 0.0030 | 87.41% | / | 1.53(0.52,2.54) |
| MMP7 | 0.0013 | 98.80% | 0.00 | 2.31(-0.24, 4.86) | 2.31(0.91,3.71) | 0.0013 | 98.80% | 0.00 | 2.31(-0.24,4.86) |
| TLR-4 protein | 0.0320 | 78.07% | 0.00 | 4.76(1.14, 19.83) | 4.75(1.16,19.45) | 0.0304 | 77.55% | 0.01 | 4.75(1.16,19.46) |
| HER‑2(human epidermal growth factor receptor 2) expression | 8.77E-11 | 71.76% | 0.00 | 11.33(5.44,23.59) | 11.82(5.36,26.08) | 9.33E-10 | 63.02% | 0.00 | 11.82(5.36,26.08) |
| Ferritin | 0.0391 | 98.51% | 0.00 | -1.57(-3.06, -0.08) | -1.57(-3.06, -0.08) | 0.0388 | 98.50% | 0.00 | -1.57(-3.06, -0.08) |
| CD26 | / | / | / | / | -0.33(-4.35,3.70) | 0.8737 | 99.84% | 0.45 | -0.33(-7.62,6.97) |
| Leptin | 0.4544 | 99.03% | 0.58 | 0.20(-0.75,1.14) | 0.20(-0.32,0.72) | 0.4551 | 99.03% | 0.58 | 0.20(-0.75,1.14) |

| **Supplementary Table 8 Meta-analyses of RCTs on supplementary micronutrients and CRC risk** | | | | | | | | | | | | | |
| --- | --- | --- | --- | --- | --- | --- | --- | --- | --- | --- | --- | --- | --- |
| First author, year | Ethicity | Population | Biomarker proxies | Dose | Comparison | Duration | No of studies | No of event | No of participants | Metric | Model | Effect size (95%CI) | I2(%) |
| Arain MA,2010^92^ | / | Healthy people aged over 40 years old | Vitamin E | 50mg/day; 400IU/day; 600IU/second day | Placebo | 7-10 years | 4 | 574 | 94,069 | RR | Fixed | 0.89(0.76,1.05) | 7% |
| Bjelakovic G,2014^93^ | / | Healthy people or with low-trauma, osteoporotic or fractureisolated systolic hypertension aged over 50 years old | Vitamin D3 (Cholecalciferol) | 800IU/day; 800 IU plus calcium 1000 mg daily; 400 IU plus calcium 1000 mg daily; 1000IU/day plus calcium 1400 to 1500 mg daily; 100,000 IU/4 months; 100,000 IU oral vitamin D/3-monthly | Placebo or no intervention | 1-7 years | 5 | 436 | 45,598 | RR | Random | 1.11(0.92,1.34) | 0% |
| Bristow SM,2013^94^ | / | Healthy people aged over 40 years old | Calcium | >500 mg/d | Placebo | 2-5 years | 8 | 83 | 9,863 | RR | Random | 1.38(0.89,2.15) | 0% |
| Druesne-Pecollo N,2010^95^ | / | Smokers or asbestos workers, or not | Beta-carotene given singly or in combination with other antioxidants | 6–15 mg/day or 20–30 mg/day | Placebo | 4-25 years | 7 | 957 | 151,118 | HR | Fixed | 0.96 (0.85,1.09) | / |
| Qin T,2015^96^ | Mixed | Vascular disease, diabetes, colorectal adenoma patients or healthy people aged over 57 years old | Folic acid | 0.5-2.5mg/day | Placebo | 27-88 months | 8 | 381 | 34,598 | RR | Fixed | 1.00(0.82,1.22) | 0% |
| Qin X,2013^97^ | Mixed | Vascular disease, diabetes, colorectal adenoma patients or healthy people aged over 57 years old | Folic acid | 0.5-2.5mg/day | Placebo | 36-88 months | 7 | 377 | 33,824 | RR | Random | 1.01(0.82,1.23) | / |
| RR: risk ratio; HR: hazard ratio | | | | | | | | | | | | | |

**References**

1 Yang B, Wang FL, Ren XL, Li D. Biospecimen long-chain N-3 PUFA and risk of colorectal cancer: A meta-analysis of data from 60,627 individuals. *PLoS One* 2014;9:e110574.

2 Esposito K, Chiodini P, Capuano A, et al. Colorectal cancer association with metabolic syndrome and its components: A systematic review with meta-analysis. *Endocrine* 2013;44:634-47.

3 Yao X, Tian Z. Dyslipidemia and colorectal cancer risk: a meta-analysis of prospective studies. *Cancer Causes Control* 2015;26:257-68.

4 Repass J, Iorns E, Denis A, Williams SR, Perfito N, Errington TM. Replication study: Fusobacterium nucleatum infection is prevalent in human colorectal carcinoma. *Elife* 2018;13:e25801.

5 Liu H, Wu H, Bilegsaikhan E, Lu EX, Shen X, Liu T. Differential expression of intestinal microbiota in colorectal cancer compared with healthy controls: A systematic review and meta-analysis. *Int J Clin Exp Med* 2016;9:10923-30.

6 Wang F, Sun MY, Shi SL, Lv ZS. Helicobacter pylori infection and normal colorectal mucosa-adenomatous polyp-adenocarcinoma sequence: a meta-analysis of 27 case-control studies. *Colorectal Dis* 2014;16:246-52.

7 Wang X, Wang G, Zhang L. Relationship between helicobacter pylori infection and colorectal cancer: A meta-analysis of observational studies. *Int J Clin Exp Med* 2017;10:11402-08.

8 Zhao Y, Wang X, Wang Y. Helicobacter pylori infection and colorectal carcinoma risk: A meta-analysis. *J Cancer Res Ther* 2016;12:C15-C18.

9 Wu Q, Yang ZP, Xu P, Gao LC, Fan DM. Association between Helicobacter pylori infection and the risk of colorectal neoplasia: A systematic review and meta-analysis. *Colorectal Dis* 2013;15:e352-e64.

10 Rokkas T, Sechopoulos P, Pistiolas D, Kothonas F, Margantinis G, Koukoulis G. The relationship of Helicobacter pylori infection and colon neoplasia, on the basis of meta-analysis. *Eur J Gastroenterol Hepatol* 2013;25:1286-94.

11 Guo Y, Li HY. Association between Helicobacter pylori infection and colorectal neoplasm risk: A meta-analysis Based on East Asian population. *J Cancer Res Ther* 2014;10:C263-C66.

12 Zhao Y-s, Wang F, Chang D, Han B, You D-y. Meta-analysis of different test indicators: Helicobacter pylori infection and the risk of colorectal cancer. *Int J Colorectal Dis* 2008;23:875-82.

13 Baandrup L, Thomsen LT, Olesen TB, Andersen KK, Norrild B, Kjaer SK. The prevalence of human papillomavirus in colorectal adenomas and adenocarcinomas: a systematic review and meta-analysis. *Eur J Cancer* 2014;50:1446-61.

14 Damin DC, Ziegelmann PK, Damin AP. Human papillomavirus infection and colorectal cancer risk: A Meta-analysis. *Colorectal Dis* 2013;15:e420-e28.

15 Pelizzer T, Dias CP, Poeta J, Torriani T, Roncada C. Colorectal cancer prevalence linked to human papillomavirus: a systematic review with meta-analysis. *Rev Bras Epidemiol* 2016;19:791-802.

16 Zhang XH, Wang W, Wang YQ, Jia DF, Zhu L. Human papillomavirus infection and colorectal cancer in the Chinese population: a meta-analysis. *Colorectal Dis* 2018;20:961-69.

17 Peder LD, Silva CM, Boeira VL, et al. Association between Human Papillomavirus and Non-cervical Genital Cancers in Brazil: A Systematic Review and Meta-Analysis. *Asian Pac J Cancer Prev* 2018;19:2359-71.

18 Ibragimova MK, Tsyganov MM, Litviakov NV. Human papillomavirus and colorectal cancer. *Med Oncol* 2018;35:140.

19 Bai B, Wang X, Chen E, Zhu H. Human cytomegalovirus infection and colorectal cancer risk: a meta-analysis. *Oncotarget* 2016;7:76735-42.

20 Liu C, Zheng P. The relationship of helicobacter pylori infection and the risk of colon neoplasia based on meta-analysis. *Int J Clin Exp Med* 2016;9:2293-300.

21 Boleij A, Van Gelder MMHJ, Swinkels DW, Tjalsma H. Clinical importance of streptococcus gallolyticus infection among colorectal cancer patients: Systematic review and meta-analysis. *Clin Infect Dis* 2011;53:870-78.

22 Krishnan S, Eslick GD. Streptococcus bovis infection and colorectal neoplasia: A meta-analysis. *Colorectal Dis* 2014;16:672-80.

23 Guo YZ, Pan L, Du CJ, Ren DQ, Xie XM. Association between C-reactive protein and risk of cancer: A meta-analysis of prospective cohort studies. *Asian Pac J Cancer Prev* 2013;14:243-48.

24 Zhou B, Shu B, Yang J, Liu J, Xi T, Xing Y. C-reactive protein, interleukin-6 and the risk of colorectal cancer: a meta-analysis. *Cancer Causes Control* 2014;25:1397-405.

25 Tsilidis KK, Branchini C, Guallar E, Helzlsouer KJ, Erlinger TP, Platz EA. C‐reactive protein and colorectal cancer risk: A systematic review of prospective studies. *Int J Cancer* 2008;123:1133-40.

26 Kakourou A, Koutsioumpa C, Lopez DS, et al. Interleukin-6 and risk of colorectal cancer: results from the CLUE II cohort and a meta-analysis of prospective studies. *Cancer Causes Control* 2015;26:1449-60.

27 Xu J, Ye Y, Wu H, et al. Association between markers of glucose metabolism and risk of colorectal cancer. *BMJ Open* 2016;6 e011430.

28 Pisani P. Hyper-insulinaemia and cancer, meta-analyses of epidemiological studies. *Arch Physiol Biochem* 2008;114:63-70.

29 Chen L, Li L, Wang Y, et al. Circulating C-peptide level is a predictive factor for colorectal neoplasia: Evidence from the meta-analysis of prospective studies. *Cancer Causes Control* 2013;24:1837-47.

30 Shi J, Xiong L, Li J, et al. A Linear Dose-Response Relationship between Fasting Plasma Glucose and Colorectal Cancer Risk: Systematic Review and Meta-analysis. *Sci Rep* 2015;5:17591.

31 Crawley DJ, Holmberg L, Melvin JC, et al. Serum glucose and risk of cancer: A meta-analysis. *BMC Cancer* 2014;14:985.

32 Chi F, Wu R, Zeng YC, Xing R, Liu Y. Circulation insulin-like growth factor peptides and colorectal cancer risk: an updated systematic review and meta-analysis. *Mol Biol Rep* 2013;40:3583-90.

33 Rinaldi S, Cleveland R, Norat T, et al. Serum levels of IGF-I, IGFBP-3 and colorectal cancer risk: Results from the EPIC cohort, plus a meta-analysis of prospective studies. *Int J Cancer* 2010;126:1702-15.

34 Morris J, George L, Wu T, Wald N. Insulin-like growth factors and cancer: no role in screening. Evidence from the BUPA study and meta-analysis of prospective epidemiological studies. *Br J Cancer* 2006;95:112.

35 Chuang SC, Rota M, Gunter MJ, et al. Quantifying the dose-response relationship between circulating folate concentrations and colorectal cancer in cohort studies: A meta-analysis based on a flexible meta-regression model. *Am J Epidemiol* 2013;178:1028-37.

36 Shiao SPK, Lie A, Yu CH. Meta-analysis of homocysteine-related factors on the risk of colorectal cancer. *Oncotarget* 2018;9:25681-97.

37 Moazzen S, Dolatkhah R, Tabrizi JS, et al. Folic acid intake and folate status and colorectal cancer risk: A systematic review and meta-analysis. *Clin Nutr* 2017;37:1926-34.

38 Zhang D, Wen X, Wu W, Guo Y, Cui W. Elevated homocysteine level and folate deficiency associated with increased overall risk of carcinogenesis: meta-analysis of 83 case-control studies involving 35,758 individuals. *PLoS One* 2015;10:e0123423.

39 Sun NH, Huang XZ, Wang SB, et al. A dose-response meta-analysis reveals an association between vitamin B12 and colorectal cancer risk. *Public Health Nutr* 2016;19:1446-56.

40 Ben S, Du M, Ma G, et al. Vitamin B2 intake reduces the risk for colorectal cancer: a dose-response analysis. *Eur J Nutr* 2018;58:1591-602.

41 Larsson SC, Orsini N, Wolk A. Vitamin B6 and risk of colorectal cancer: a meta-analysis of prospective studies. *JAMA* 2010;303:1077-83.

42 Mocellin S, Briarava M, Pilati P. Vitamin B6 and cancer risk: A field synopsis and meta-analysis. *J Natl Cancer Inst* 2017;109:1-9.

43 Vinceti M, Dennert G, Crespi CM, et al. Selenium for preventing cancer. *Cochrane Database Syst Rev* 2014;2014:CD005195.

44 Vinceti M, Filippini T, Del Giovane C, et al. Selenium for preventing cancer. *Cochrane Database Syst Rev* 2018.

45 Chung M, Lee J, Terasawa T, Lau J, Trikalinos TA. Vitamin D with or without calcium supplementation for prevention of cancer and fractures: an updated meta-analysis for the U.S. Preventive Services Task Force. *Ann Intern Med* 2011;155:827-38.

46 Ekmekcioglu C, Haluza D, Kundi M. 25-Hydroxyvitamin D Status and Risk for Colorectal Cancer and Type 2 Diabetes Mellitus: A Systematic Review and Meta-Analysis of Epidemiological Studies. *Int J Environ Res Public Health* 2017;14:28.

47 Gandini S, Boniol M, Haukka J, et al. Meta-analysis of observational studies of serum 25-hydroxyvitamin D levels and colorectal, breast and prostate cancer and colorectal adenoma. *Int J Cancer* 2011;128:1414-24.

48 Garland CF, Gorham ED. Dose-response of serum 25-hydroxyvitamin D in association with risk of colorectal cancer: A meta-analysis. *J Steroid Biochem Mol Biol* 2017;168:1-8.

49 Ma Y, Zhang P, Wang F, Yang J, Liu Z, Qin H. Association between vitamin D and risk of colorectal cancer: A systematic review of prospective studies. *J Clin Oncol* 2011;29:3775-82.

50 Touvier M, Chan DSM, Lau R, et al. Meta-analyses of vitamin D intake, 25-hydroxyvitamin D status, vitamin D receptor polymorphisms, and colorectal cancer risk. *Cancer Epidemiol Biomarkers Prev* 2011;20:1003-16.

51 Lee JE, Li H, Chan AT, et al. Circulating levels of vitamin D and colon and rectal cancer: The Physicians' Health Study and a meta-analysis of prospective studies. *Cancer Prev Res (Phila)* 2011;4:735-43.

52 Yin L, Grandi N, Raum E, Haug U, Arndt V, Brenner H. Meta‐analysis: longitudinal studies of serum vitamin D and colorectal cancer risk. *Aliment Pharmacol Ther* 2009;30:113-25.

53 Dong Y, Liu Y, Shu Y, et al. Link between risk of colorectal cancer and serum vitamin E levels: A meta-analysis of case-control studies. *Medicine (Baltimore)* 2017;96:e7470.

54 Gumulec J, Masarik M, Adam V, Eckschlager T, Provaznik I, Kizek R. Serum and tissue zinc in epithelial malignancies: A meta-analysis. *PLoS One* 2014;9 e99790.

55 Jiang R, Botma A, Rudolph A, Husing A, Chang-Claude J. Phyto-oestrogens and colorectal cancer risk: a systematic review and dose-response meta-analysis of observational studies. *Br J Nutr* 2016;116:2115-28.

56 Zhang BL, He N, Huang YB, Song FJ, Chen KX. ABO blood groups and risk of cancer: a systematic review and meta-analysis. *Asian Pac J Cancer Prev* 2014;15:4643-50.

57 Zhang X, Zhao Q, Zhu W, et al. The association of telomere length in peripheral blood cells with cancer risk: A Systematic review and meta-Analysis of prospective studies. *Cancer Epidemiol Biomarkers Prev* 2017;26:1381-90.

58 Naing C, Aung K, Lai PK, Mak JW. Association between telomere length and the risk of colorectal cancer: A meta-analysis of observational studies. *BMC Cancer* 2017;17:24.

59 Lu S, Hua H, Wu M, Xiang T, Cheng X. Circulating adiponectin level and risk of colorectal cancer: Evidence from a dose-response meta-analysis. *Int J Clin Exp Med* 2017;10:13015-24.

60 Lu W, Huang Z, Li N, Liu H. Low circulating total adiponectin, especially its non-high-molecular weight fraction, represents a promising risk factor for colorectal cancer: A meta-analysis. *Onco Targets Ther* 2018;11:2519-31.

61 Xu XT, Xu Q, Tong JL, et al. Meta-analysis: circulating adiponectin levels and risk of colorectal cancer and adenoma. *J Dig Dis* 2011;12:234-44.

62 Joshi RK, Lee SA. Obesity related adipokines and colorectal cancer: A review and meta-analysis. *Asian Pac J Cancer Prev* 2014;15:397-405.

63 An W, Bai Y, Deng SX, et al. Adiponectin levels in patients with colorectal cancer and adenoma: a meta-analysis. *Eur J Cancer Prev* 2012;21:126-33.

64 Yu D, Cai Y, Zhou W, Sheng J, Xu Z. The Potential of Angiogenin as a Serum Biomarker for Diseases: Systematic Review and Meta-Analysis. *Dis Markers* 2018;2018:1984718.

65 Yang G, Fan W, Luo B, et al. Circulating Resistin Levels and Risk of Colorectal Cancer: A Meta-Analysis. *Biomed Res Int* 2016;2016:7367485.

66 Gialamas SP, Sergentanis TN, Antonopoulos CN, Dessypris N, Chrousos GP, Petridou ET. Circulating leptin levels and risk of colorectal cancer and adenoma: a case-control study and meta-analysis. *Cancer Causes Control* 2013;24:2129-41.

67 Feng Z, Chen JW, Feng JH, et al. The association between serum ferritin with colorectal cancer. *Int J Clin Exp Med* 2015;8:22293-99.

68 Sun SJ, Lin Q, Sun Q, et al. High HER-2 protein levels correlate with clinicopathological features in colorectal cancer. *J Cancer Res Ther* 2016;12:323-33.

69 Xing XJ, Xiao-Hu G, Ma TF. Relationship of serum mmp-7 levels for colorectal cancer: A meta-analysis. *Tumour Biol* 2014;35:10515-22.

70 Li XX, Sun GP, Meng J, et al. Role of toll-like receptor 4 in colorectal carcinogenesis: A meta-analysis. *PLoS One* 2014;9:e93904.

71 Ouyang Z, Jiang Y, Deng F, Wang B. Serum CD26 levels may be correlated with the risk of colorectal cancer among asian populations: A meta-analysis. *Int J Clin Exp Med* 2017;10:8545-57.

72 He Y, Timofeeva M, Farrington SM, et al. Exploring causality in the association between circulating 25-hydroxyvitamin D and colorectal cancer risk: a large Mendelian randomisation study. *BMC Med* 2018;16:142.

73 Chandler PD. Association between vitamin D genetic risk score and cancer risk in a large cohort of U.S. women. *Nutrients* 2018;10:55.

74 Dimitrakopoulou V, Tsilidis KK, Haycock PC, et al. Circulating vitamin D concentration and risk of seven cancers: Mendelian randomisation study. *BMJ* 2017;359:j4761.

75 Theodoratou E, Palmer T, Zgaga L, et al. Instrumental variable estimation of the causal effect of plasma 25-hydroxy-vitamin D on colorectal cancer risk: a mendelian randomization analysis. *PLoS One* 2012;7:e37662.

76 Ong J-S, Gharahkhani P, An J, et al. Vitamin D and overall cancer risk and cancer mortality: a Mendelian randomization study. *Hum Mol Genet* 2018;27:4315-22.

77 Cornish AJ, Law PJ, Timofeeva M, et al. Modifiable pathways for colorectal cancer: A Mendelian randomisation analysis. *Lancet Gastroenterol Hepatol* 2019;5:55-62.

78 May-Wilson S, Sud A, Law PJ, et al. Pro-inflammatory fatty acid profile and colorectal cancer risk: A Mendelian randomisation analysis. *Eur J Cancer* 2017;84:228-38.

79 Liyanage UE, Ong JS, An J, Gharahkhani P, Law MH, MacGregor S. Mendelian Randomization Study for Genetically Predicted Polyunsaturated Fatty Acids Levels on Overall Cancer Risk and Mortality. *Cancer Epidemiol Biomark Prev* 2019;28:1015-23.

80 Rodriguez‐Broadbent H, Law PJ, Sud A, et al. Mendelian randomisation implicates hyperlipidaemia as a risk factor for colorectal cancer. *Int J Cancer* 2017;140:2701-08.

81 Orho-Melander M, Hindy G, Borgquist S, et al. Blood lipid genetic scores, the HMGCR gene and cancer risk: a Mendelian randomization study. *Int J Epidemiol* 2017;47:495-505.

82 Nimptsch K, Aleksandrova K, Boeing H, et al. Association of CRP genetic variants with blood concentrations of C-reactive protein and colorectal cancer risk. *Int J Cancer* 2015;136:1181-92.

83 Wang X, Dai JY, Albanes D, et al. Mendelian randomization analysis of C-reactive protein on colorectal cancer risk. *Int J Epidemiol* 2018;48:767-80.

84 Zhang C, Doherty JA, Burgess S, et al. Genetic determinants of telomere length and risk of common cancers: a Mendelian randomization study. *Hum Mol Genet* 2015;24:5356-66.

85 Haycock PC, Burgess S, Nounu A, et al. Association between telomere length and risk of cancer and non-neoplastic diseases: a Mendelian randomization study. *JAMA oncology* 2017;3:636-51.

86 Au Yeung SL, Luo S, Schooling CM. The impact of GDF-15, a biomarker for metformin, on the risk of coronary artery disease, breast and colorectal cancer, and type 2 diabetes and metabolic traits: a Mendelian randomisation study. *Diabetologia* 2019;62:1638-46.

87 Nimptsch K, Song M, Aleksandrova K, et al. Genetic variation in the ADIPOQ gene, adiponectin concentrations and risk of colorectal cancer: a Mendelian Randomization analysis using data from three large cohort studies. *Eur J Epidemiol* 2017;32:419-30.

88 Nimptsch K, Aleksandrova K, Boeing H, et al. Plasma fetuin-A concentration, genetic variation in the AHSG gene and risk of colorectal cancer. *Int J Cancer* 2015;137:911-20.

89 Morris J, George L, Wu T, Wald N. Insulin-like growth factors and cancer: no role in screening. Evidence from the BUPA study and meta-analysis of prospective epidemiological studies. *Br J Cancer* 2006;95:112.

90 Joshi RK, Kim WJ, Lee SA. Association between obesity-related adipokines and colorectal cancer: a case-control study and meta-analysis. *World J Gastroenterol* 2014;20:7941-49.

91 Song M, Gong J, Giovannucci EL, et al. Genetic variants of adiponectin and risk of colorectal cancer. *Int J Cancer* 2015;137:154-64.

92 Arain MA, Abdul Qadeer A. Systematic review on "vitamin E and prevention of colorectal cancer". *Pak* 2010;23:125-30.

93 Bjelakovic G, Gluud LL, Nikolova D, et al. Vitamin D supplementation for prevention of cancer in adults. *Cochrane Database Syst Rev* 2014:CD007469.

94 Bristow SM, Bolland MJ, MacLennan GS, et al. Calcium supplements and cancer risk: a meta-analysis of randomised controlled trials. *Br J Nutr* 2013;110:1384-93.

95 Druesne-Pecollo N, Latino-Martel P, Norat T, et al. Beta-carotene supplementation and cancer risk: a systematic review and metaanalysis of randomized controlled trials. *Int J Cancer* 2010;127:172-84.

96 Qin T, Du M, Du H, Shu Y, Wang M, Zhu L. Folic acid supplements and colorectal cancer risk: meta-analysis of randomized controlled trials. *Sci* 2015;5:12044.

97 Qin X, Cui Y, Shen L, et al. Folic acid supplementation and cancer risk: a meta-analysis of randomized controlled trials. *Int J Cancer* 2013;133:1033-41.
